# Kuwanon A from *Morus alba* L. Alleviates H_2_O_2_-Induced Oxidative Damage in HaCaT Keratinocytes by Inhibiting Ferroptosis and Enhancing Antioxidant Capacity

**DOI:** 10.3390/antiox15060657

**Published:** 2026-05-22

**Authors:** Yan Liu, Hening Fu, Junjie Ma, Youqing Wang, Zhaohua Shi, Yupeng Liu, Xianju Huang, Bingchen Han, Jun Li

**Affiliations:** 1 School of Pharmaceutical Science, South-Central Minzu University, Wuhan 430074, China; 2 College of Life Sciences, South-Central Minzu University, Wuhan 430074, China; 3 Hubei Shi-Zhen Laboratory, Wuhan 430065, China

**Keywords:** *Morus alba* L., Kuwanon A, oxidative stress, HaCaT, ferroptosis

## Abstract

The root bark of *Morus alba* L. is commonly used as a natural antioxidant; however, its active constituents and underlying molecular mechanisms remain unclear. In this study, a bioactivity-guided isolation approach was employed to identify antioxidant substances from the root bark of *Morus alba* L. and to investigate their protective effects against oxidative damage in HaCaT cells. Using techniques such as silica gel column chromatography and semi-preparative HPLC, combined with NMR and HR-ESI-MS analysis, 22 compounds were isolated and identified from the dichloromethane extract of *Morus alba* L. root bark, including Diels–Alder adducts, flavonoids, and benzofurans. Among them, compounds **1** and **2** are new compounds, while compounds **12** and **16** were isolated from this plant for the first time. Bioactivity screening revealed that Kuwanon A (compound **17**) exhibited significant cytoprotective effects in an H_2_O_2_-induced HaCaT cell injury model, effectively scavenging intracellular reactive oxygen species (ROS), restoring mitochondrial function, and enhancing the activities of antioxidant enzymes such as SOD and GSH. Further studies indicated that H_2_O_2_ induced ferroptosis in HaCaT cells, characterized by abnormal Fe^2+^ levels, lipid peroxidation, and elevated levels of pro-inflammatory cytokines (IL-1β, IL-6, TNF-α). Kuwanon A significantly ameliorated these pathological changes. Consistently, ELISA and Astral DIA quantitative proteomics analyses demonstrated that Kuwanon A specifically upregulates the expression of the sulfurtransferase NFS1, thereby promoting the expression of the core antioxidant enzyme GPX4 and the iron storage protein ferritin-H, collectively inhibiting ferroptosis. This study elucidates that Kuwanon A is a key active component responsible for the antioxidant and anti-inflammatory effects of *Morus alba* L. root bark, and its mechanism is closely associated with regulating the NFS1-mediated ferroptosis defense pathway.

## 1. Introduction

As the largest organ of the human body, the skin serves as the first line of defense against physical, chemical, and microbial external insults. However, with increasing environmental pollution, heightened ultraviolet radiation, and rising life stress, the skin is frequently exposed to high levels of oxidative stress, which in turn leads to a series of pathological processes such as impaired skin barrier function, inflammation, and photoaging [1,2]. Therefore, the search for efficient and low-toxicity natural active ingredients to prevent skin oxidative damage has been a research hotspot in the fields of dermatology and cosmetic science [3].

The dried root bark of *Morus alba* L., known as “Sang Baipi” in traditional Chinese medicine, is traditionally used for its effects of purging the lung to relieve dyspnea, promoting diuresis and reducing edema [4]. Modern pharmacological studies have shown that Sang Baipi exhibits various biological activities, including antibacterial, anti-inflammatory, hypoglycemic, and antioxidant effects [5,6,7,8,9]. To date, over a hundred compounds have been isolated from Sang Baipi, mainly including Diels–Alder adducts, stilbenes, flavonoids, phenylpropanoids, triterpenes, and alkaloids [10]. In recent years, due to its excellent antioxidant capacity, Sang Baipi has been widely used in the field of skin whitening and anti-aging, and various topical cosmetic formulations contain extracts of Sang Baipi [11]. Multiple studies have reported that Sang Baipi extract possesses significant antioxidant and tyrosinase inhibitory activities. Among the various flavonoids isolated from it, compounds such as 2′,4′-dihydroxy-7-methoxy-8-prenylflavan exert antioxidant effects by activating Nrf2, NQO1, and γ-GCS [12], while oxyresveratrol exhibits both antioxidant and tyrosinase inhibitory activities [13], and five compounds including trans-mulberroside A have been identified as competitive inhibitors of tyrosinase [14]. Therefore, searching for novel antioxidants from Sang Baipi for skin protection holds significant research value.

As a primary source of reactive oxygen species (ROS), hydrogen peroxide can directly induce oxidative damage in skin keratinocytes (HaCaT) [15]. In this study, modern column chromatography separation techniques and spectroscopic analysis methods were employed to isolate and identify the antioxidant active fractions from Sang Baipi. The isolated compounds were screened for activity using a hydrogen peroxide-induced HaCaT cell damage model, and the mechanism of action of the active compounds was preliminarily investigated using fluorescent probes, biochemical assay kits, Elisa kits, and proteomics.

## 2. Materials and Methods

### 2.1. Materials

Plant Material: The dried root bark of *Morus alba* L. was provided by Hubei Yaosheng Traditional Chinese Medicine Co., Ltd. (Wuhan, China). Chemicals and Chromatographic Materials: Unless otherwise specified, analytical-grade organic reagents were purchased from Sinopharm Chemical Reagent Co., Ltd. (Shanghai, China), and chromatographic-grade reagents were obtained from Hubei Fudun Science and Technology Co., Ltd. (Wuhan, China). Silica gel for column chromatography (200–300 mesh) was sourced from Qingdao Marine Chemical Co., Ltd. (Qingdao, China). ODS silica gel (50 μm) and a semi-preparative HPLC column (YMC-Pack ODS-A, 250 mm × 10 mm, 5 μm) were purchased from YMC Co., Ltd. (Kyoto, Japan). Cell Culture and Biochemical Reagents: DMEM medium, penicillin–streptomycin solution, and trypsin were purchased from HyClone (Logan, UT, USA). Fetal bovine serum (FBS) was obtained from Hangzhou Sijiqing Biological Engineering Materials Co., Ltd. (Hangzhou, China). The Cell Counting Kit-8 (CCK-8) was purchased from Beyotime Biotechnology Co., Ltd. (Shanghai, China). Assay Kits: Kits for superoxide dismutase (SOD), reduced glutathione (GSH), catalase (CAT), and malondialdehyde (MDA) were purchased from Nanjing Jiancheng Bioengineering Institute (Nanjing, China). Colorimetric assay kits for ferrous ion (Fe^2+^) and ferric ion (Fe^3+^), as well as enzyme-linked immunosorbent assay (ELISA) kits for human interleukin-6 (IL-6), tumor necrosis factor-α (TNF-α), interleukin-1β (IL-1β), glutathione peroxidase 4 (GPX4), transferrin receptor 1 (TFR1), and ferritin heavy chain (FTH), were purchased from Wuhan Enova Biotechnology Co., Ltd. (Wuhan, China).

### 2.2. Extraction and Isolation of Compounds

The dried root bark powder of *Morus alba* L. (6.67 kg) was refluxed with absolute ethanol three times (1 h each time). The combined filtrate was concentrated under reduced pressure to afford a total extract (660 g). The extract was suspended in water and successively partitioned with petroleum ether, CHCl_2_, EtOAc, and n-BuOH to obtain the corresponding fractions weighing 27.25 g, 52 g, 351.18 g, and 75.95 g, respectively.

The CHCl_2_ fraction (52 g) was subjected to silica gel column chromatography and eluted with a gradient of CHCl_2_–MeOH (100:1 to 50:50, *v*/*v*) to yield Fr.A and Fr.B. Fr.A (15 g) was further separated by reversed-phase C18 column chromatography (MeOH–H_2_O, 60:1) to afford subfractions A1–A3. These subfractions were purified by preparative HPLC as follows: A1 (ACN–H_2_O, 50:50) yielded compounds **14** (1.2 mg) and **15** (1 mg); A2 (ACN–H_2_O, 45:55) yielded compound **11** (3.6 mg); A3 (ACN–H_2_O, 35:65) yielded compounds **19** (17.1 mg) and 21 (6.7 mg).

Fr.B (26 g) was similarly separated by C18 column chromatography (MeOH–H_2_O, 70:1) to obtain subfractions B1–B7. Each subfraction was purified by preparative HPLC as follows: B1 (ACN–H_2_O, 85:15) gave compound **3** (2.4 mg); B2 (ACN–H_2_O, 70:30) gave compounds **8** (4 mg), **9** (14.9 mg), **16** (3 mg), and **20** (6.7 mg); B3 (ACN–H_2_O, 69:31) gave compounds 2* (2.8 mg), **5** (2 mg), and **22** (4.6 mg); B4 (ACN–H_2_O, 67:33) gave compounds 1* (2.3 mg), **6** (2 mg), **10** (5.1 mg), and **12** (2.6 mg); B5 (ACN–H_2_O, 58:42) gave compound **13** (9.3 mg); B6 (ACN–H_2_O, 53:47) gave compounds **4** (2.5 mg), **17** (8.8 mg), and **18** (16.4 mg); B7 (ACN–H_2_O, 15:85) gave compound **7** (10.8 mg).

### 2.3. Structural Identification of Compounds

NMR spectra were recorded on a Bruker AM-500 MHz spectrometer (Bruker, Karlsruhe, Germany), with TMS used as the internal reference. High-resolution ESI-MS data were acquired using a quadrupole ion trap high-resolution mass spectrometer (ThermoFisher Scientific, Waltham, MA, USA). CD spectra were measured on a Chirascan detector (Applied Photophysics Limited Shanghai Representative Office, Shanghai, China). Optical rotations were determined with a Rudolph Autopol IV polarimeter (Rudolph, Hackettstown, NJ, USA). UV full-wavelength scans were performed on a Shimadzu UV-2401 PC spectrophotometer (Shimadzu, Kyoto, Japan).

### 2.4. Cell Culture and Viability Assay (CCK-8 Method)

HaCaT cells were cultured in DMEM complete medium supplemented with 10% fetal bovine serum and 1% penicillin–streptomycin solution (a mixture of penicillin and streptomycin), at 37 °C in a 5% CO_2_ atmosphere.

Initial Screening of Drug Activity: HaCaT cells in the logarithmic growth phase were seeded into 96-well plates at a density of 5 × 10^3^ cells per well and incubated overnight until reaching approximately 80% confluence. In the experimental groups, hydrogen peroxide (H_2_O_2_) was added at a final concentration of 125 μM to induce cell injury, together with different candidate drugs at a final concentration of 20 μM. After co-incubation for 24 h, the detection solution was added strictly according to the instructions of the CCK-8 assay kit. Following an appropriate incubation period, the absorbance of each well was measured using a microplate reader to evaluate cell viability.

Cytotoxicity Assessment of Kuwanon A: HaCaT cells were seeded and cultured as described above until approximately 80% confluence. The old culture medium was discarded, and fresh medium containing various concentrations of Kuwanon A was added. After 24 h of incubation, cell viability was assessed using the CCK-8 method to evaluate the potential toxicity of Kuwanon A on normal HaCaT cells.

Protective Effect of Kuwanon A on H_2_O_2_-Damaged Cell Viability: HaCaT cells were seeded in 96-well plates and cultured overnight until approximately 80% confluence. Both the model group and the treatment groups were exposed to H_2_O_2_ at a final concentration of 125 μM, while the treatment groups were simultaneously treated with different concentrations of Kuwanon A. After co-incubation for 24 h, the relative cell viability was determined using the CCK-8 kit.

### 2.5. Laser Confocal Microscopy with Fluorescent Probe Detection

Mitochondrial Membrane Potential (JC-1) Assay: HaCaT cells in the logarithmic growth phase were seeded into 6-well plates at a density of 2 × 10^5^ cells per well and cultured overnight until reaching approximately 80% confluence. Subsequently, the cells were co-incubated with H_2_O_2_ at a final concentration of 125 μM and Kuwanon A at a concentration of 25 μM for 24 h. Probe incubation and washing were performed according to the instructions of the JC-1 assay kit (Beyotime, C2006, Wuhan, China). Image acquisition was carried out using a laser confocal microscope (Leica Stellaris 5, Leica Microsystems, Wetzlar Germany). For detection of JC-1 monomers, the excitation wavelength was set to 490 nm and the emission wavelength to 530 nm; for detection of JC-1 aggregates (J-aggregates), the excitation wavelength was set to 525 nm and the emission wavelength to 590 nm.

Intracellular Reactive Oxygen Species (ROS) Detection: Cell seeding and treatment were performed as described above. After the incubation period, fluorescent probe loading was carried out following the instructions of the CM-H_2_DCFDA ROS assay kit (Beyotime, S0035S, Wuhan China). Intracellular ROS levels were observed using a laser confocal microscope with the following detection parameters: excitation wavelength 495 nm, emission wavelength 530 nm.

Lipid Peroxidation Assay (BDPY 581/591 C11): Cell seeding and treatment were performed as described above. After treatment, cells were incubated with the BDPY 581/591 C11 probe (Beyotime, S0043S, Wuhan, China) according to the manufacturer’s instructions. The reduced form of the probe exhibits red fluorescence, whereas the oxidized form (after reaction with lipid hydroperoxides) shifts to green fluorescence. Fluorescence images were acquired using a laser confocal microscope (Leica Stellaris 5) with standard settings for red and green fluorescence. The degree of lipid peroxidation was evaluated by calculating the ratio of green to red fluorescence intensity.

### 2.6. Biochemical Indicators and Enzyme-Linked Immunosorbent Assay (ELISA)

HaCaT cells in the logarithmic growth phase were seeded into 6-well plates at a density of 2 × 10^5^ cells per well and cultured overnight until reaching approximately 80% confluence. Subsequently, the cells were co-incubated with H_2_O_2_ at a final concentration of 125 μM and Kuwanon A at a concentration of 25 μM for 24 h. For positive controls, 10 μM vitamin C (Vc) was used to assess antioxidant-related indicators, and 10 μM Ferrostatin-1 (Fer-1) was used to evaluate ferroptosis-related markers.

Total Protein Extraction and Quantification: HaCaT cells subjected to various treatment conditions were collected, and an appropriate amount of cell lysis buffer was added to extract total protein. After centrifugation, the supernatant was collected, and the protein concentration of each sample was determined strictly according to the instructions of the BCA protein assay kit to enable standardized calculation of subsequent indicators.

Detection of Oxidative Stress and Ferroptosis Markers: Equal amounts of protein samples were used to measure the activity or content of the following indicators according to the protocols of the respective assay kits (micro-assay and colorimetric methods): reduced glutathione (GSH), total superoxide dismutase (T-SOD), catalase (CAT), malondialdehyde (MDA), glutathione peroxidase (GPX), ferrous ion (Fe^2+^) content, and ferric ion (Fe^3+^) content.

ELISA Detection: The expression levels of the following proteins were determined strictly in accordance with the standard curve preparation and detection procedures described in the ELISA kit instructions: Inflammation-related factors: interleukin-6 (IL-6), tumor necrosis factor-α (TNF-α), interleukin-1β (IL-1β). Iron metabolism and ferroptosis-related markers: glutathione peroxidase 4 (GPX4), transferrin receptor 1 (TFR1), and ferritin heavy chain (FTH).

### 2.7. Proteomics

HaCaT cells were co-treated with H_2_O_2_ (125 μM) and 25 μM Kuwanon A for 24 h, with cells not treated with Kuwanon A serving as the blank control. Total protein was extracted using DB lysis buffer and quantified by the Bradford method. Aliquots of protein were digested with trypsin, desalted using a C18 column, and vacuum freeze-dried. The resulting peptides were reconstituted in 0.1% formic acid and analyzed using a Vanquish Neo UHPLC system coupled with an Orbitrap Astral mass spectrometer. Mass spectrometry was performed in data-independent acquisition (DIA) mode, with the resolution set to 240,000 for MS^1^ and 80,000 for MS^2^, using a precursor isolation window of 2 Th (300 windows). The raw data were searched and filtered using DIA-NN software version 1.8.1 (Q value < 0.01). Functional annotation, pathway enrichment, and protein–protein interaction network prediction of differentially expressed proteins were further analyzed using the InterProScan, KEGG, GO, and STRING databases.

### 2.8. Statistical Analysis

All studies were designed, where possible, to generate groups of equal size using randomization and blinded analysis. Data analysis followed the statistical methods reported in the literature. Data are presented as mean ± standard deviation and were statistically analyzed using one-way analysis of variance in GraphPadPrism.v10. All measurements were performed in at least triplicate unless stated otherwise. A *p*-value of <0.05 was considered statistically significant.

## 3. Results

### 3.1. Isolation and Identification of Chemical Constituents from the Root Bark of Morus alba L.

**Figure 1:**
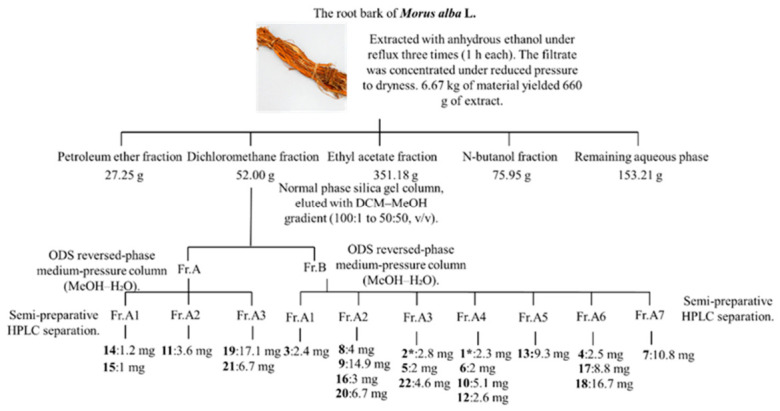
Flow chart of compound isolation.

**Figure 2:**
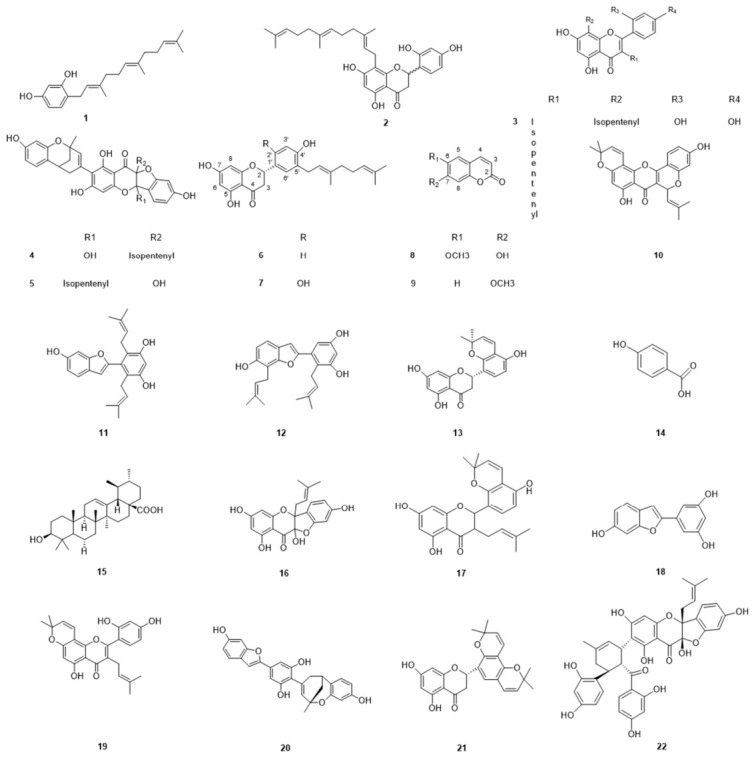
Chemical structures of compounds (with compounds **1** and **2** being new compounds).

The extraction and isolation process of compounds from the root bark of *Morus alba* L. is shown in Figure 1. Five fractions were obtained during the extraction phase. In order to quickly screen the protective effect of crude extract at the same dose, we conducted a rapid screening. Preliminary experiments indicated that the dichloromethane fraction exhibited the strongest protective effect against the hydrogen peroxide-induced HaCaT cell damage model (Figure S1). Therefore, the dichloromethane fraction was selected for further isolation, yielding a total of 22 monomeric compounds, whose chemical structures are presented in Figure 2. Among them, compounds **1** and **2** are new compounds.

**Figure 3:**
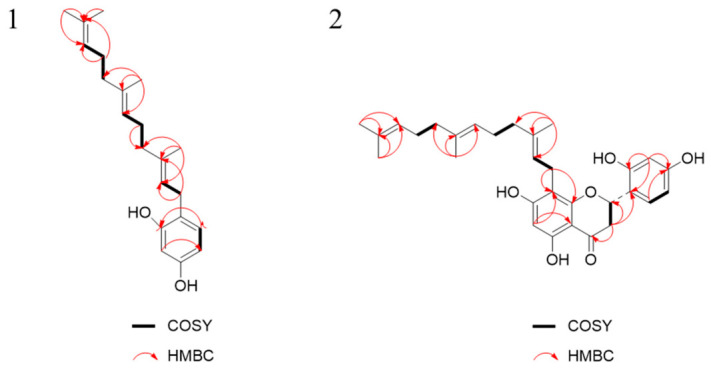
Key COSY correlations (bold black bonds) and HMBC correlations (red arrows) of compounds **1** and **2**.

**Compound 1** was obtained as a yellow oil. HR-ESI-MS showed a [M + Na]^+^ peak at *m*/*z* 337.21603, establishing its molecular weight as 314. Combined with the hydrogen spectrum, the molecular formula was determined to be C_21_H_30_O_2_. According to the ^1^H-NMR spectrum (Table 1), signals at δ 7.15 (d, *J* = 8.25 Hz, 1H), 6.34 (dd, *J* = 8.0, 2.0 Hz, 1H), and 6.22 (d, *J* = 2.15 Hz, 1H) were assigned to three aromatic protons on the benzene ring. Signals at δ 4.93 (1H, m), 4.97 (1H, m), and 5.13 (1H, t) were attributed to olefinic protons. The signals at δ 2.63 (dd, *J* = 6.45, 6.55 Hz, 1H) and 2.99 (dd, *J* = 9.3, 9.0 Hz, 1H) were assigned to the methylene group directly attached to the benzene ring. Signals at δ 1.56, 1.53, 1.48, and 1.45 (each 3H, s) corresponded to terminal methyl carbons. According to the ^13^C-NMR spectrum (Table 1), signals at δ 164.2 and 161.5 were attributed to aromatic carbons bearing –OH substituents. Signals at δ 121.7, 125.6, 109.8, and 99.6 were assigned to the aromatic ring carbons. Signals at δ 140.4, 118.7, 125.5, 136.0, 125.1, and 132.1 were assigned to olefinic carbons. Signals at δ 41.1, 40.9, 32.6, 27.8, 27.4, 25.9, 25.8, 17.8, 16.7, and 16.1 corresponded to methylene and methyl carbons, indicative of a long aliphatic chain. Based on the HSQC spectrum (Figure 3), the signal at δ 7.15 (1H, d, *J* = 8.25 Hz) was correlated with C-5 (δ 125.6) on the benzene ring; δ 6.34 (1H, dd, *J* = 8.0, 2.0 Hz) was correlated with C-6 (δ 109.8); δ 6.22 (1H, d, *J* = 2.15 Hz) was correlated with C-2 (δ 99.6); δ 5.13 (1H, t) was correlated with the olefinic carbon C-8 (δ 118.7); δ 4.93 (1H, m) was correlated with the olefinic carbon C-12; and δ 4.97 (1H, m) was correlated with the olefinic carbon C-16. According to the HMBC spectrum (Figure 3), the phenolic hydroxy groups were determined to be located at C-1 and C-3. The correlation of the side chain protons at δ 2.63 (1H, dd, *J* = 6.45, 6.55 Hz) and 2.99 (1H, dd, *J* = 9.3, 9.0 Hz) with C-4 (δ 121.7) confirmed the substitution position of the side chain. Based on H-H COSY correlations, the aromatic protons at δ 7.15, 6.22, and 6.34 showed coupling; the olefinic proton at δ 5.13 showed coupling with the methylene protons at δ 2.63 and 2.99; the olefinic proton at δ 4.91 showed coupling with the methylene protons at δ 1.67; and the methylene protons at δ 1.67 showed coupling with the methylene protons at δ 1.71. Integrating the ^1^H NMR, ^13^C NMR, HSQC, HMBC, H-H COSY, and HR-ESI-MS data (Figures S2–S8), the structure of this compound was identified as 4-Farnesylresorcinol.

**Compound 2** was obtained as a yellow oil. HR-ESI-MS showed an [M + H]^+^ peak at *m/z* 493.25909, establishing its molecular weight as 492. Combined with the hydrogen spectrum, the molecular formula was determined to be C_30_H_36_O_6_. According to the ^1^H-NMR spectrum (Table 2), signals at δ 7.17 (d, *J* = 2.0 Hz, 1H), 6.23 (d, *J* = 8.0, 2.0 Hz, 1H), and 5.83 (s, 1H) were assigned to H-6′ of ring B, H-2′ of ring B, and H-6 of ring A, respectively. The signal at δ 5.48 (dd, *J* = 12.0, 3.0 Hz, 1H) was assigned to H-2. The signals at δ 2.63 (m, 1H) and 2.86 (dd, *J* = 17.0, 12.0 Hz, 1H) were assigned to the two non-equivalent protons at C-3. The signals at δ 5.07 (1H, m) and 5.04 (2H, dtt, *J* ≈ 7.0 Hz) were collectively assigned to the CH protons H-2″, H-6″, and H-10″, corresponding to the internal double bonds of the three farnesyl isoprene units; appearing as multiplets with a total integral of 3H, this directly demonstrated that the side chain contains three double bonds. The signal at δ 4.51 (2H, s) was assigned to H-1″a/H-1″b, corresponding to the terminal =CH_2_ of the farnesyl group; appearing as a singlet without coupling, this is a characteristic feature of the terminal double bond of the farnesyl group. The signal at δ 3.07 (2H, dd, *J* ≈ 7.0 Hz) was assigned to CH_2_-1″. This methylene group is directly attached to position 6 of ring A and exhibits coupling with the adjacent olefinic proton, appearing as a dd peak. Its chemical shift is significantly higher than that of an ordinary alkyl methylene, confirming its direct attachment to the aromatic ring and serving as the key proton evidence for the farnesyl substitution at position C-8. The signals at δ 1.64, 1.55, 1.61, and 1.54 (each 3H, s) were assigned to the three gem-dimethyl groups C-12, C-13, C-14, and C-15, representing the most characteristic fingerprint region of the farnesyl group and directly indicating that the side chain is an all-trans farnesyl group (three isoprene units).

According to the ^13^C-NMR spectrum (Table 2), the signals at δ 198.86 (C=O, C-4), 75.9 (CH, C-2), and 43.28 (CH_2_, C-3) were attributed to the flavanone core. Signals at δ 166.22 (C-7), 163.37 (C-5), 96.48 (C-6), and 109.3 (C-8) were assigned to ring A of the flavanone core. Signals at δ 118.38 (C-1′), 156.64 (C-2′), 109.11 (C-3′), 159.55 (C-4′), 107.62 (C-5′), and 128.60 (C-6′) were assigned to ring B. Signals at δ 135.95, 162.16, and 132.02 were attributed to quaternary olefinic carbons; δ 125.71, 124.53, and 125.52 were assigned to olefinic methine carbons; δ 113.52 was assigned to the terminal olefinic CH_2_; signals at δ 40.83, 30.76, 27.82, 27.28, and 25.89 were assigned to methylene carbons; and signals at δ 22.42, 17.71, and 16.21 were assigned to methyl carbons.

Based on HMBC, HMQC, and H-H COSY spectra (Figure 3), CH_2_-1″ (δ 3.07) showed correlations with C-7 (δ 166.22), C-8 (δ 109.36), and C-9 (δ 162.35), confirming that the farnesyl side chain is attached to position C-8 of ring A. H-2 (δ 5.48) showed correlations with C-1′ (δ 118.38), C-2′ (δ 156.64), C-6′ (δ 128.60), and C-5′ (δ 107.62), confirming that ring B is attached to position C-2. Integrating the ^1^H NMR, ^13^C NMR, HSQC, HMBC, H-H COSY, and HR-ESI-MS data (Figures S10–S16), the structure of this compound was determined and named 8-Farnesyl-2′,4′,5,7-tetrahydroxyflavanone.

The remaining known compounds were identified by comparison with spectral data from the literature as follows: 3: Kuwanon C [16]; 4: Sanggenon B [17]; 6: Kuwanon S [18]; 7: Kuwanon E [19]; 8: Scopoletin [20]; 9: 7-Methoxycoumarin [21]; 10: Cyclomorusin [22]; 11: Mulberrofuran V [23]; 12: Cathafuran B [16]; 13: Sanggenon H [24]; 14: p-Hydroxybenzoic acid [25]; 15: Ursolic acid [26]; 16: Nigragenon E [27]; 17: Kuwanon A [28]; 18: Moracin M [29]; 19: Morusin [30]; 20: Mulberrofuran H [31]; 21: Sanggenol O [32]; 22: Sanggenon C [33]. No relevant spectral information has been reported for compound **5**. Detailed information is provided in the Supplementary Materials.

### 3.2. Kuwanon A Is an Active Compound with Antioxidant and Anti-Damage Properties Derived from the Root Bark of Morus alba L.

**Figure 4:**
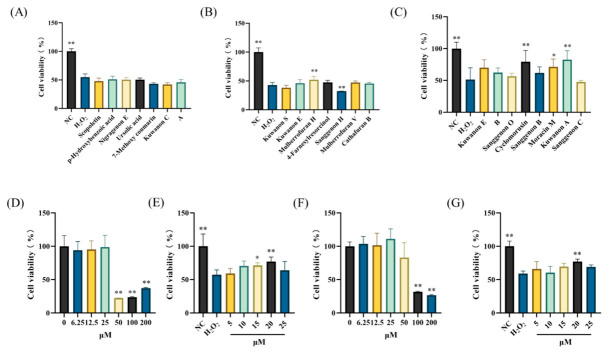
Activity screening using the H_2_O_2_-induced HaCaT cell damage model. (**A**–**C**) Preliminary screening results, where (**A**) represents compound **18**, and (**B**) represents 8-Farnesyl-2′,4′,5,7-tetrahydroxyflavanone. (**D**) Effect of Kuwanon A on the viability of normal HaCaT cells. (**E**) Effect of Kuwanon A on the viability of H_2_O_2_-damaged HaCaT cells. (**F**) Effect of cyclomorusin on the viability of normal HaCaT cells. (**G**) Effect of cyclomorusin on the viability of H_2_O_2_-damaged HaCaT cells. n = 6: In each experiment, six replicate wells were set up as repeat groups. (**A**–**C**,**E**,**G**): * *p* < 0.05, ** *p* < 0.01 vs. H_2_O_2_ group, (**D**,**F**): * *p* < 0.05, ** *p* < 0.01 vs. control group.

The active compounds isolated from the root bark of Morus alba were screened using HaCaT cells damaged by H_2_O_2_, and the results are shown in Figure 4A–C. Among them, Mulberrofuran H, Cyclomorusin, Moracin M, and Kuwanon A exhibited good activity. Considering the large isolation yield and favorable activity of Kuwanon A, Kuwanon A and Cyclomorusin were selected for subsequent experiments. Figure 4D illustrates the effect of Kuwanon A on the viability of normal HaCaT cells. Within the concentration range of 6.25 to 25 μM, Kuwanon A showed no significant toxicity *(p* > 0.05), whereas at concentrations ranging from 50 to 200 μM, Kuwanon A exhibited significant activity on HaCaT cells (*p* < 0.01). Concentrations ranging from 5 to 25 μM were selected to further investigate the efficacy and concentration range of Kuwanon A in H_2_O_2_-damaged HaCaT cells (Figure 4E). It was found that Kuwanon A exerted a certain protective effect on HaCaT cells within the concentration range of 10 to 20 μM, with 15 μM and 20 μM significantly enhancing cell viability compared with the model group (*p* < 0.05, *p* < 0.01). Figure 4F shows the effect of Cyclomorusin on the toxicity of normal HaCaT cells. Within the concentration range of 6.25 to 50 μM, no significant toxicity was observed (*p* > 0.05), whereas at concentrations ranging from 100 to 200 μM, Cyclomorusin exhibited significant activity on HaCaT cells (*p* < 0.01). In the H_2_O_2_-damaged HaCaT cell model, a significant protective effect was observed only at a concentration of 20 μM (Figure 4G) (*p* < 0.01). Considering the isolation yield and the requirements for subsequent experiments, Kuwanon A exhibits a more robust dose–response relationship and a wider therapeutic window, making it a more suitable candidate compound for mechanistic studies.

### 3.3. Kuwanon A Ameliorates Oxidative Stress and Mitochondrial Damage in Hydrogen Peroxide-Damaged HaCaT Cells

**Figure 5:**
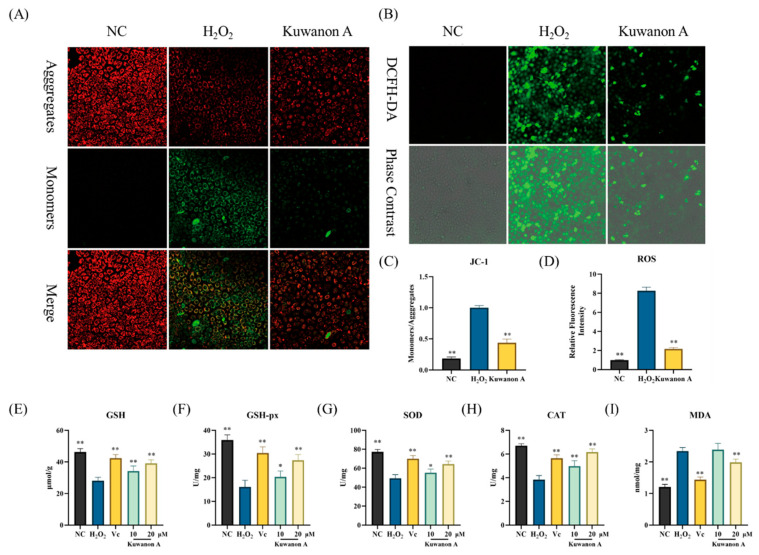
Kuwanon A ameliorates H_2_O_2_-induced oxidative stress levels in HaCaT cells. (**A**) Mitochondrial membrane potential (JC-1) staining images of HaCaT cells; (**B**) ROS staining images of HaCaT cells; (**C**,**D**) Quantitative fluorescence statistical analysis; (**E**–**I**) The contents of GSH, GSH-Px, SOD, CAT, and MDA in HaCaT cells, respectively. n = 6: In each experiment, six biological replicates were set up, * *p* < 0.05, ** *p* < 0.01 vs. H_2_O_2_ group.

In further experiments, the effect of Kuwanon A on the mitochondrial membrane potential of H_2_O_2_-damaged HaCaT cells was assessed using JC-1 staining (Figure 5A). It was observed that after H_2_O_2_ treatment, compared with the normal group, red fluorescence (aggregates) decreased while green fluorescence (monomers) increased. The fluorescence intensity was quantified using ImageJ 1.8.0 software, revealing that the monomers/aggregates ratio in the model group was significantly higher than that in the normal group (*p* < 0.01). Treatment with 20 μM Kuwanon A significantly ameliorated this change, reducing the monomers/aggregates fluorescence ratio (*p* < 0.01). Additionally, reactive oxygen species (ROS) levels were measured using the DCFH-DA probe (Figure 5B). Compared with the normal group, H_2_O_2_-damaged HaCaT cells exhibited substantial green fluorescence, indicating a marked increase in ROS production. Quantification of fluorescence intensity using ImageJ showed that the fluorescence intensity in the model group was significantly higher than that in the normal group (*p* < 0.01), while treatment with 20 μM Kuwanon A significantly alleviated this effect, reducing the fluorescence intensity (*p* < 0.01). Furthermore, commercial kits were used to assess the levels of GSH, GSH-PX, SOD, CAT, and MDA (Figure 5E–I). Compared with the normal group, H_2_O_2_-damaged HaCaT cells exhibited significantly decreased levels of GSH, GSH-PX, SOD, and CAT (*p* < 0.01), along with a significant increase in MDA (*p* < 0.01). Intervention with 10 μM and 20 μM Kuwanon A resulted in varying degrees of improvement, with the higher concentration of Kuwanon A showing the most pronounced effect (*p* < 0.01).

### 3.4. Kuwanon A Ameliorates Ferroptosis and Inflammatory Response in H_2_O_2_-Damaged HaCaT Cells

**Figure 6:**
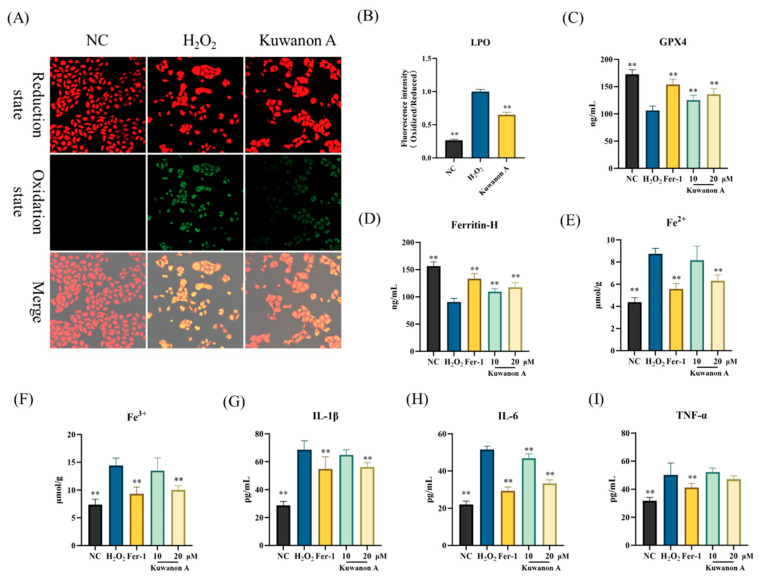
Kuwanon A ameliorates H_2_O_2_-induced ferroptosis and inflammatory response in HaCaT cells. (**A**) Lipid peroxidation staining images; (**B**) Quantitative fluorescence analysis of lipid peroxidation levels; (**C**,**D**) Protein expression levels of GPX4 and Ferritin-H in HaCaT cells; (**E**,**F**) Intracellular Fe^2+^ and Fe^3+^ levels; (**G**–**I**) Protein expression levels of IL-1β, IL-6, and TNF-α in cells. n = 6: In each experiment, six biological replicates were set up, ** *p* < 0.01 vs. H_2_O_2_ group.

Lipid peroxidation fluorescent staining (Figure 6A) revealed that in H_2_O_2_-damaged HaCaT cells, red fluorescence (reduction state) decreased, while green fluorescence (oxidation state) intensity increased. Quantification showed that the reduction state/oxidation state ratio in the model group was significantly higher than that in the normal group (*p* < 0.01) (Figure 6B). Following Kuwanon A intervention, this ratio was significantly decreased compared with the model group (*p* < 0.01). ELISA and biochemical kit assays indicated that in H_2_O_2_-damaged HaCaT cells, GPX4 and Ferritin-H levels were significantly decreased, while Fe^2+^ and Fe^3+^ levels were significantly increased. Intervention with 10 μM and 20 μM Kuwanon A resulted in improvements, with the 20 μM concentration showing the most significant effect (*p* < 0.01) (Figure 6C–F), comparable to the positive control group treated with Fer-1. Additionally, inflammatory factors were measured using ELISA kits (Figure 6G–I). H_2_O_2_-damaged HaCaT cells also exhibited an inflammatory response, characterized by significant increases in IL-1β, IL-6, and TNF-α. Kuwanon A at different concentrations ameliorated the inflammatory response, significantly reducing IL-1β and IL-6 levels compared with the model group (*p* < 0.01).

### 3.5. Proteomics Reveals the Effects of Kuwanon A on H_2_O_2_-Damaged HaCaT Cells

**Figure 7:**
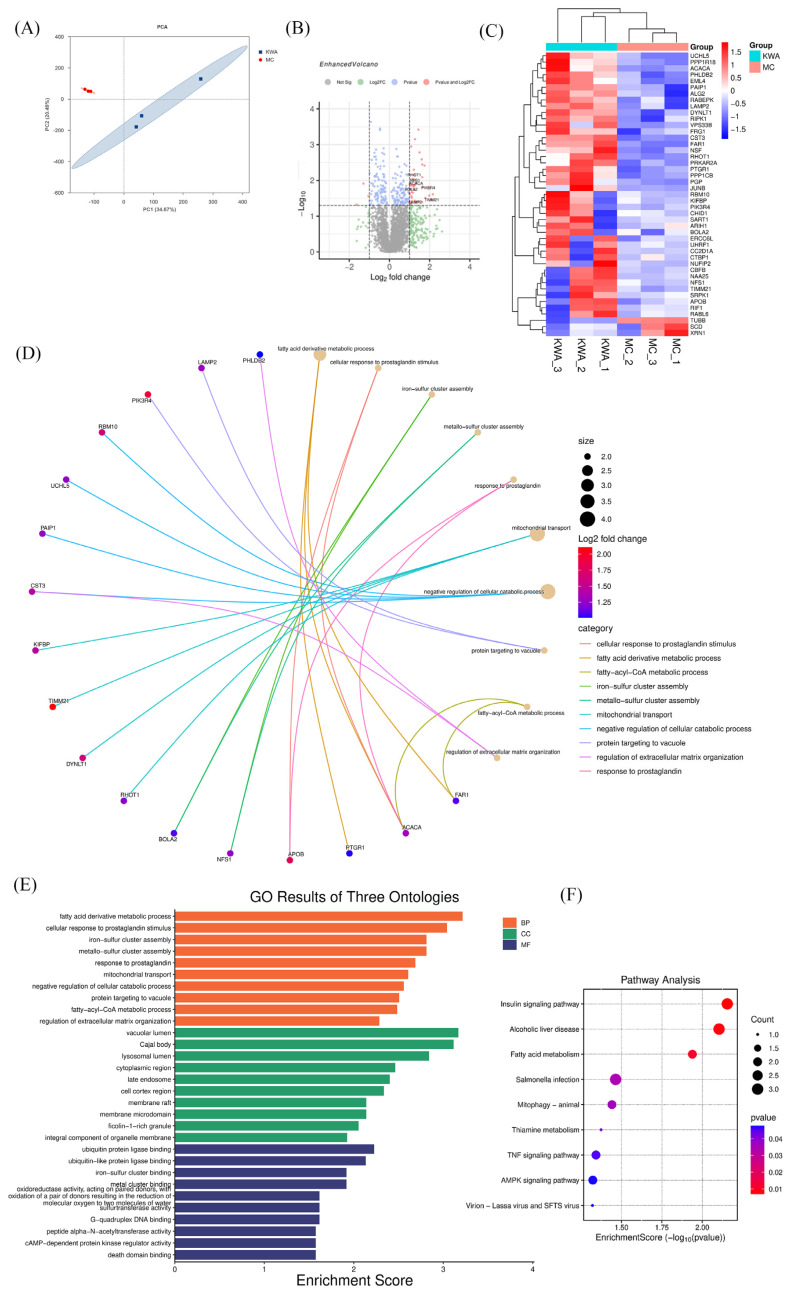
Kuwanon A upregulates NFS1 to regulate the iron–sulfur cluster pathway and ameliorate ferroptosis. (**A**) PCA plot of sample distribution; (**B**) Volcano plot of differentially expressed proteins; (**C**) Cluster analysis heatmap of differentially expressed proteins; (**D**,**E**) GO enrichment analysis of differentially expressed proteins; (**F**) KEGG enrichment analysis of differentially expressed proteins. n = 3: Each experiment was performed with three biological replicates.

To investigate the mechanism of action of Kuwanon A, proteomics experiments were performed. PCA analysis in Figure 7A showed good separation between the two groups. The volcano plot in Figure 7B displays 45 differentially expressed proteins, including 3 downregulated proteins (TUBB, SCD, XRN1) and 42 upregulated proteins (including NFS1, ACACA, RHOT1, BOLA2, PIK3R4, AMP2, etc.) compared with the model group. Cluster analysis was performed for each differential protein and is presented in Figure 7C. GO enrichment analysis (Figure 7D,E) revealed that the differentially expressed proteins were mainly enriched in the pathways of iron–sulfur cluster assembly and metallo–sulfur cluster assembly (BP), as well as iron–sulfur cluster binding, metal cluster binding, and oxidoreductase activity (MF). KEGG analysis (Figure 7F) indicated that the differentially expressed proteins were primarily enriched in inflammation (TNF and AMPK) and mitophagy. Based on the proteomics results, it is inferred that Kuwanon A is associated with iron–sulfur clusters and the NFS1 protein.

## 4. Discussion

The extract from the root bark of *Morus alba* L. has been reported to possess strong antioxidant activity, and both its extracts and isolated components have been used in the fields of skin whitening and anti-aging. Obtaining natural skincare ingredients from *Morus alba* L. represents a promising strategy. From the dichloromethane-soluble active fraction of the root bark of Morus alba L., we isolated 22 compounds, including Diels–Alder type adducts (**4**, **5**, **13**, **22**), flavonoids (**2**, **3**, **6**, **7**, **10**, **16**, **17**, **19**, **21**), benzofurans (**11**, **12**, **18**, **20**), coumarins (**8**, **9**), and others (**1**, **14**, **15**). Among these, two new compounds (**1**, **2**) were discovered, and compounds (**12**, **16**) were isolated from the root bark of *Morus alba* L. for the first time.

The skin is highly sensitive to external stimuli. Keratinocytes, the main components of the epidermis, serve as the primary skin barrier and are highly susceptible to oxidative damage, which can lead to severe skin-related diseases [1]. Accumulating evidence indicates that when epidermal cells (keratinocytes) are continuously exposed to oxidative stress, excessive accumulation of ROS causes oxidative modification of nucleic acids, lipids, proteins, and other small intracellular molecules, ultimately leading to cell death, exacerbating skin pigmentation and aging [34]. This results in changes in skin tone uniformity, wrinkles, sagging, dryness, and roughness [2]. A robust oxidative defense system is crucial for protecting against and treating diseases associated with oxidative stress [35].

H_2_O_2_-induced oxidative stress or cell death in HaCaT keratinocytes has been widely used as an in vitro screening model for skin oxidative stress [36]. In this study, we used an H_2_O_2_-induced HaCaT cell damage model to perform preliminary activity screening of the isolated compounds. Kuwanon A exhibited excellent cytoprotective effects and was selected as the preferred compound for further investigation.

Mitochondria are a major source of ROS. During normal respiratory chain metabolism, mitochondria produce superoxide anions (O_2_^−^•) as a byproduct, which is the most direct form of mitochondrial ROS [37]. The generated ROS can trigger the opening of the mitochondrial permeability transition pore (mPTP). The opening of mPTP further stimulates substantial ROS generation, forming a vicious cycle known as “ROS-induced ROS release” (RIRR) [38]. Fluorescence probe analysis and confocal microscopy observations indicated that Kuwanon A reduces intracellular ROS levels after damage and improves mitochondrial function.

In the oxidative stress system, SOD acts as the first line of defense, dismutating superoxide anions into hydrogen peroxide [39]. Subsequently, CAT and GSH-PX are responsible for clearing high and low concentrations of hydrogen peroxide and lipid peroxides, respectively. GSH-PX uses reduced GSH as a substrate and is particularly important for maintaining cell membrane stability [40]. GSH itself not only provides thiol reducing power and directly neutralizes free radicals but also reflects the cellular redox balance through the GSH/GSSG ratio. These antioxidant enzymes and antioxidants together constitute the defense barrier, while MDA is an end product of lipid peroxidation, and its level directly reflects the extent of cell membrane damage caused by oxidation [41]. Combined detection of SOD, CAT, GSH-PX, GSH, and MDA allows for comprehensive evaluation of the body’s antioxidant capacity and oxidative damage status. The results showed that Kuwanon A ameliorated the abnormalities in SOD, CAT, GSH-PX, GSH, and MDA levels induced by H_2_O_2_ in HaCaT cells. In summary, Kuwanon A is a potent antioxidant.

Oxidative stress is the core driver of ferroptosis, a regulated form of cell death characterized by iron-dependent accumulation of lipid peroxidation [42]. Labile iron catalyzes the generation of highly reactive hydroxyl radicals (•OH) through the Fenton reaction, directly initiating lipid peroxidation chain reactions [43]. Among the antioxidant indicators, Kuwanon A reduced the H_2_O_2_-induced increase in MDA, suggesting that H_2_O_2_ might induce ferroptosis in HaCaT cells. Fluorescence probe and confocal microscopy observations confirmed that H_2_O_2_ indeed caused lipid peroxidation accumulation in HaCaT cells, which was mitigated by Kuwanon A. Ferroptosis is fundamentally characterized by the lethal accumulation of lipid peroxides and iron homeostasis imbalance. When the intracellular antioxidant system, particularly the GSH-dependent GPX4, fails to clear excess lipid peroxides, oxidative stress sharply increases, triggering ferroptosis [44]. Detection of GPX4 revealed that Kuwanon A increased GPX4 levels in HaCaT cells. These results indicate that oxidative stress induced by H_2_O_2_ leads to ferroptosis in HaCaT cells, and Kuwanon A serves as an effective intervention. Furthermore, Ferritin heavy chain (FTH1) is a key intracellular protein that inhibits ferroptosis. FTH1 possesses ferroxidase activity, converting pro-oxidant Fe^2+^ into Fe^3+^, thereby preventing Fe^2+^ from participating in the Fenton reaction and safely sequestering Fe^3+^ within its protein cavity, storing iron in a non-toxic form and preventing the expansion of the labile iron pool [45]. Experiments showed that H_2_O_2_ increased intracellular Fe^2+^ and Fe^3+^ levels, while Kuwanon A reduced labile iron content and increased FTH1 expression in HaCaT cells. In summary, Kuwanon A can protect against H_2_O_2_-induced ferroptosis in HaCaT cells by reducing lipid peroxidation and ameliorating iron homeostasis imbalance. Additionally, Kuwanon A possesses certain anti-inflammatory effects. Hydrogen peroxide treatment significantly upregulated the expression of pro-inflammatory cytokines (IL-1β, TNF-α, and IL-6) in HaCaT cells, while intervention with Kuwanon A markedly reduced the secretion levels of these cytokines, indicating that Kuwanon A possesses obvious anti-inflammatory activity. IL-1β, TNF-α, and IL-6 are key cytokines involved in the inflammatory response, and their overexpression can aggravate cellular injury, amplify oxidative stress, and further induce cellular dysfunction. Kuwanon A alleviates inflammatory damage by suppressing the release of these pro-inflammatory factors, which acts synergistically with its antioxidant and anti-ferroptosis effects to protect HaCaT cells against H_2_O_2_-induced oxidative injury. These findings further enrich the underlying mechanism of Kuwanon A, suggesting that it protects cells not only by inhibiting ferroptosis but also by reducing inflammatory cytokine release and mitigating inflammatory responses, thereby maintaining cellular homeostasis in a multi-dimensional manner.

Oxidative stress represents an upstream pathological state characterized by excessive accumulation of intracellular ROS and disruption of redox homeostasis, which can trigger cellular injury and initiate multiple forms of programmed cell death [46]. Ferroptosis is an iron-dependent type of programmed cell death driven by excessive lipid peroxidation, mainly regulated by the SLC7A11/GPX4 signaling axis [47]. It is caspase-independent and distinctly different from classical apoptosis and general oxidative stress injury in both molecular mechanisms and cellular morphology. In this study, Kuwanon A not only eliminated excessive intracellular ROS and alleviated global oxidative stress, but also modulated key ferroptosis-related molecules and suppressed the accumulation of lipid peroxidation, thereby blocking the progression of ferroptosis. These findings indicate that the skin-protective effect of Kuwanon A is not merely attributed to general antioxidant activity, but involves dual regulation of redox homeostasis and specific inhibition of ferroptosis.

To further explore the underlying mechanism of Kuwanon A, quantitative proteomics technology was employed to detect protein-level changes in H_2_O_2_-damaged HaCaT cells following Kuwanon A intervention. The results indicated that Kuwanon A upregulated the expression of proteins such as NFS1, ACACA, RHOT1, and BOLA2, with NFS1 showing the most significant change. GO enrichment analysis of differentially expressed proteins revealed significant enrichment in pathways related to “iron–sulfur cluster assembly” and “metallo–sulfur cluster assembly” (Biological Process), as well as “iron–sulfur cluster binding,” “metal cluster binding,” and “oxidoreductase activity” (Molecular Function).

Cysteine desulfurase (NFS1) is a key enzyme in the biosynthesis of mitochondrial iron–sulfur (Fe-S) clusters [48]. By maintaining Fe-S cluster homeostasis and regulating the ACO1/IRP1 axis, the SLC7A11/GPX4 axis, and the p53/STAT signaling pathways, NFS1 participates in the regulation of ferroptosis [49]. When the NFS1 function is impaired, intracellular labile iron levels rise, leading to ROS accumulation and subsequent lipid peroxidation. In recent years, researchers have progressively identified NFS1 as an upstream node in the ferroptosis regulatory network, revealing its multidimensional role in determining cell fate by integrating iron homeostasis, oxidative stress, and lipid metabolism [50]. NFS1 deficiency or dysfunction leads to impaired Fe-S cluster synthesis, followed by the conversion of ACO1 to IRP1. This results in upregulation of transferrin receptor 1 (TfR1) and downregulation of FTH1, elevating labile iron levels and activating an iron starvation response. Additionally, NFS1 downregulation activates the p53 pathway, inhibits the transcriptional activity of SLC7A11, impairs the function of the Xc^−^ system, and restricts cysteine uptake and GSH synthesis [48]. Ultimately, GPX4 activity is reduced, and lipid peroxidation accumulation is enhanced. Concurrently, antioxidant enzymes such as GPX4 are inhibited, leading to excessive ROS accumulation, eventually triggering ferroptosis. NFS1 expression varies significantly across different tissues. Upregulation of NFS1 in tumors can enhance tumor cell resistance to ferroptosis, thereby promoting tumor growth, drug resistance, and metastatic ability [51]. Conversely, downregulation of NFS1 in cardiomyocytes and neurons exacerbates ferroptosis and leads to dysfunction [52]. The proteomics results suggest that Kuwanon A upregulates NFS1 to regulate the iron–sulfur cluster assembly pathway, thereby protecting HaCaT cells from ferroptosis induced by H_2_O_2_ damage. It should be noted that in the proteomic study of this research, each group had 3 biological replicates, which has limitations in evaluating true biological variability and statistical power. In addition, the enrichment analysis based on unbalanced differential proteins may lead to biases in interpretation. In the future, it is necessary to expand the sample size to further verify the robustness of the conclusions.

## 5. Conclusions

This study began with the dichloromethane active fraction of Morus alba L. root bark, from which 22 compounds were isolated. Among them, Kuwanon A was selected as the preferred compound due to its excellent cytoprotective effects. Using an H_2_O_2_-induced oxidative stress model in HaCaT keratinocytes, this study confirmed the potential of Kuwanon A as a natural antioxidant for skin protection. Furthermore, it was deduced that Kuwanon A acts through NFS1-mediated regulation of iron–sulfur cluster homeostasis, providing a novel molecular target and lead compound for intervening in oxidative stress-related skin damage by targeting ferroptosis. Furthermore, before extrapolating the conclusions to clinical applications, further validation is required using more physiologically relevant models (e.g., primary cells, animal models, or human skin equivalents).

## Figures and Tables

**Table 1:** ^1^H (500 MHz) and ^13^C (125 MHz) NMR spectral data of compound **1**.

No.	^1^H-NMR (500 MHz, MEOD)	^13^C-NMR (125 MHz, MEOD)
	1	1
1		164.2
2	6.22 (1H, d, *J* = 2.15 Hz)	99.6
3		161.5
4		121.7
5	7.15 (1H, d, *J* = 8.25 Hz)	125.6
6	6.34 (1H, dd, *J* = 8.0, 2.0 Hz)	109.8
7	2.63 (1H, dd, *J* = 6.45, 6.55 Hz) 2.99 (1H, dd, *J* = 9.3, 9.0 Hz)	32.6
8	5.13 (1H, t)	118.7
9		140.4
10	1.71 (2H, m)	41.4
11	1.67 (2H, m)	27.4
12	4.93 (1H, m)	125.5
13		136.0
14	1.84 (2H, t)	40.8
15	1.94 (2H, m)	27.8
16	4.97 (1H, m)	125.1
17		132.1
18	1.56 (3H, s)	25.9
19	1.48 (3H, s)	17.8
20	1.45 (3H, s)	16.1
21	1.53 (3H, s)	16.7

**Table 2:** ^1^H (500 MHz) and ^13^C (125 MHz) NMR spectral data of compound **2**.

No.1	^1^H-NMR (500 MHz, MEOD)	^13^C-NMR (125 MHz, MEOD)
	2	2
1		
2	5.48 (1H, dd, *J* = 12.0, 3.0 Hz)	75.9
3	2.63 (1H, m), 2.86 (1H, dd, 17.0, 12.0 Hz)	43.28
4		198.86
5		163.37
6	5.83 (1H, s)	96.48
7		166.22
8		109.3
9		162.35
10		103.32
1′		118.38
2′		156.64
3′	6.23 (1H, d, *J* = 2.0 Hz)	103.36
4′		159.55
5′	6.23 (1H, d, *J* = 2.0 Hz)	107.62
6′	7.17 (1H, d, *J* = 2.0 Hz)	128.60
1″	4.51 (2H, s)	22.42
2″	5.04 (2H, dtt)	125.71
3″		135.95
4″	1.94 (2H, dt)	41.04
5″	1.88 (2H, dd, *J* = 5.5, 7.0 Hz)	27.82
6″	5.04 (2H, ddt)	124.53
7″		162.16
8″	1.94 (2H, dt)	43.28
9″	1.94 (2H, dt)	27.28
10″	5.07 (1H, m)	125.52
11		132.02
12	1.64 (3H, s)	26.1
13	1.55 (3H, s)	17.71
14	1.61 (3H, s)	16.21
15	1.54 (3H, s)	16.18

## Data Availability

The original contributions presented in this study are included in the article/Supplementary Material. Further inquiries can be directed to the corresponding authors.

## Supplementary Materials

The following supporting information can be downloaded at: https://www.mdpi.com/article/10.3390/antiox15060657/s1.
