# Supplementary material for: Kuwanon A from Morus alba L. Alleviates H2O2-Induced Oxidative Damage in HaCaT Keratinocytes by Inhibiting Ferroptosis and Enhancing Antioxidant Capacity
Source: Antioxidants (Basel). 2026 May 22;15(6):657. doi: 10.3390/antiox15060657 (PMC13296179; doi:10.3390/antiox15060657)
Supplement: Supplementary file 1 [file antioxidants-15-00657-s001.zip › antioxidants-4253947-supplementary.pdf]

Kuwanon A from Morus alba L. Alleviates H_2O_2-Induced Oxidative Damage in HaCaT Keratinocytes by Inhibiting Ferroptosis and Enhancing Antioxidant Capacity

Yan Liu[a1], Hening Fu[a1], Junjie Ma[a1], Youqing Wang[c], Zhaohua Shi[b], Yupeng Liu[a], Xianju Huang[a], Bingchen Han[ac]*, Jun Li[a]*

a School of Pharmaceutical Science, South-Central Minzu University, Wuhan 430074, Hubei, China

b Hubei Shi-Zhen Laboratory, Wuhan, Hubei, 430065, China

c College of life Sciences, South-Central Minzu University, Wuhan 430074, Hubei, China

contents

Figure S1: (A) Screening of cell modeling concentration; (B) Screening of active fractions. 1: Petroleum ether fraction; 2: Dichloromethane fraction; 3: Ethyl acetate fraction; 4: n-Butanol fraction; 5: Residual aqueous phase. 3
Figure S2: HR-ESI-MS of compound **1** 3
Figure S3: ^{1}H-NMR (500 MHz, MEOD) spectrum of compound **1** 4
Figure S4: 13C-NMR (151 MHz, MEOD) spectrum of compound **1** 4
Figure S5: 13C-NMR-DEPT spectrum of compound **1** 5
Figure S6: HSQC spectrum of compound **1** 6
Figure S7: HMBC spectrum of compound **1** 6
Figure S8: 1H-1H COSY spectrum of compound **1** 7
Figure S9: UV spectrum of compound **1** 7
Figure S10: HR-ESI-MS of compound **2** 8
Figure S11: 1H-NMR (500 MHz, MEOD) spectrum of compound **2** 8
Figure S12: 13C-NMR (126 MHz, MEOD) spectrum of compound **2** 9
Figure S13: 13C-NMR-DEPT spectrum of compound **2** 10
Figure S14: HSQC spectrum of compound **2** 11
Figure S15: HMBC spectrum of compound **2** 11
Figure S16: 1H-1H COSY spectrum of compound **2** 12
Figure S17: UV spectrum of compound **2** 13
Figure S18: 1H-NMR (500 MHz, MEOD) spectrum of compound 3 14
Figure S19: 13C-NMR (126 MHz, MEOD) spectrum of compound **3** 14
Figure S20: 1H-NMR (500 MHz, CD3OD) spectrum of compound **4** 15

---

1 Co-first authors. These authors provided the same contribution to this work.
Yan Liu: 2023110457@mail.scuec.edu.cn
Hening Fu: 2023110427@mail.scuec.edu.cn
* Co-corresponding author
Bingchen Han: 2023010071@mail.scuec.edu.cn
Jun Li: lijun-pharm@hotmail.com

Figure S21: 13C-NMR (126 MHz, CD_3OD) spectrum of compound **4**..16
Figure S22: 1H-NMR (500 MHz, MEOD) spectrum of compound **5** 17
Figure S23: 1H-NMR (500 MHz, MEOD) spectrum of compound **5** 18
Figure S24: 1H-NMR (500 MHz, MEOD) spectrum of compound **6** 19
Figure S25: 13C-NMR (126 MHz, MEOD) spectrum of compound **6**...19
Figure S26: 1H-NMR (500 MHz, MEOD) spectrum of compound **7** 20
Figure S27: 13C-NMR (126 MHz, MEOD) spectrum of compound **7**...21
Figure S28: 1H-NMR (500 MHz, MEOD) spectrum of compound **8** 22
Figure S29: 13C-NMR (126 MHz, MEOD) spectrum of compound **8**...22
Figure S30: 1H-NMR (500 MHz, MEOD) spectrum of compound **9** 23
Figure S31: 13C-NMR (151 MHz, MEOD) spectrum of compound **9**...24
Figure S32: 1H-NMR (500 MHz, MEOD) spectrum of compound **10** 25
Figure S33: 13C-NMR (126 MHz, MEOD) spectrum of compound **10**...25
Figure S34: 1H-NMR (500 MHz, MEOD) spectrum of compound **11**..26
Figure S35: 13C-NMR (126 MHz, MEOD) spectrum of compound **11**...27
Figure S36: 1H-NMR (500 MHz, MEOD) spectrum of compound **12** 28
Figure S37: 13C-NMR (126 MHz, MEOD) spectrum of compound **12**...28
Figure S38: 1H-NMR (600 MHz, MEOD) spectrum of compound **13** 29
Figure S39: 13C-NMR (151 MHz, MEOD) spectrum of compound **13**...30
Figure S40: 1H-NMR (600 MHz, MEOD) spectrum of compound **14** 31
Figure S41: 13C-NMR (151 MHz, MEOD) spectrum of compound **14**...31
Figure S42: 1H-NMR (600 MHz, MEOD) spectrum of compound **15** 32
Figure S43: 13C-NMR (151 MHz, MEOD) spectrum of compound **15**...33
Figure S44: 1H-NMR (500 MHz, MEOD) spectrum of compound **16** 34
Figure S45: 13C-NMR (126 MHz, MEOD) spectrum of compound **16**...34
Figure S46: 1H-NMR (500 MHz, MEOD) spectrum of compound **17** 35
Figure S47: 13C-NMR (126 MHz, MEOD) spectrum of compound **17**...36
Figure S48: 1H-NMR (500 MHz, MEOD) spectrum of compound **18** 37
Figure S49: 13C-NMR (126 MHz, MEOD) spectrum of compound **18**...37
Figure S50: 1H-NMR (600 MHz, MEOD) spectrum of compound **19** 38
Figure S51: 13C-NMR (151 MHz, MEOD) spectrum of compound **19**...39
Figure S52: 1H-NMR (500 MHz, MEOD) spectrum of compound **20** 40
Figure S53: 13C-NMR (126 MHz, MEOD) spectrum of compound **20**...40
Figure S54: 1H-NMR (500 MHz, MEOD) spectrum of compound **21** 41
Figure S55: 13C-NMR (126 MHz, MEOD) spectrum of compound **21**...42
Figure S56: 13C-NMR (126 MHz, MEOD) spectrum of compound **22**...43
Figure S57: 13C-NMR (126 MHz, MEOD) spectrum of compound **22**...43

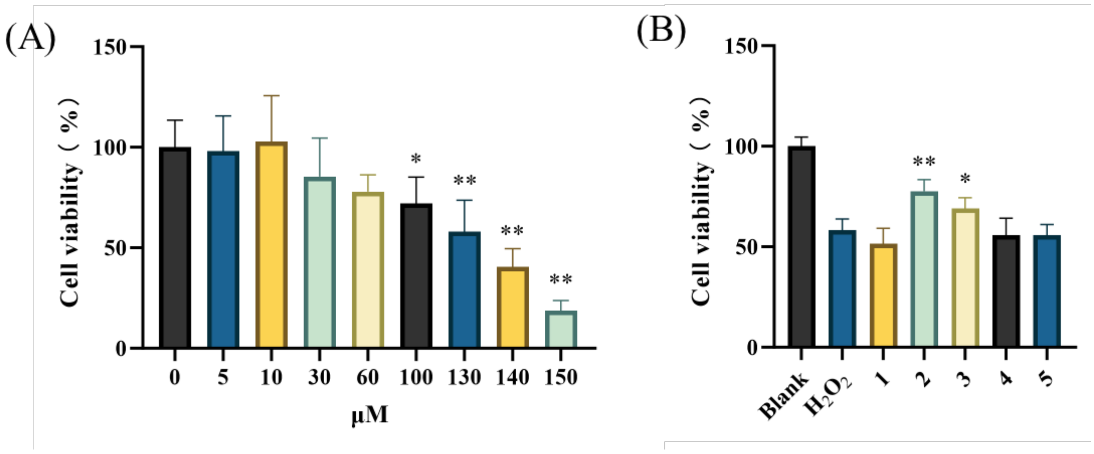

Figure S1: (A) Screening of cell modeling concentration; (B) Screening of active fractions. 1: Petroleum ether fraction; 2: Dichloromethane fraction; 3: Ethyl acetate fraction; 4: n-Butanol fraction; 5: Residual aqueous phase. Take the lyophilized powder of each extract at 100 μg/mL and perform preliminary screening according to the method in section 2.4.

n=6, **P* < 0.05, ***P* < 0.01 vs. control group.

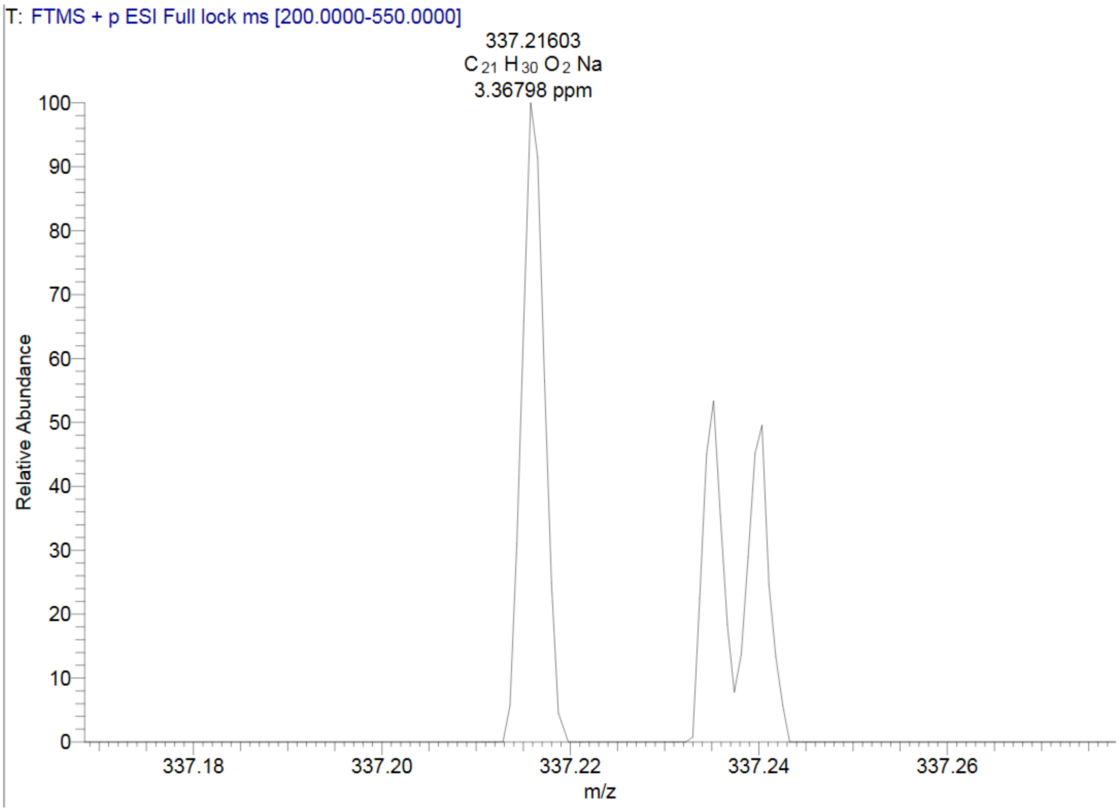

Figure S2: HR-ESI-MS of compound **1**

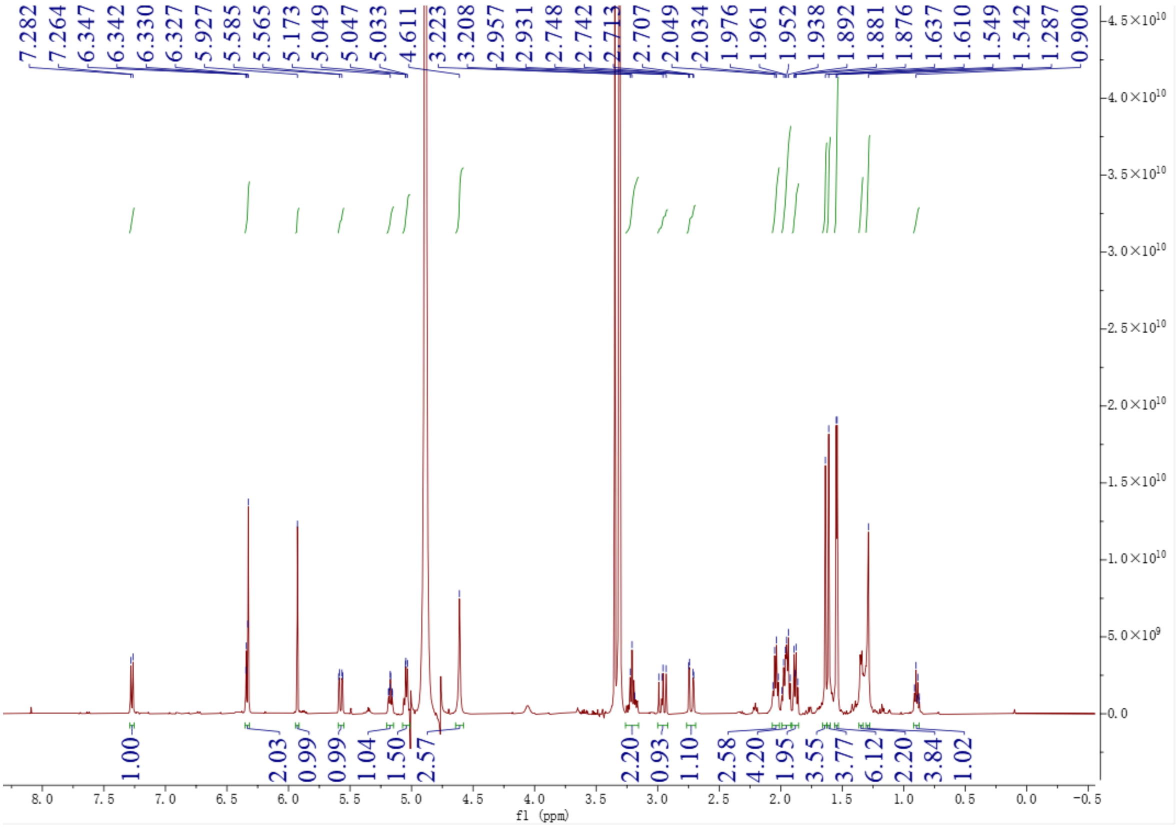

Figure S3: ^{1}H-NMR (500 MHz, MEOD) spectrum of compound **1**

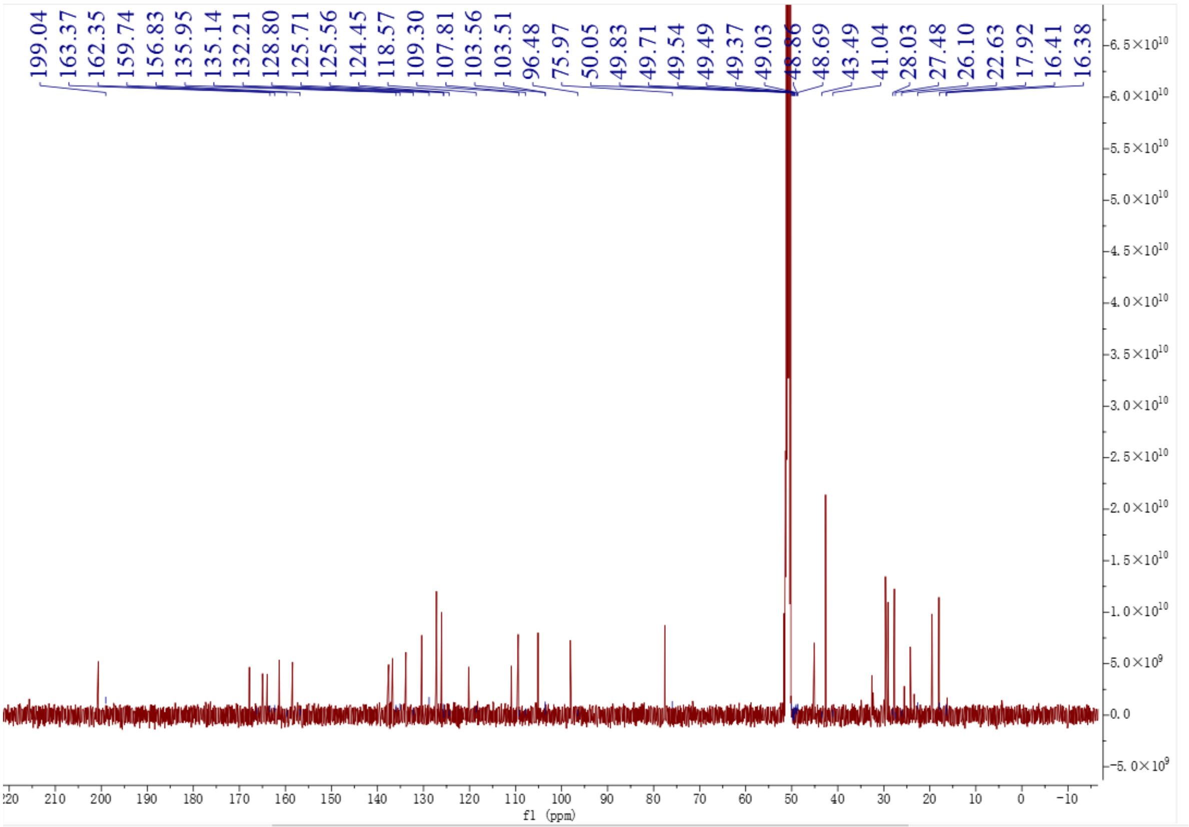

Figure S4: 13C-NMR (151 MHz, MEOD) spectrum of compound **1**

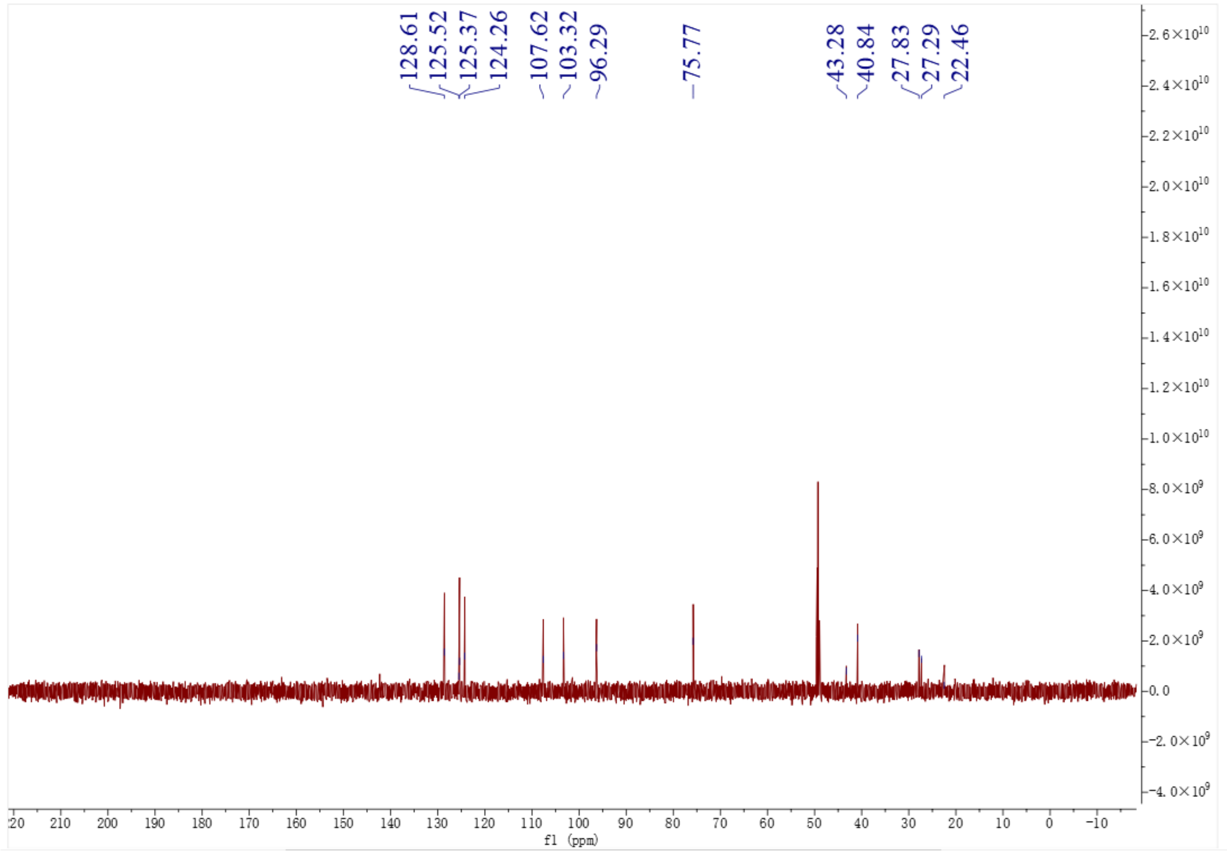

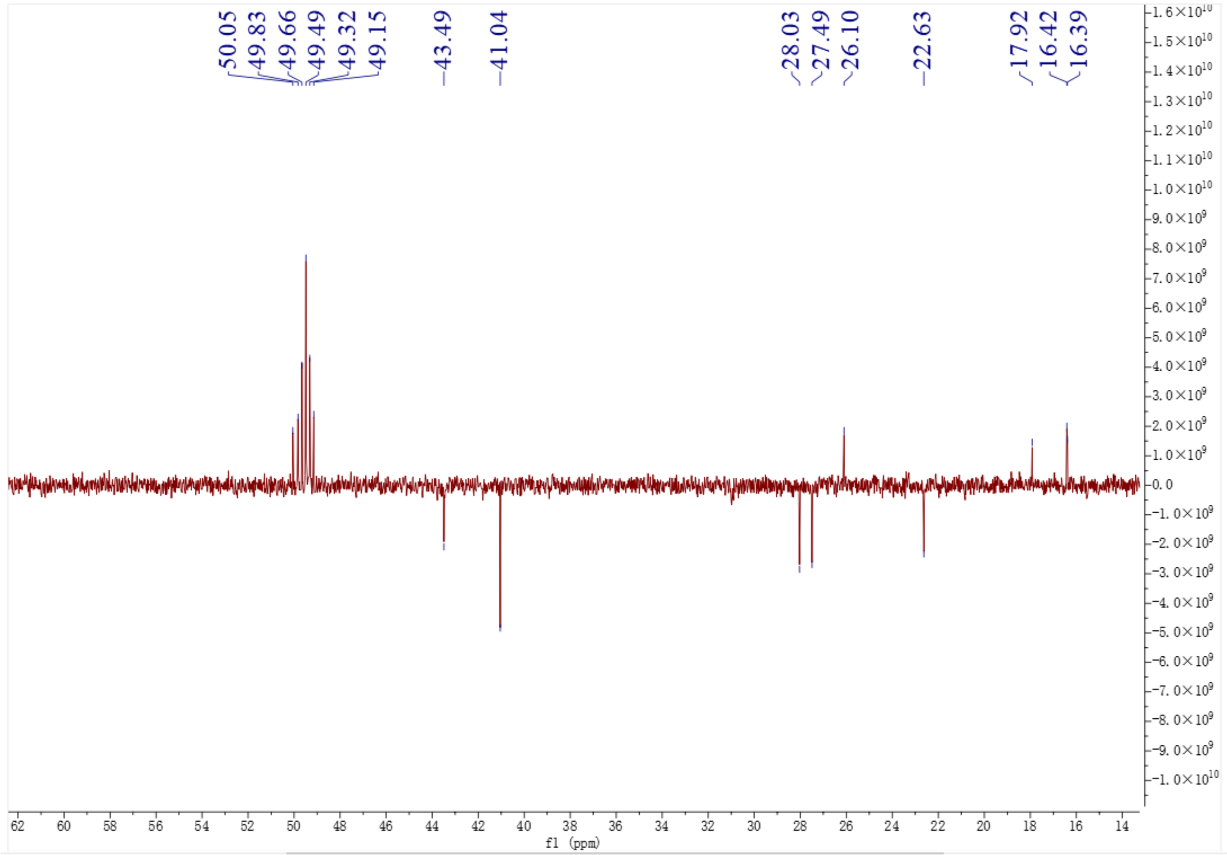

Figure S5: 13C-NMR-DEPT spectrum of compound **1**

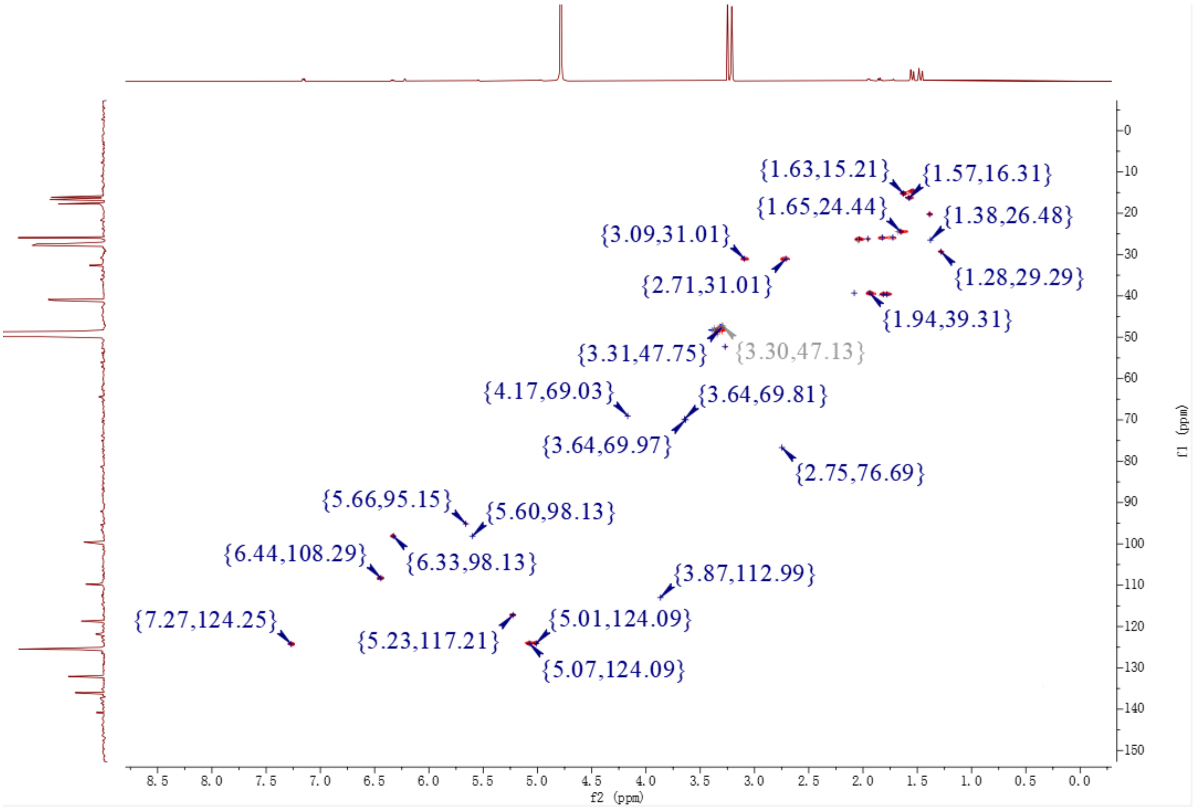

Figure S6: HSQC spectrum of compound **1**

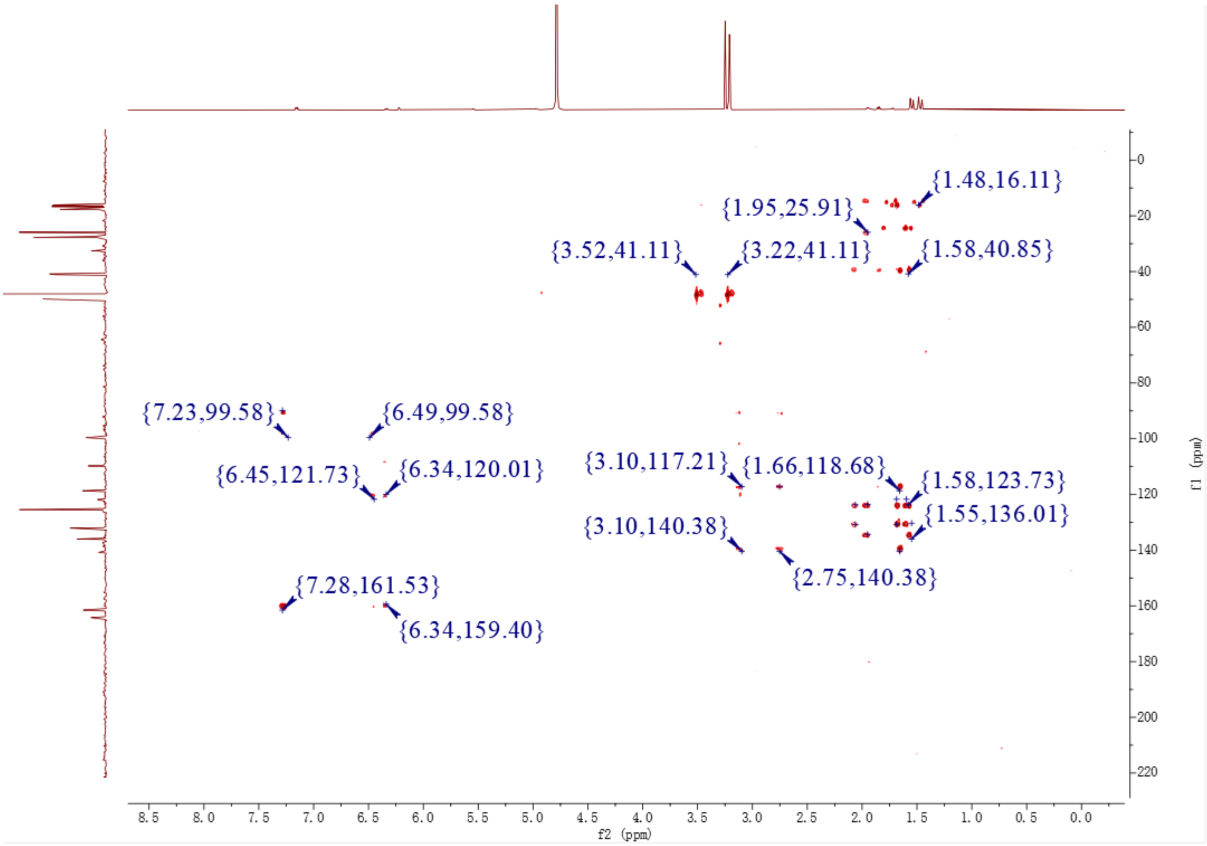

Figure S7: HMBC spectrum of compound **1**

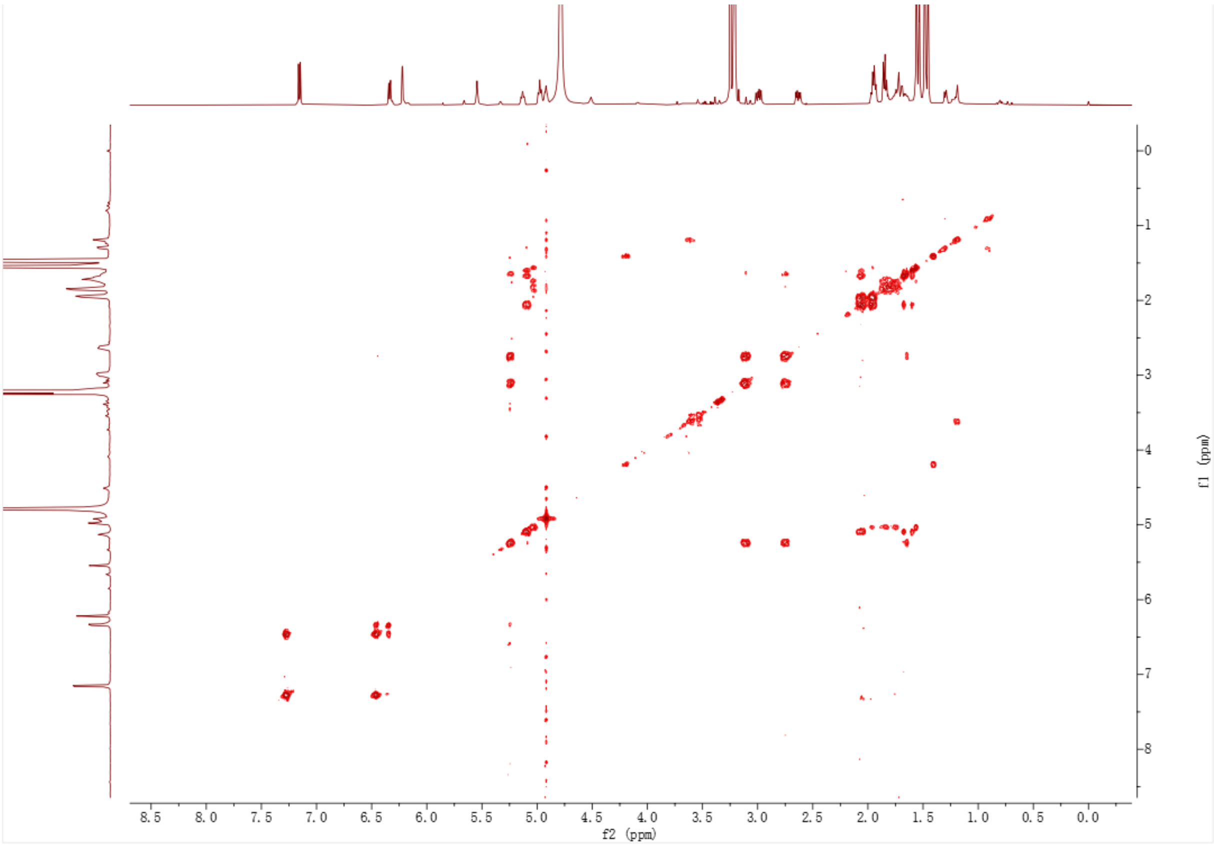

Figure S8: 1H-1H COSY spectrum of compound **1**

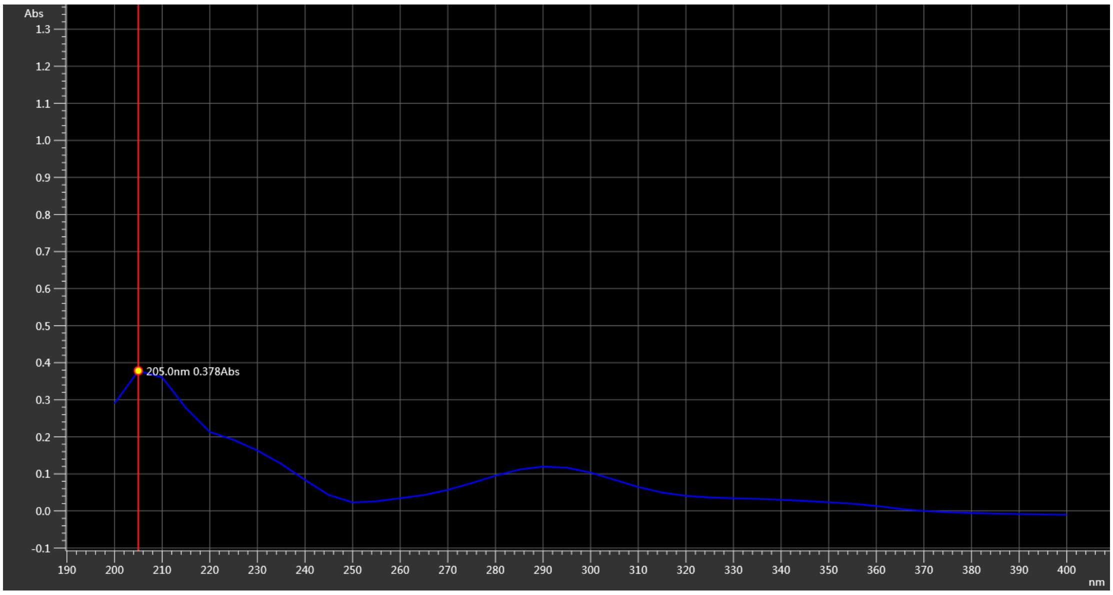

Figure S9: UV spectrum of compound **1**

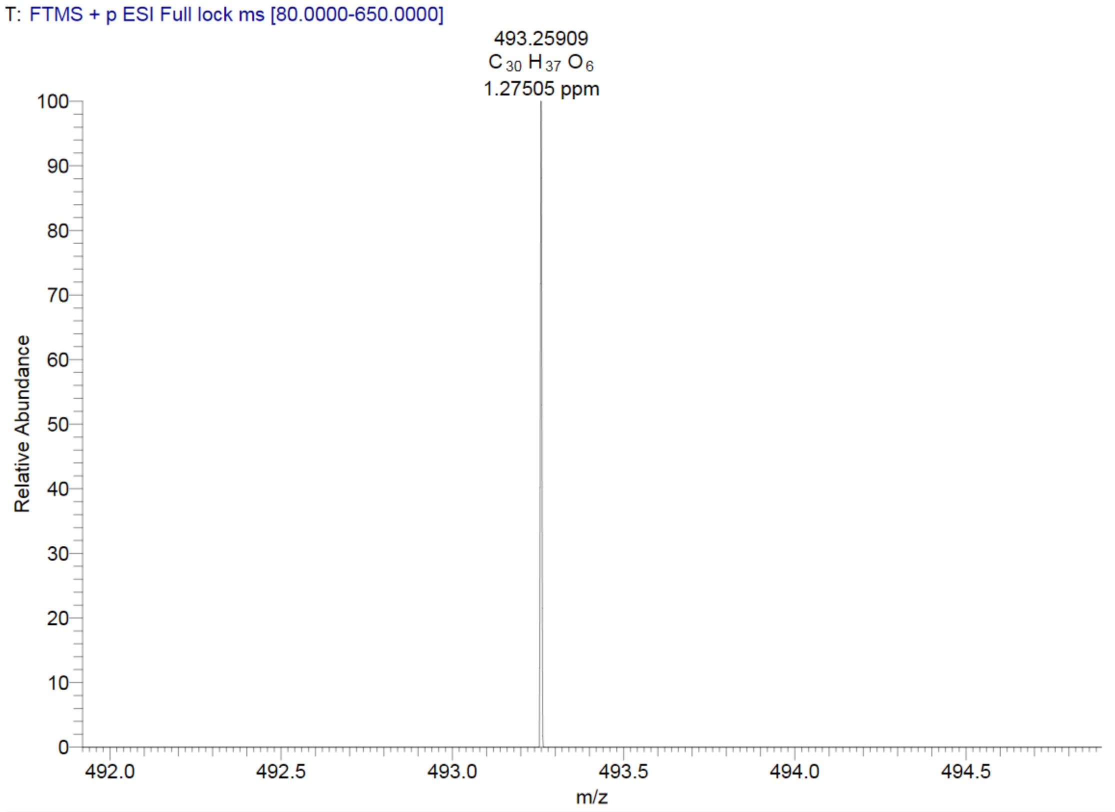

Figure S10: HR-ESI-MS of compound **2**

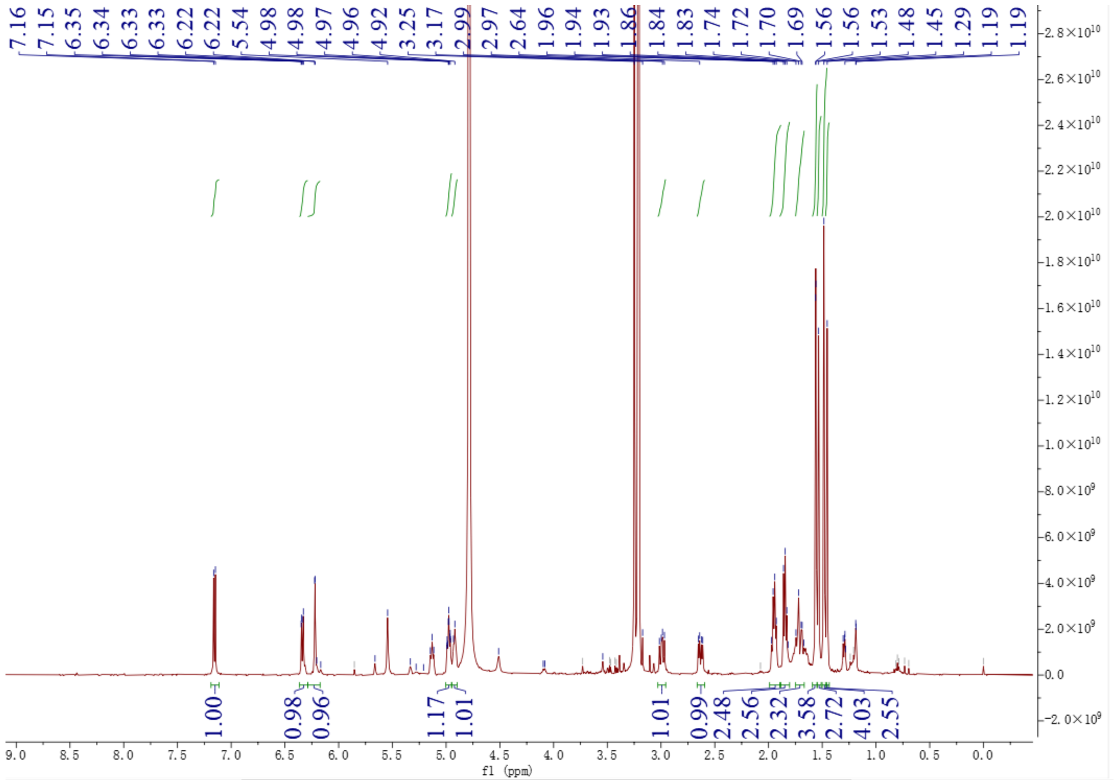

Figure S11: 1H-NMR (500 MHz, MEOD) spectrum of compound **2**

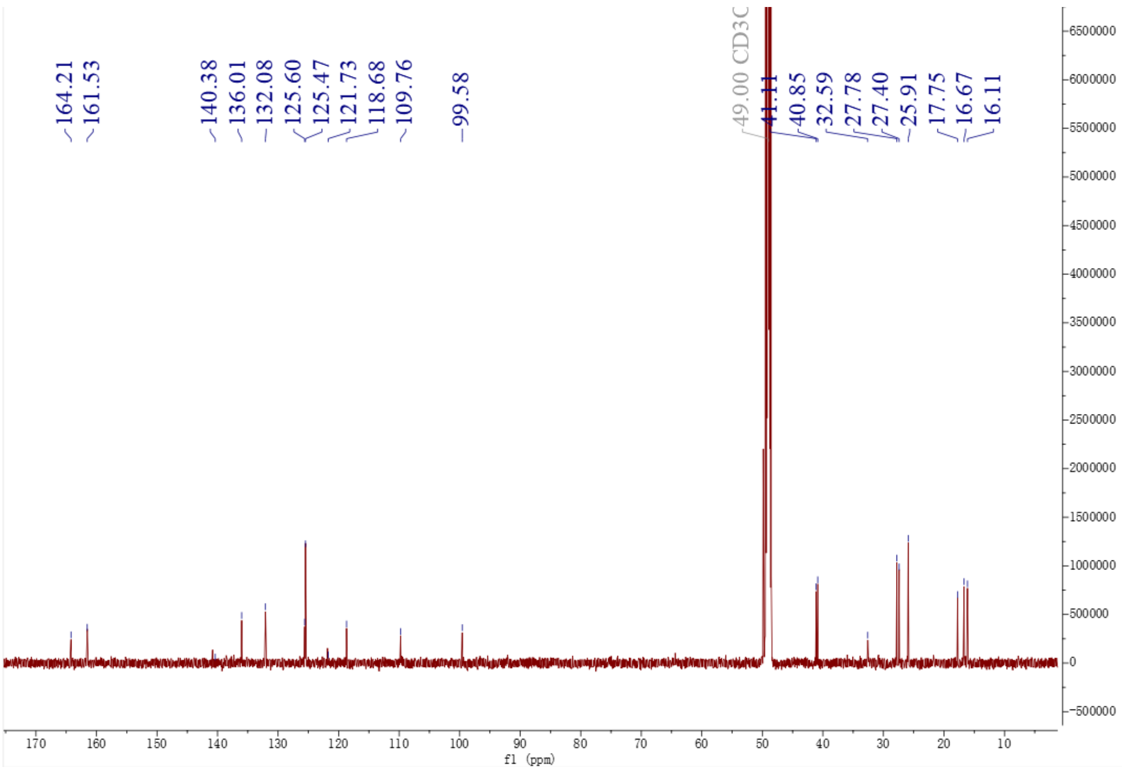

Figure S12: 13C-NMR (126 MHz, MEOD) spectrum of compound **2**

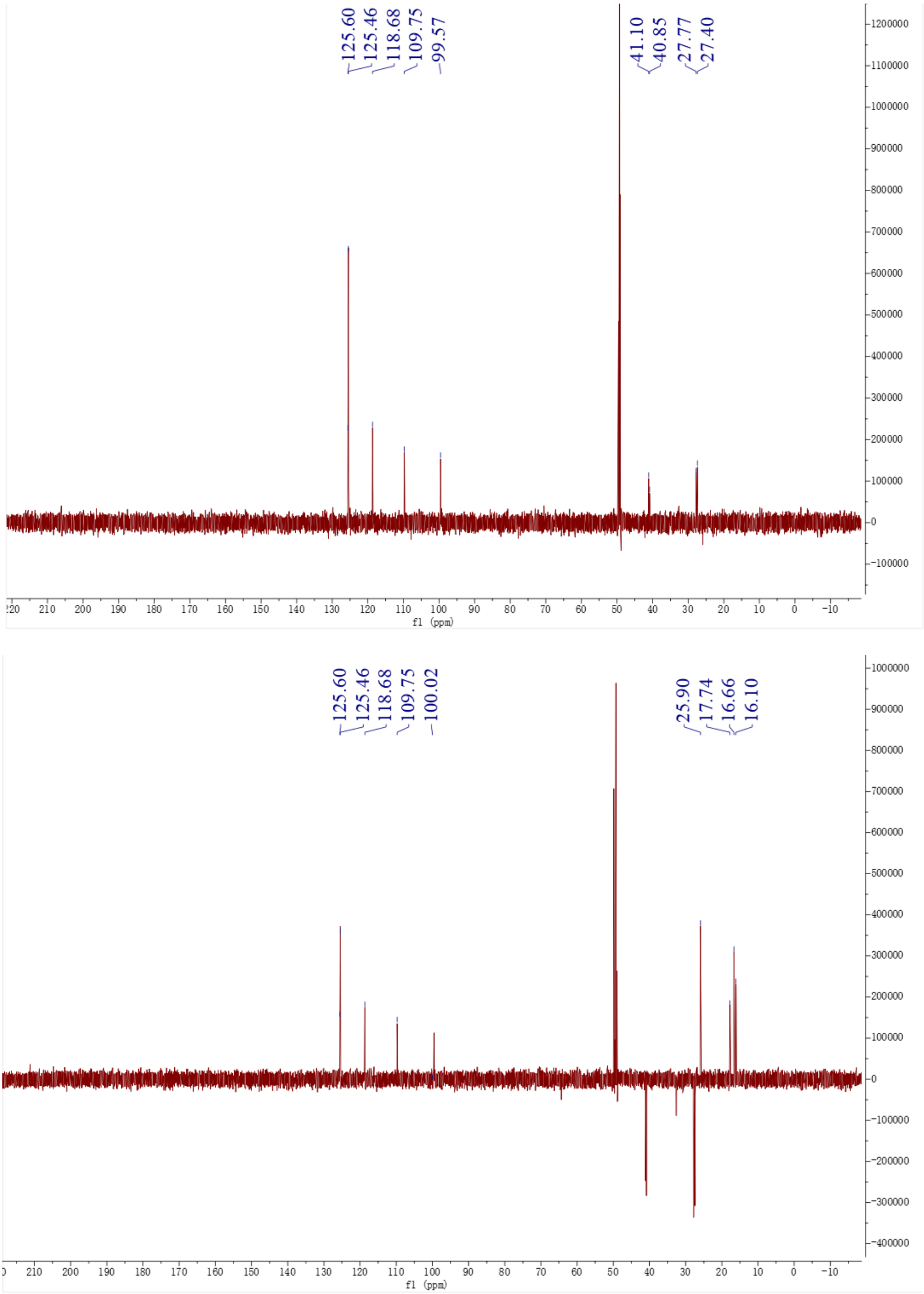

Figure S13: 13C-NMR-DEPT spectrum of compound **2**

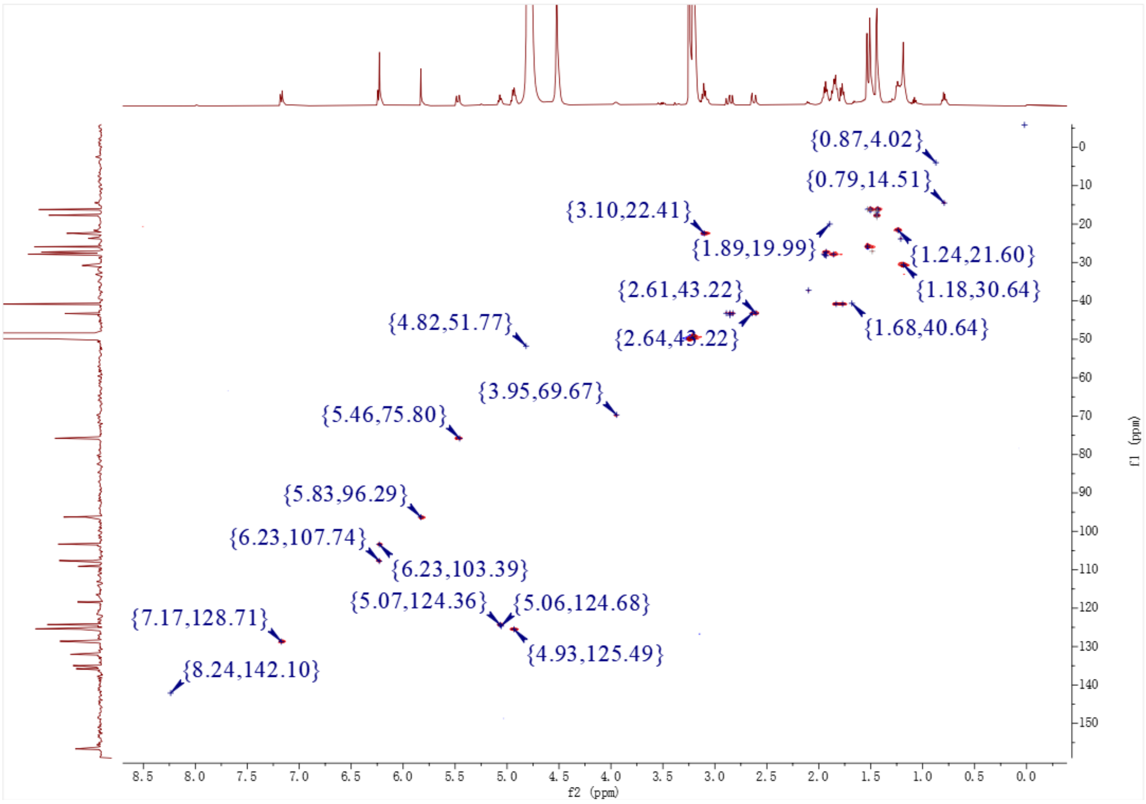

Figure S14: HSQC spectrum of compound **2**

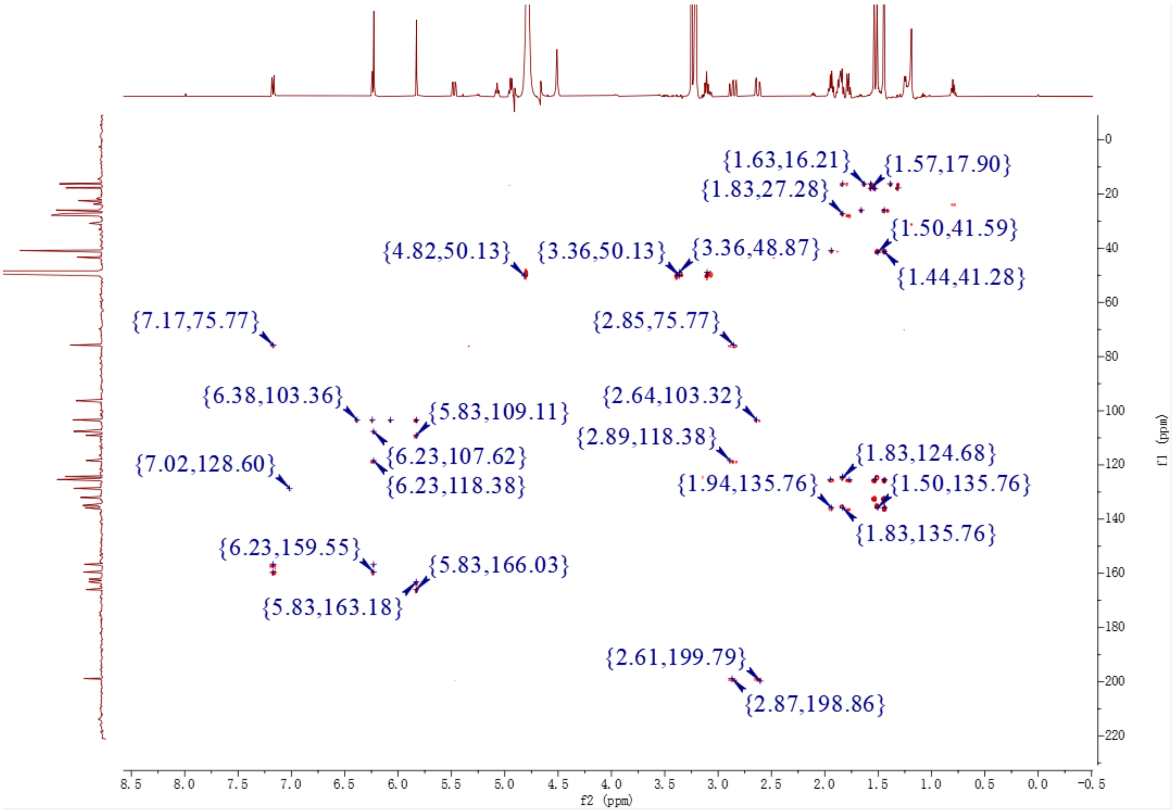

Figure S15: HMBC spectrum of compound **2**

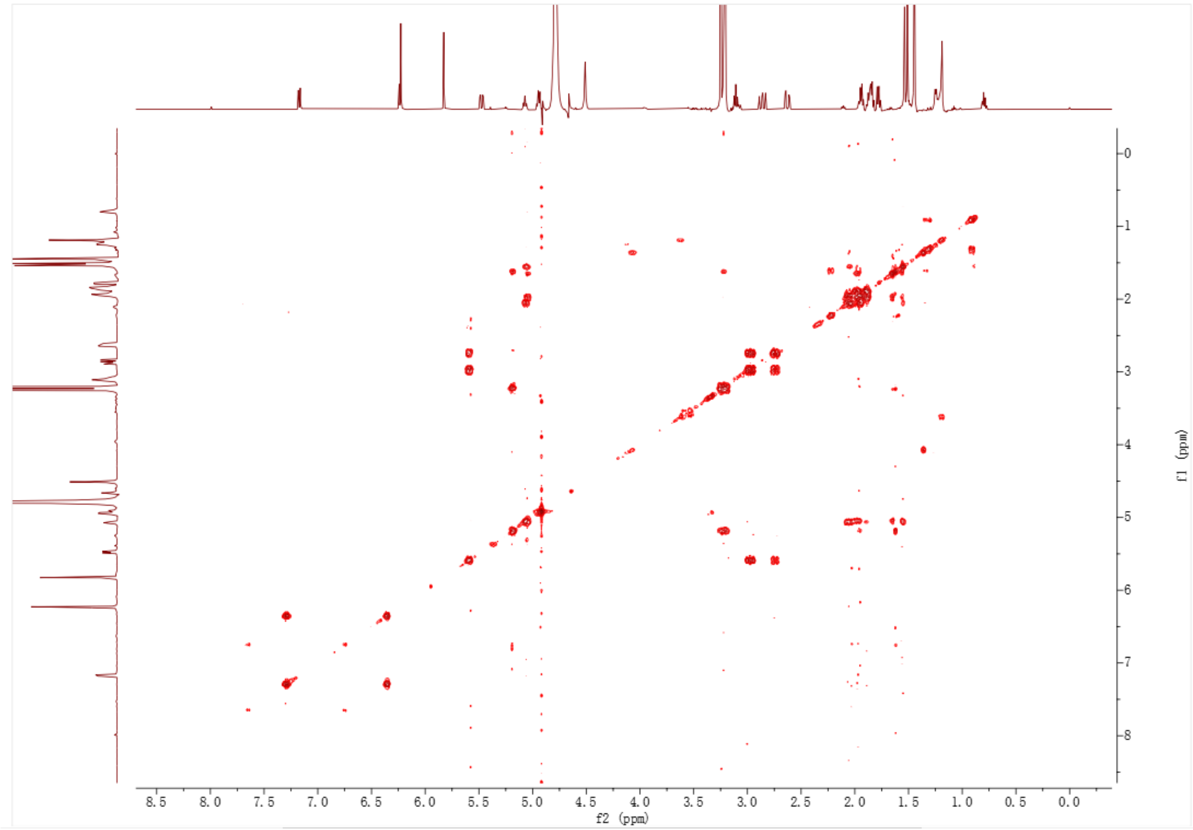

Figure S16: 1H-1H COSY spectrum of compound **2**

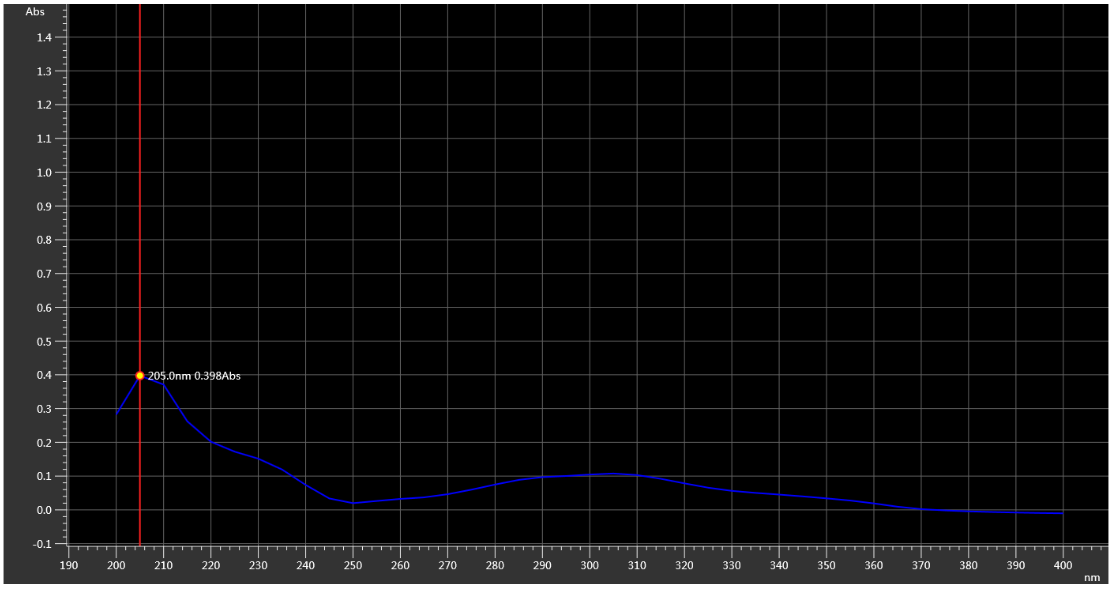

Figure S17: UV spectrum of compound **2**

3

Yellow powder with a molecular formula of $C_{25}H_{26}O_6$. Its 13C-NMR spectrum displays 25 carbon signals. In the low-field region, a flavonoid C-4 ketone carbonyl carbon signal at δ 184.05 is observed. The aromatic region shows typical features of a 5,7,2',4'-tetraoxygenated flavone skeleton. In the high-field region, four methyl carbon signals [δ 25.95, 25.86, 17.76, 17.65] and two methylene carbon signals [δ 24.84, 22.34] are present. In the olefinic region, two pairs of trisubstituted double bond carbon signals [δ 132.44, 122.99 and δ 132.05, 123.43] are observed, confirming the presence of two prenyl groups. Comparison with literature data indicates that the NMR data of this compound are essentially consistent with those reported for Kuwanon C. Therefore, compound 3 was identified as Kuwanon C.

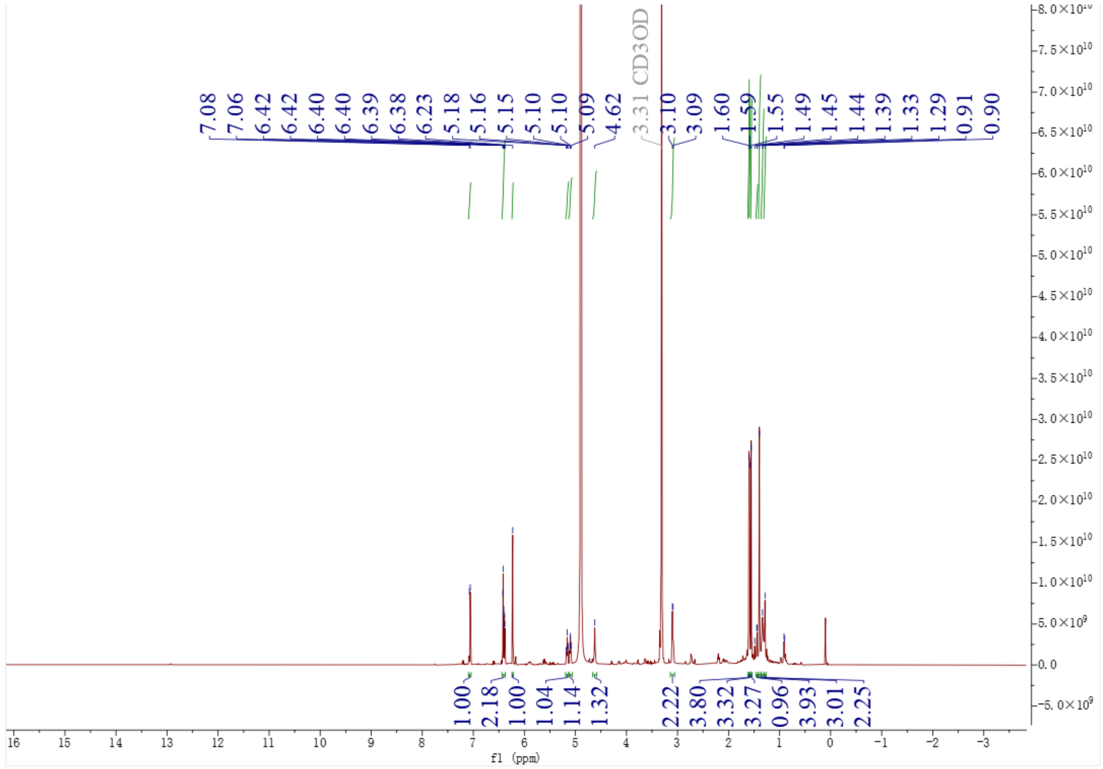

Figure S18: 1H-NMR (500 MHz, MEOD) spectrum of compound 3

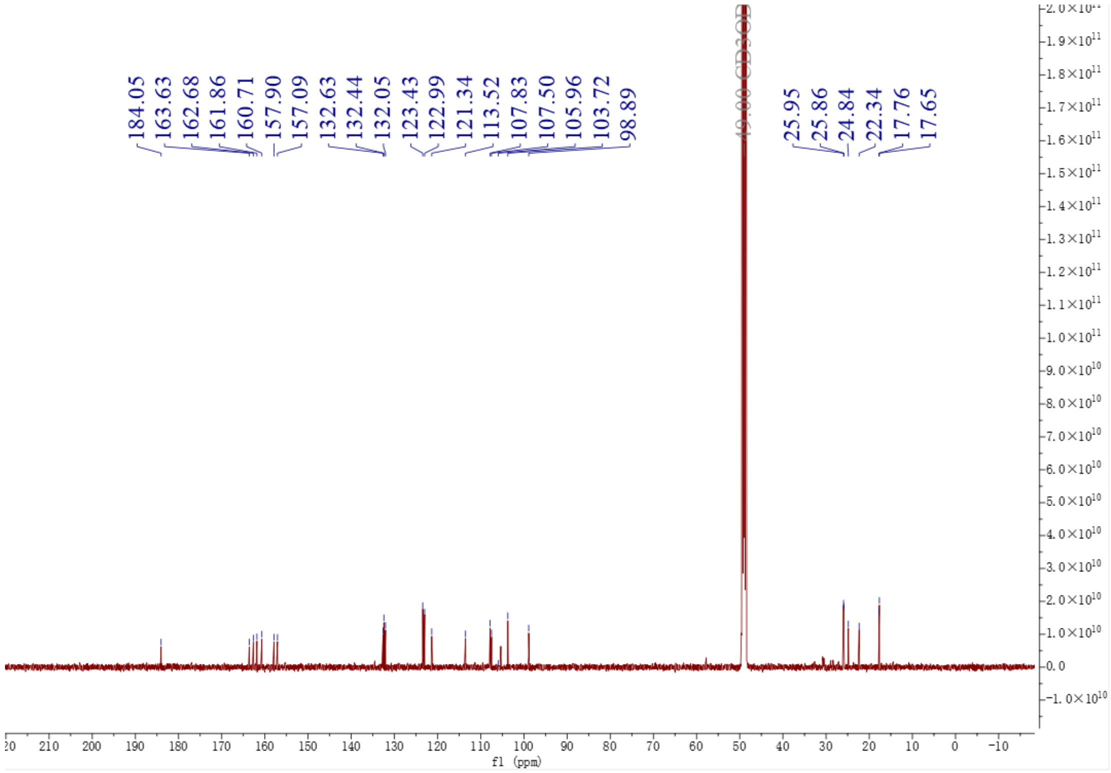

Figure S19: 13C-NMR (126 MHz, MEOD) spectrum of compound **3**

4

Yellow amorphous powder with a molecular formula of $C_{20}H_{20}O_6$. In the low-field region, a typical dihydroflavonoid C-4 ketone carbonyl carbon signal at δC 186.86 is observed. The C-2 carbon signal exhibits a significant downfield shift to (value missing) and is not connected to any proton, indicating the presence of a hemiacetal hydroxyl substitution. Meanwhile, the C-3 carbon signal shifts upfield to (value missing), corresponding to a characteristic aliphatic methine proton at δH 3.17 in the ^{1}H-NMR spectrum, confirming that the core skeleton is a 2-hydroxydihydroflavonoid. In the far downfield region of the ^{1}H-NMR spectrum, a strongly chelated hydroxyl signal at δH 11.49 is present, demonstrating the presence of a free hydroxyl group at the C-5 position on ring A. The aromatic proton signals reveal a meta-coupling system on ring A, indicating a typical 5,7-dihydroxy substitution pattern. In the B-ring region, a spin system consisting of δH 7.28 (1H, d, J = 8.2 Hz, H-6') and overlapping multiplets at higher field is observed, which, combined with multiple oxygenated quaternary carbon signals in the 13C-NMR spectrum, suggests polyhydroxy substitution on the B-ring. Additionally, in the mid- to high-field region, a characteristic set of prenyl group signals is clearly observed, corresponding to five carbon signals. Since the C-3 carbon becomes an aliphatic methine (CH), it confirms that the prenyl group is substituted at the C-3 position of the dihydroflavonoid nucleus. Based on the above spectroscopic features and careful comparison with literature data, the core skeleton signals of this compound are in good agreement with those reported for Sanggenon B. Therefore, the main component compound 4 was identified as Sanggenon B.

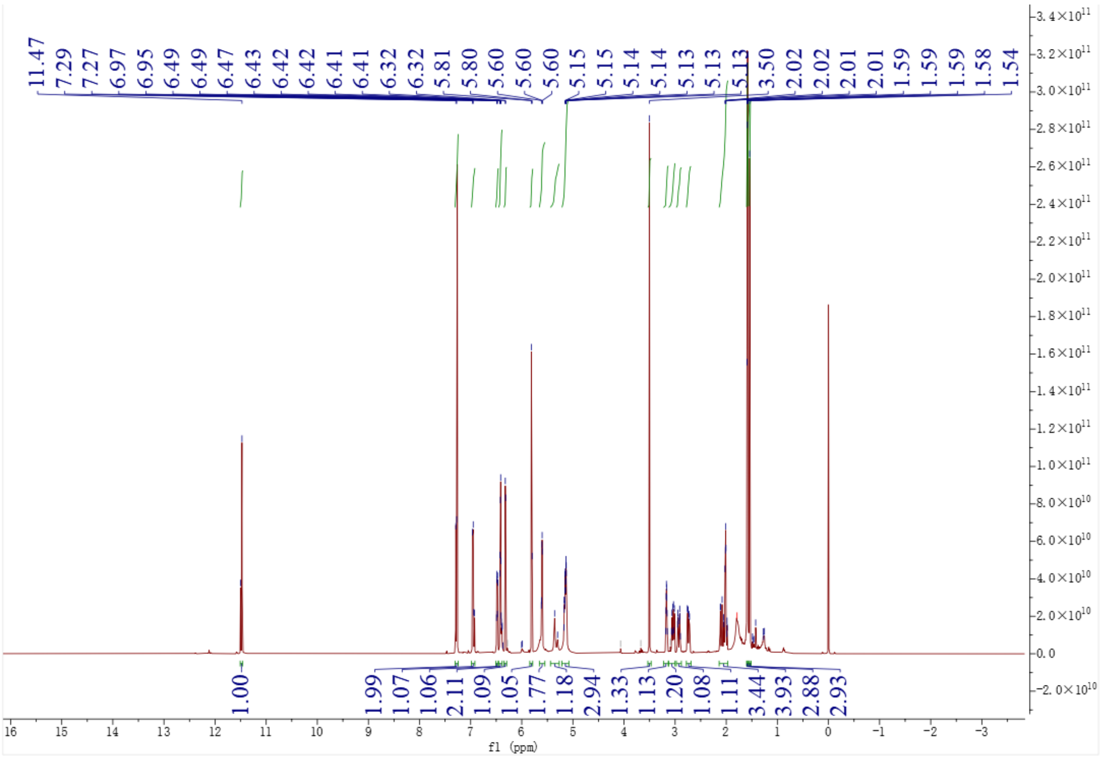

Figure S20: 1H-NMR (500 MHz, CD3OD) spectrum of compound **4**

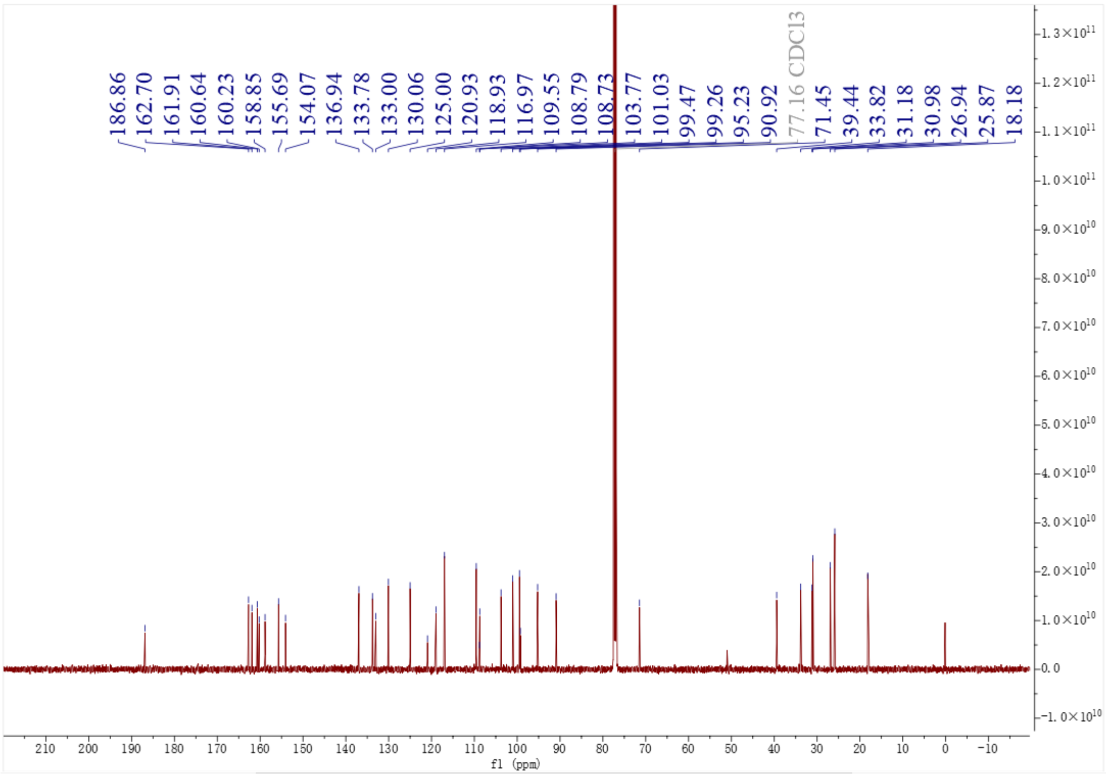

Figure S21: 13C-NMR (126 MHz, CD_3OD) spectrum of compound **4**

5

Yellow powder with a molecular formula of $C_{33}H_{30}O_9$. In the low-field region, a dihydroflavonoid ketone carbonyl carbon signal at δC 188.4 is observed. The carbon signal at δC 103.5 is shifted downfield and is not connected to any proton, indicating the presence of a hemiacetal hydroxyl substitution. The aromatic proton signals reveal a meta-coupling system on ring A, indicating that ring A has a typical 5,7-dihydroxy substitution pattern. The B-ring region contains a spin system consisting of δH 7.20 and overlapping multiplets in the higher field, which, combined with multiple oxygenated quaternary carbon signals in the ^{13}C-NMR spectrum, suggests polyhydroxy substitution on the B-ring. Additionally, in the mid- to high-field region, a characteristic set of prenyl group signals is clearly observed, corresponding to five carbon signals. The carbon signal at δC 32.1 becomes an aliphatic methine, confirming that the prenyl group is substituted at the C-3 position of the dihydroflavonoid nucleus. Based on the above spectroscopic features and comparison with literature data, the core skeleton signals of this compound are in good agreement with those reported for 2,7,9,10a-tetrahydroxy-8-(5-hydroxy-9-methyl-8-oxatricyclo[7.3.1.$0^{2,7}$]trideca-2(7),3,5,10-tetraen-11-yl)-4b-(3-methylbut-2-enyl)-4b,10a-dihydro-5,11-dioxabenzo[b]fluoren-10-one. Therefore, the main component compound 5 was identified as 2,7,9,10a-tetrahydroxy-8-(5-hydroxy-9-methyl-8-oxatricyclo[7.3.1.$0^{2,7}$]trideca-2(7),3,5,10-tetraen-11-yl)-4b-(3-methylbut-2-enyl)-4b,10a-dihydro-5,11-dioxabenzo[b]fluoren-10-one.

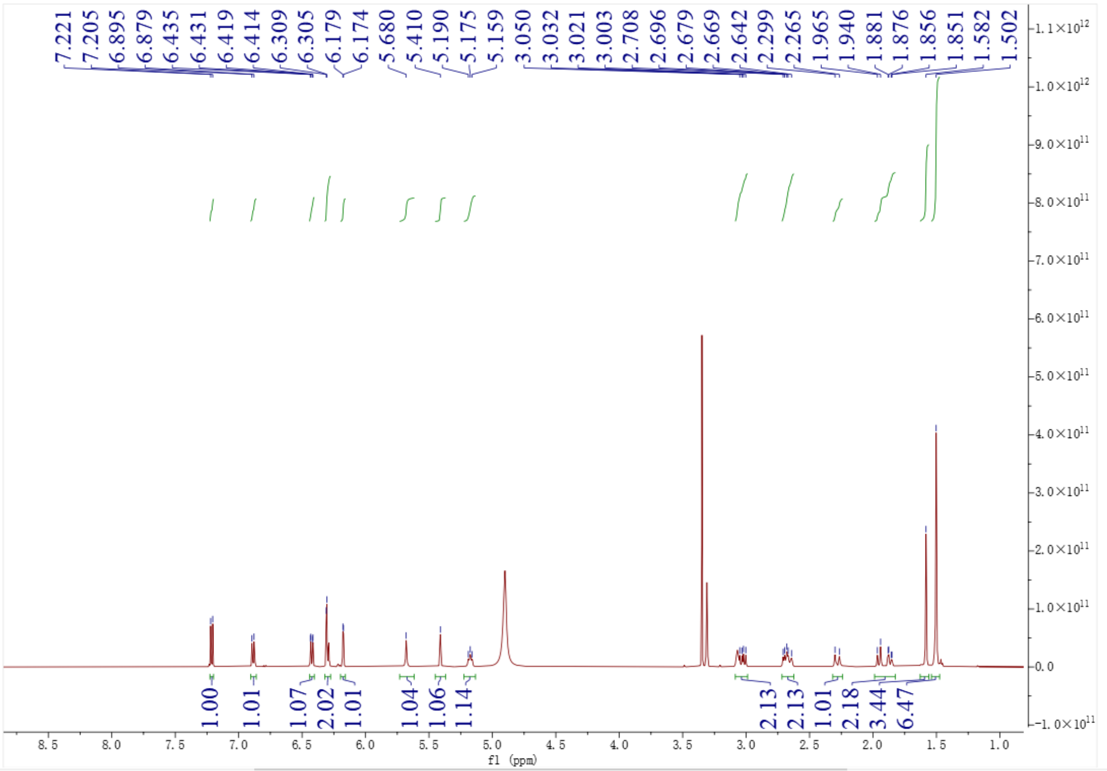

Figure S22: 1H-NMR (500 MHz, MEOD) spectrum of compound **5**

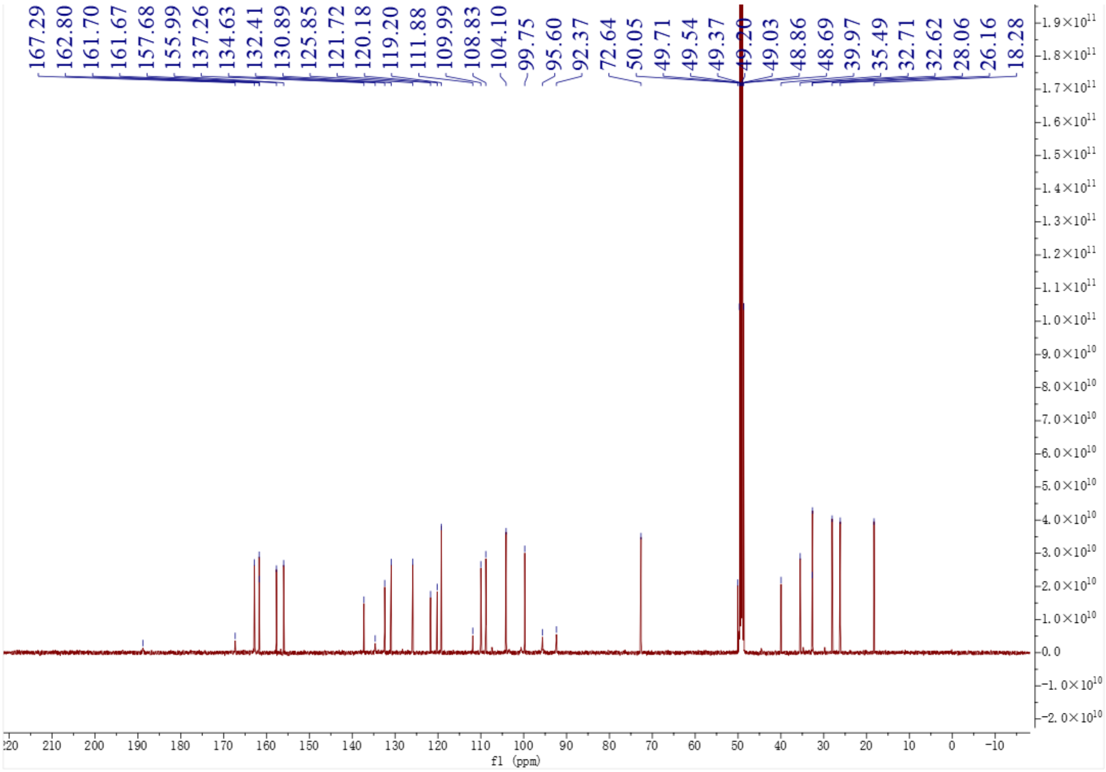

Figure S23: 1H-NMR (500 MHz, MEOD) spectrum of compound **5**

6

Yellow powder with a molecular formula of $C_{25}H_{26}O_5$. Its ^{13}C-NMR spectrum shows a flavonoid C-4 ketone carbonyl carbon signal at δC 183.80. Five oxygenated aromatic quaternary carbon signals [δC 166.71, 166.56, 163.27, 160.60, 159.49] are observed between δC 159 and 167. Combined with the aromatic methine signals at δC 100.33 and 95.18 in the mid-field region, this indicates that the core skeleton of the compound is a 5,7,4'-trioxygenated flavone. In the high-field aliphatic region, three methyl carbon signals [δC 25.85, 17.78, 16.24] and three methylene carbon signals [δC 40.87, 29.02, 27.79] are present. In the olefinic region, two pairs of trisubstituted double bond carbon signals [δC 137.67, 123.11; 132.35, 125.28] are observed. Comparison with literature data indicates that the NMR data of this compound are completely consistent with those reported for Kuwanon S. Therefore, compound 6 was identified as Kuwanon S.

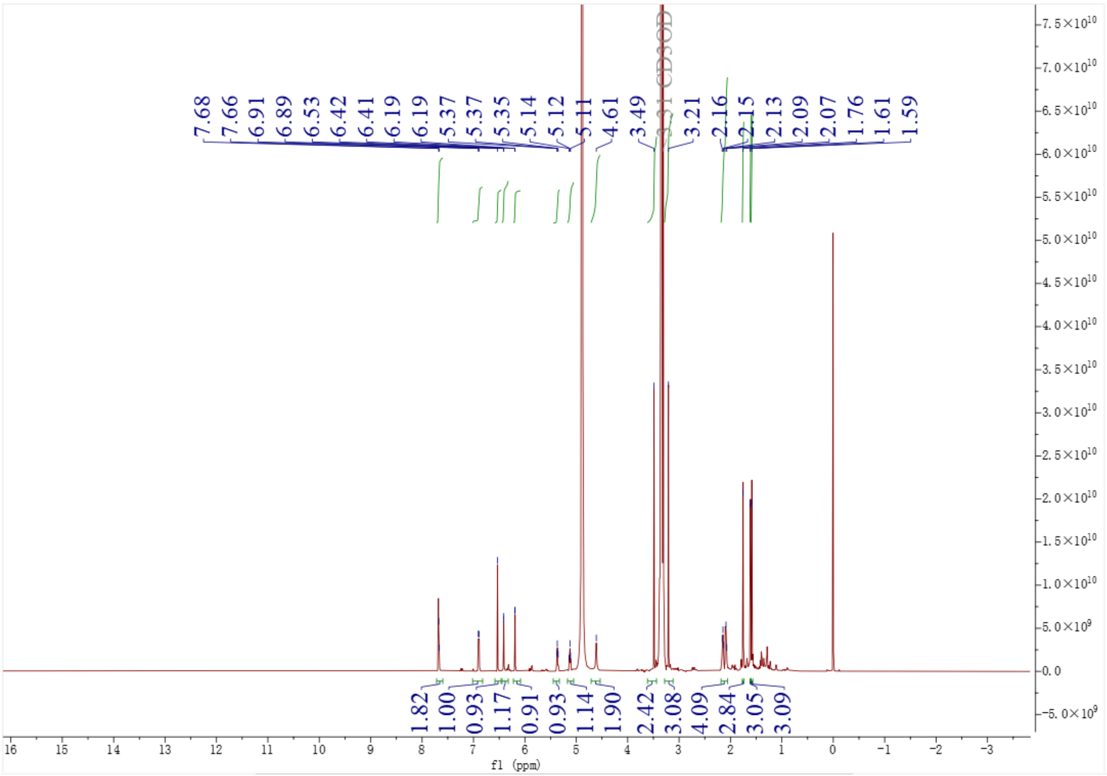

Figure S24: 1H-NMR (500 MHz, MEOD) spectrum of compound **6**

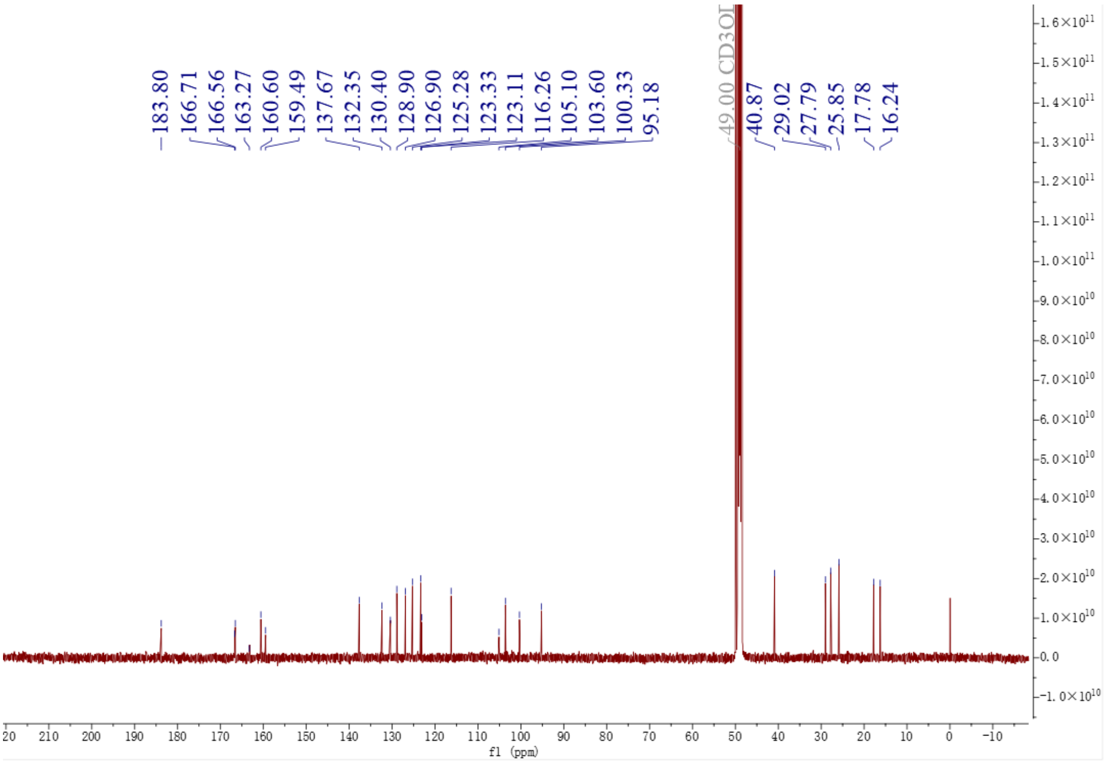

Figure S25: 13C-NMR (126 MHz, MEOD) spectrum of compound **6**

7

Yellow powder with a molecular formula of $C_{25}H_{28}O_6$. Its ^{13}C-NMR spectrum shows a dihydroflavonoid ketone carbonyl carbon signal at δC 198.40 in the low-field region. In the mid- to high-field region, an oxygenated methine carbon signal at δC 76.06 and an aliphatic methylene carbon signal at δC 43.18 are observed. In the ^{1}H-NMR spectrum, signals at δH 5.60, 3.04, and 2.70 indicate that the core skeleton of the compound is a dihydroflavonoid. In the aromatic region of the ^{1}H-NMR spectrum, a pair of meta-coupled proton signals at δH 5.90 and 5.87 are observed on ring A, indicating that ring A has a 5,7-dihydroxy substitution pattern. In the B-ring region, two singlet proton signals at δH 7.07 and 6.64 are present, suggesting that the two protons on the B-ring are para-oriented. Additionally, in the high-field region, three singlet methyl signals at δH 1.69, 1.62, and 1.57 are observed, and the B-ring carbon signal at δC 120.53 is shifted downfield. Comparison with literature data indicates that the NMR data of this compound are essentially consistent with those reported for Kuwanon E. Therefore, compound 7 was identified as Kuwanon E.

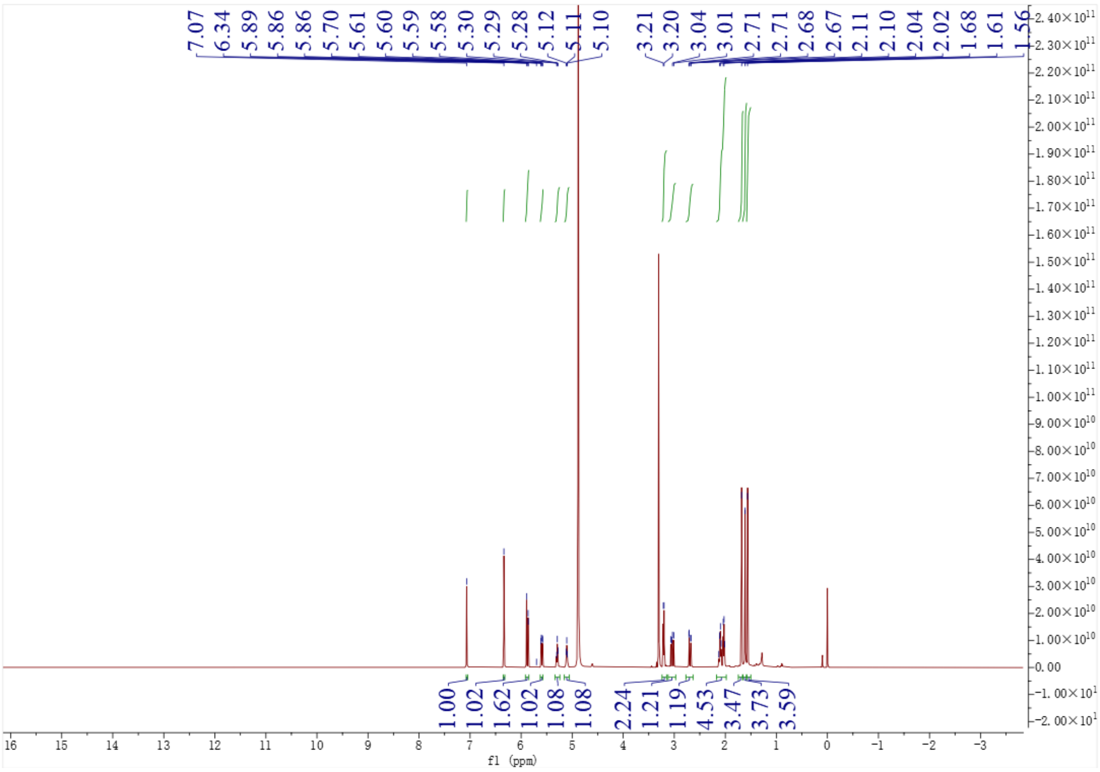

Figure S26: 1H-NMR (500 MHz, MEOD) spectrum of compound **7**

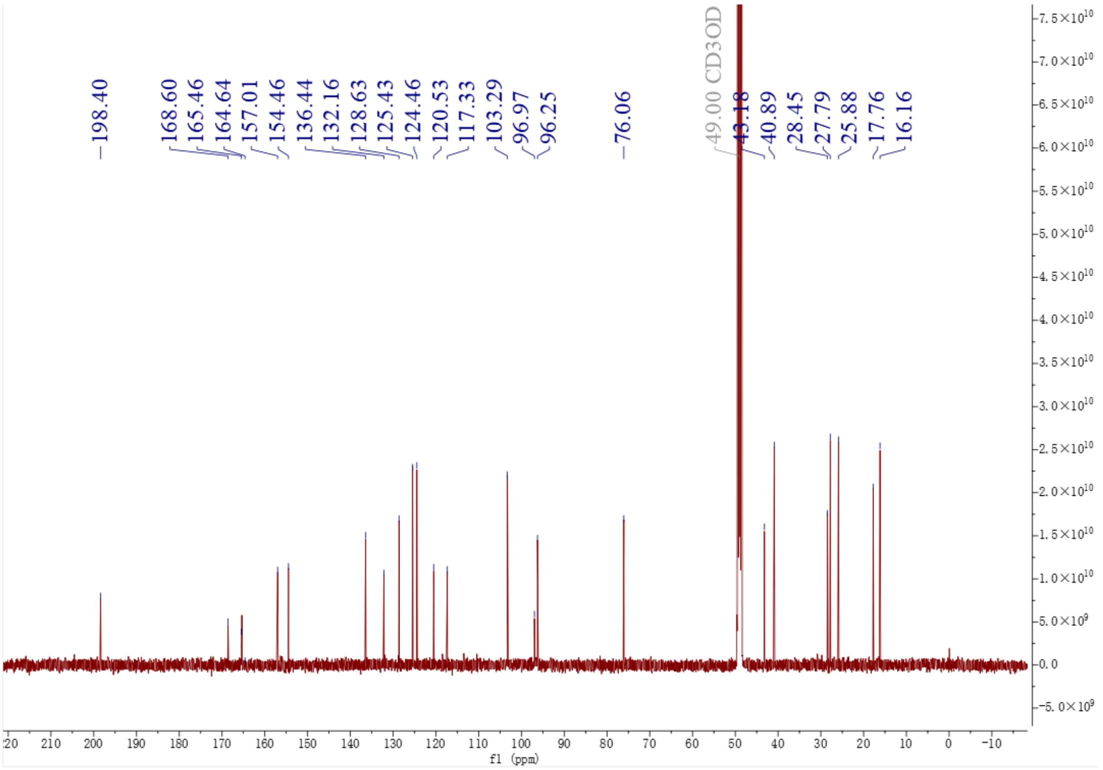

Figure S27: 13C-NMR (126 MHz, MEOD) spectrum of compound **7**

8

Pale yellow powder with a molecular formula of $C_{10}H_8O_4$. The ^{1}H-NMR spectrum shows a set of characteristic cis-double bond proton signals at δH 6.19 and δH 7.84, which are hallmark signals of the α-pyrone ring of the coumarin core. In the aromatic region, two isolated singlet proton signals at δH 6.76 and δH 7.10 are observed, indicating that the benzene ring of the coumarin has a 6,7-disubstituted pattern. Additionally, a distinct methoxy signal at δH 3.90 (3H, s) is present in the high-field region. ^{13}C-NMR shows an α-pyrone lactone carbonyl carbon at δC 164.1 in the low-field region; δC 153.1, 151.5, and 147.1 are typical oxygenated aromatic quaternary carbon signals. Together with the methoxy carbon signal at δC 56.8, these indicate the presence of one methoxy group and one free phenolic hydroxyl group attached to the core skeleton. Based on the above spectroscopic features, this compound is identified as 6-methoxy-7-hydroxycoumarin. Comparison of the above NMR data with literature reports shows that this compound is consistent with scopoletin. Therefore, compound 8 was identified as scopoletin.

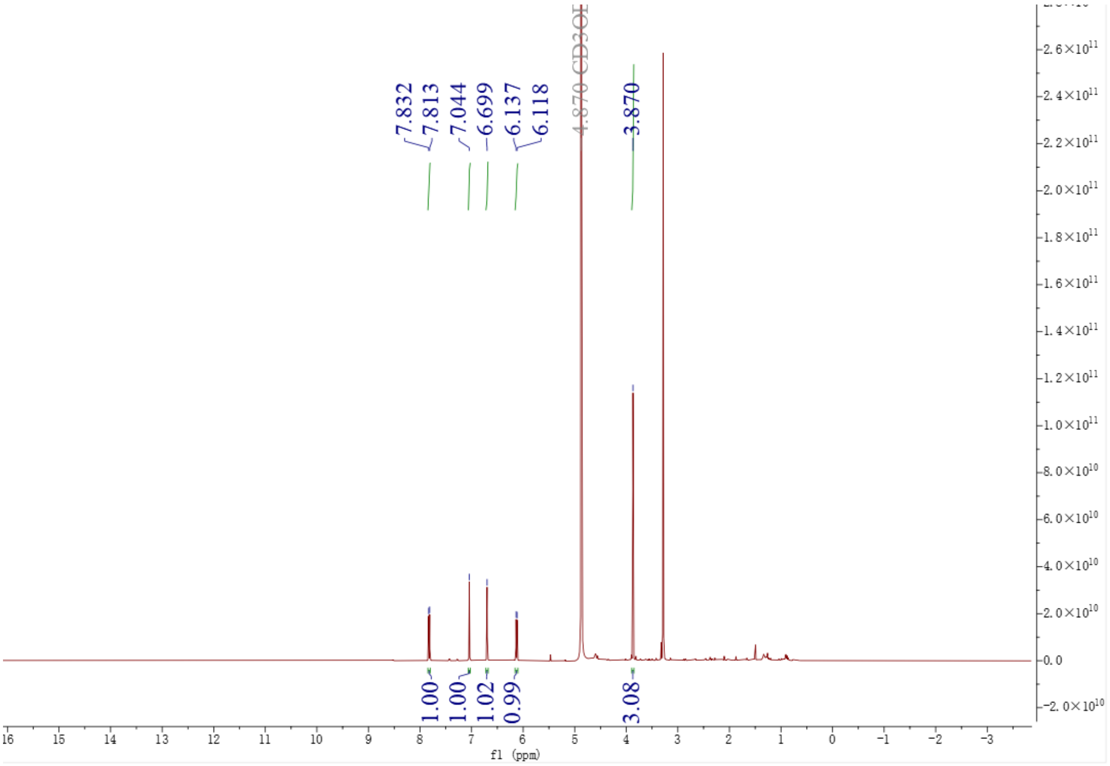

Figure S28: 1H-NMR (500 MHz, MEOD) spectrum of compound **8**

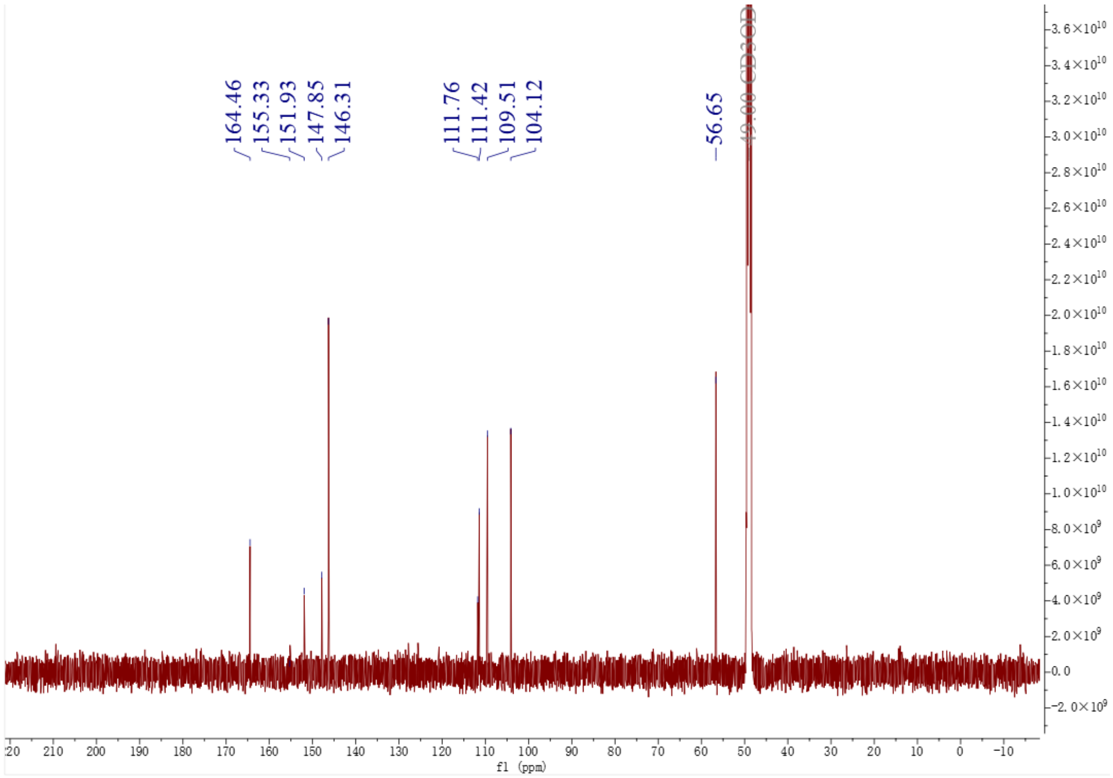

Figure S29: 13C-NMR (126 MHz, MEOD) spectrum of compound **8**

9

White powder with a molecular formula of $C_{10}H_8O_3$. The ^{1}H-NMR spectrum shows a set of characteristic cis-double bond proton signals at δH 6.24 and δH 7.62, indicating the presence of an α-pyrone ring structure of the coumarin core. The aromatic protons exhibit a typical ABX coupling system: δH 7.36 and δH 6.83 show ortho coupling, while H-6 shows meta coupling with δH 6.80 in the downfield region, indicating that the benzene ring is monosubstituted at the 7-position. Additionally, a methoxy singlet signal at δH 3.86 is present in the high-field region. The ^{13}C-NMR spectrum shows the α-pyrone lactone carbonyl carbon at δC 161.2 in the low-field region; δC 162.8 is significantly shifted downfield, which, together with the aliphatic carbon signal at δC 55.8, indicates that a methoxy group is attached to the C-7 position. Based on the above spectroscopic features, this compound is identified as 7-methoxycoumarin. Comparison of its NMR data with literature reports shows consistent spectroscopic characteristics. Therefore, compound 9 was identified as 7-methoxycoumarin.

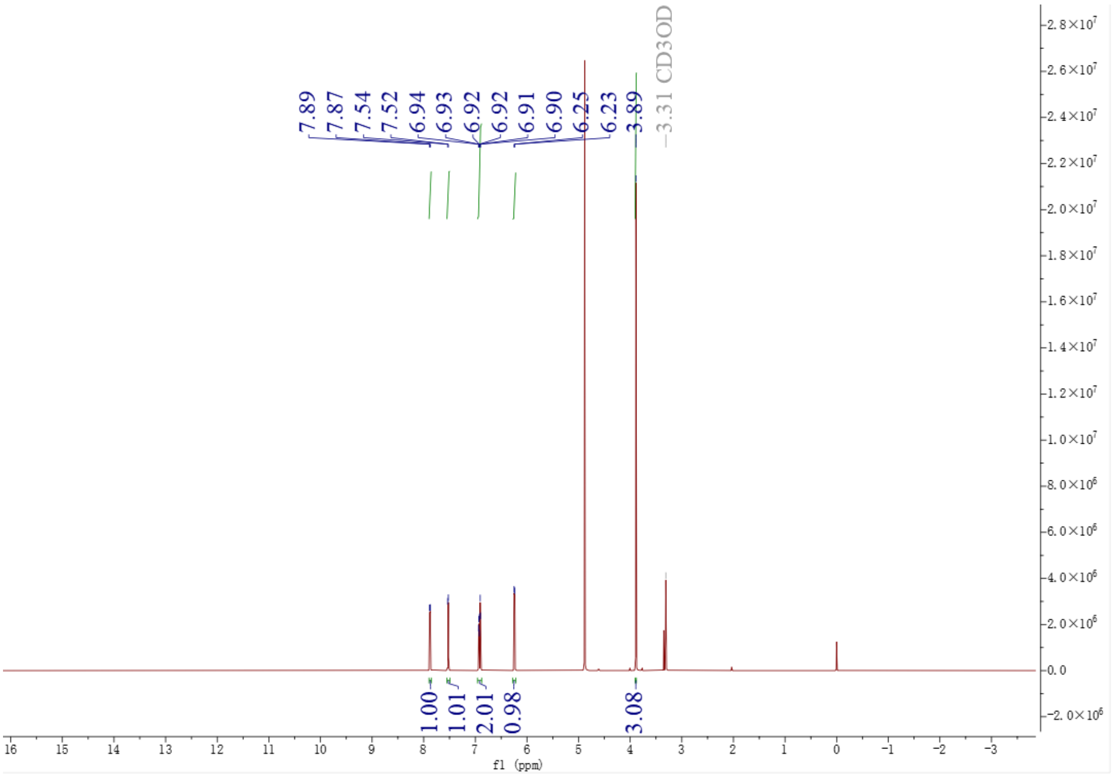

Figure S30: 1H-NMR (500 MHz, MEOD) spectrum of compound **9**

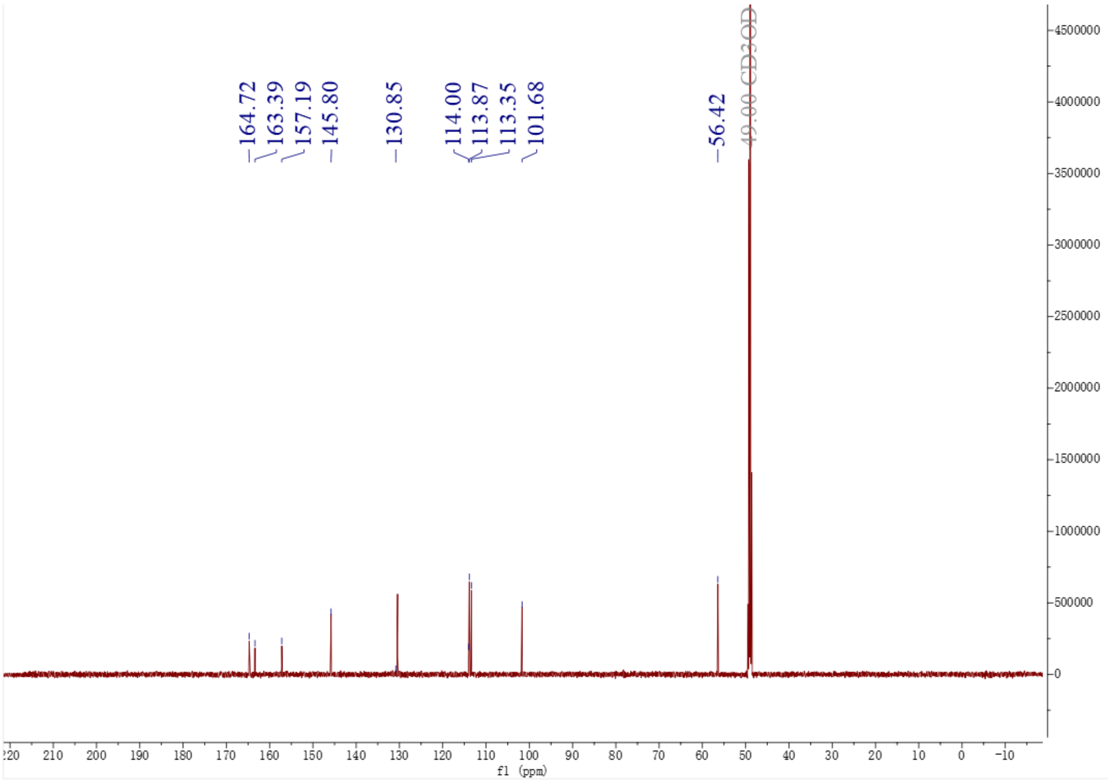

Figure S31: 13C-NMR (151 MHz, MEOD) spectrum of compound **9**

10

Yellow powder with a molecular formula of $C_{25}H_{22}O_6$. Its ^{1}H-NMR (500 MHz, MeOD) spectrum shows four singlet methyl signals at δH 1.47, 1.70, and 1.95 in the high-field region. In the mid- to low-field region, four mutually coupled olefinic proton signals are observed, constituting two sets of 2,2-dimethylpyran ring cis-double bond systems at δH 6.84, 5.73 and δH 6.16, 5.42. In the aromatic region, signals at δH 7.66, 6.55, and 6.32 indicate a 2',4'-disubstitution pattern on the B-ring, along with an isolated aromatic proton singlet at δH 6.32. Its ^{13}C-NMR (126 MHz, MeOD) spectrum shows a flavonoid carbonyl carbon signal at δC 179.9, along with six oxygenated sp^2 hybridized carbon signals at δC 165.2, 163.1, 160.6, 159.9, 157.5, and 152.7. The signals at δC 79.4 and 70.8 further confirm the presence of oxygenated quaternary carbons of two pyran rings. Comparison with literature data indicates that the NMR data of this compound are in good agreement with those reported for Cyclomorusin. Therefore, compound 10 was identified as Cyclomorusin.

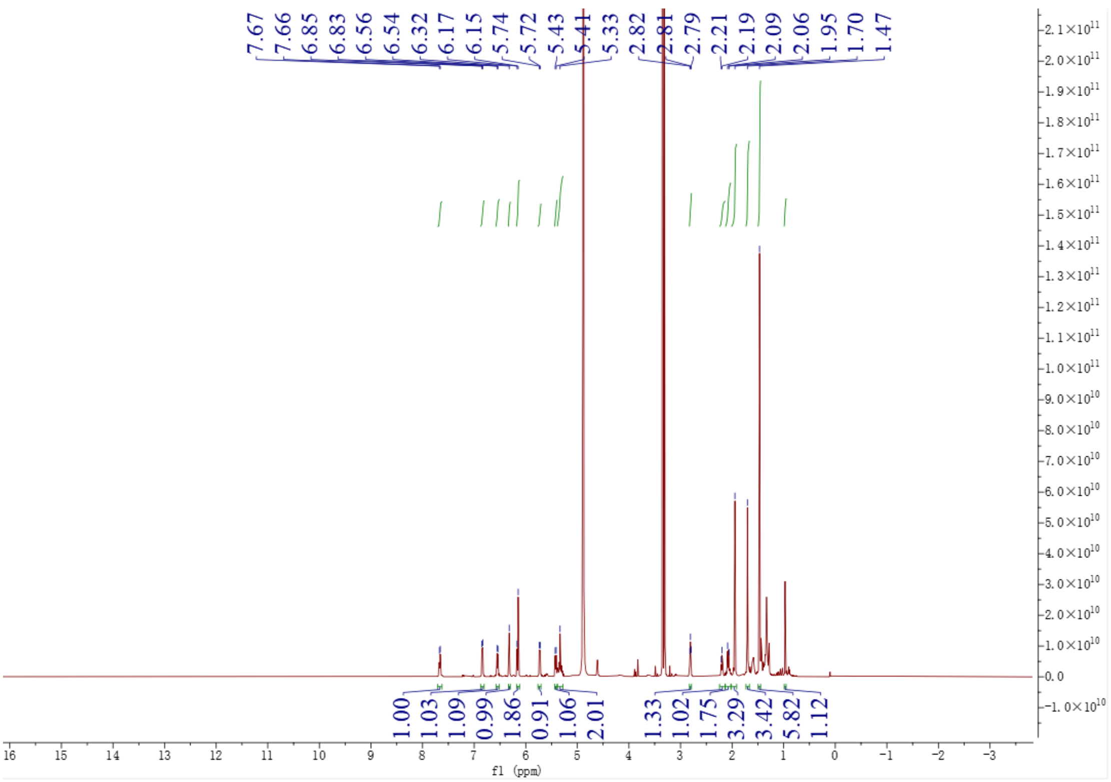

Figure S32: 1H-NMR (500 MHz, MEOD) spectrum of compound **10**

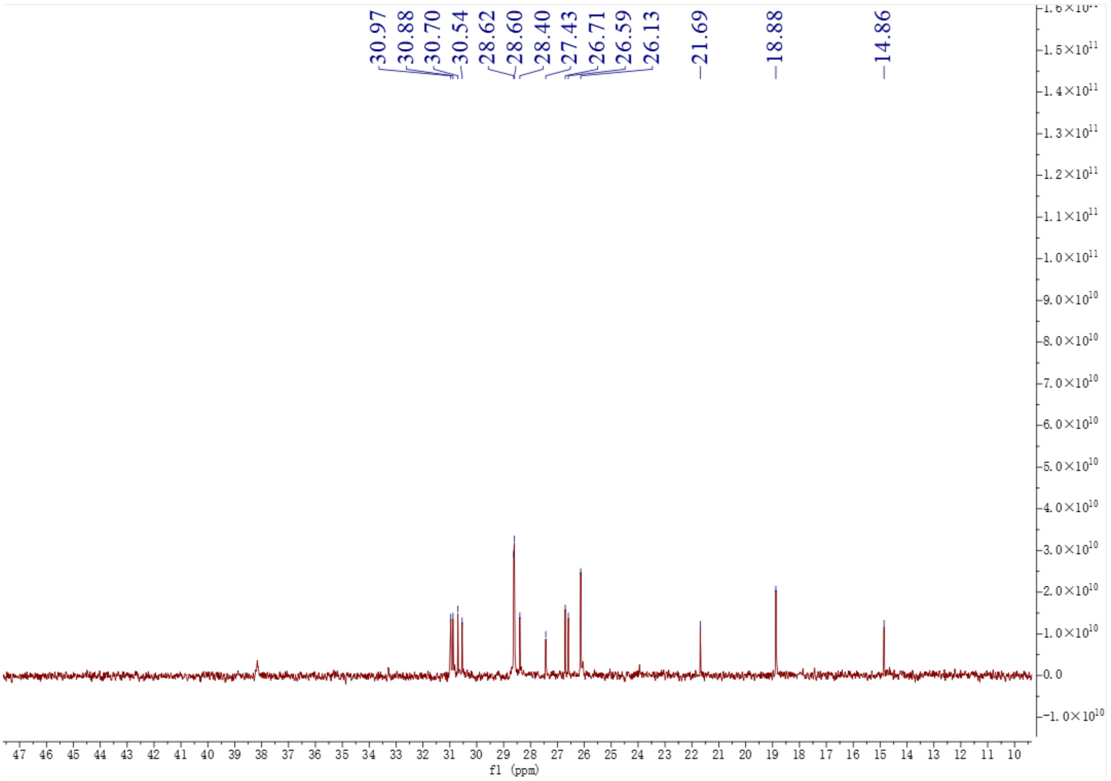

Figure S33: 13C-NMR (126 MHz, MEOD) spectrum of compound **10**

11

Yellow powder with a molecular formula of $C_{24}H_{26}O_4$. Its ^{13}C-NMR spectrum

shows the presence of a prenyl group [δC 133.38, 121.57, 27.26, 25.90, 17.77]. Combined with the overlapping double carbon signals at δC 156.07 and δC 106.99 arising from chemical equivalence, it is inferred that the B-ring has a symmetrical 1,3,4,5-tetrasubstituted pattern. In the low-field region, four oxygenated carbon signals are observed at δC 157.21, 156.07, 154.89, and 154.68, consistent with the characteristics of a 6-hydroxybenzofuran and a 3,5-dihydroxybenzene ring. In its ^{1}H-NMR spectrum, signals at δH 7.36, 6.76, and 6.87 indicate that ring A is an AMX spin coupling system, corresponding to a 6-hydroxy substitution. An isolated singlet at δH 6.47 is observed on the B-ring, indicating that the B-ring has a 3,5-dihydroxy-4-substituted phenyl structure. Based on the above spectroscopic data and comparison with literature reports, the data of this compound are consistent with those of Mulberrofuran V. Therefore, compound 11 was identified as Mulberrofuran V.

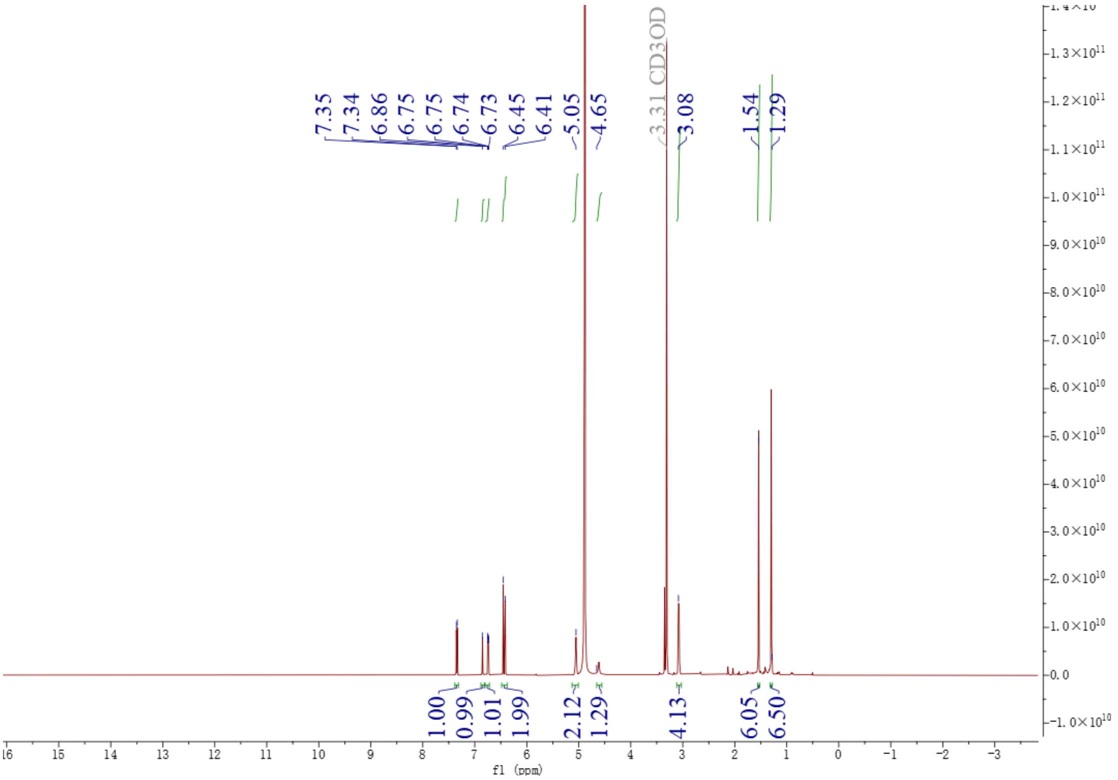

Figure S34: 1H-NMR (500 MHz, MEOD) spectrum of compound **11**

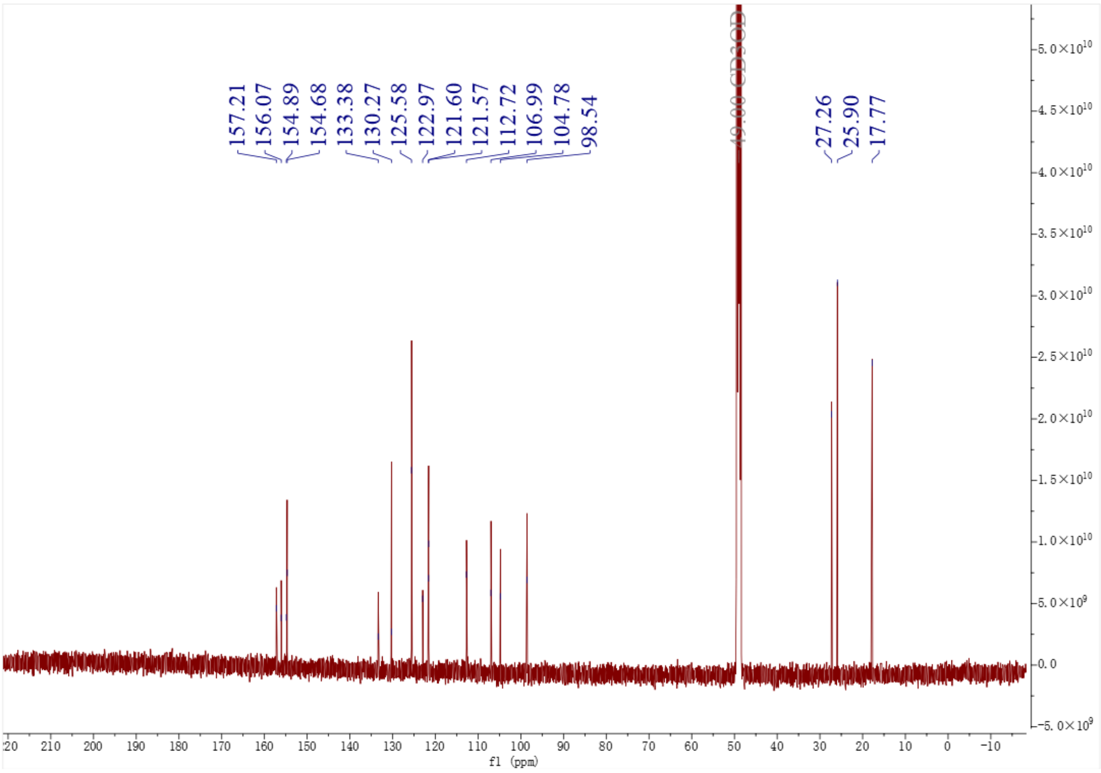

Figure S35: 13C-NMR (126 MHz, MEOD) spectrum of compound **11**

12

Pale yellow powder with a molecular formula of $C_{24}H_{26}O_4$. In its ^{13}C-NMR spectrum, five oxygenated aromatic carbon signals are observed in the low-field region at δC 157.89, 156.86, 156.28, 155.73, and 153.54, consistent with the characteristics of a 6-hydroxybenzofuran skeleton and a 3,5-dihydroxybenzene ring. In its ^{1}H-NMR spectrum, a singlet is present at δH 6.68, and in the ring A region, a pair of ortho spin-coupled proton signals at δH 7.19 and 6.75 indicate that ring A has a 6,7-disubstituted pattern. Both ^{1}H- and ^{13}C-NMR spectra show two sets of characteristic prenyl group signals [δH 5.41, 3.59, 1.69, 1.67 and δH 5.18, 3.48, 1.85, 1.64]. Based on the above spectroscopic analysis and comparison with literature data, the spectroscopic characteristics of this compound are completely consistent with those of Cathafuran B. Therefore, compound 12 was identified as Cathafuran B.

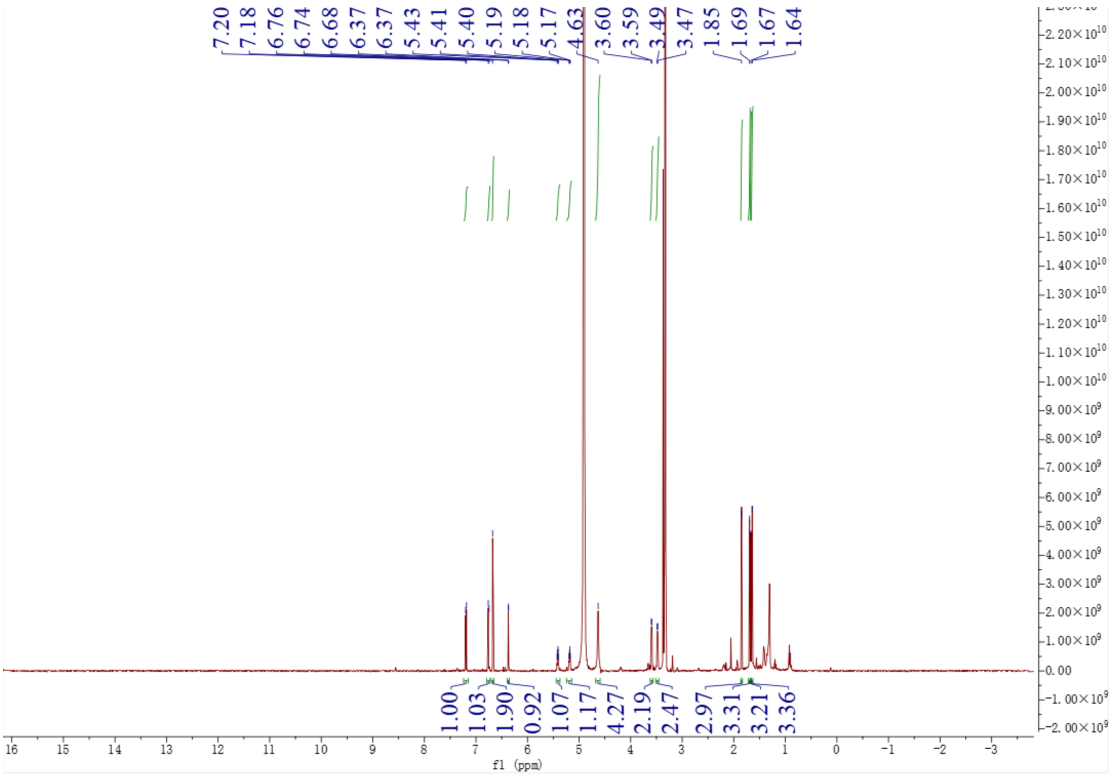

Figure S36: 1H-NMR (500 MHz, MEOD) spectrum of compound **12**

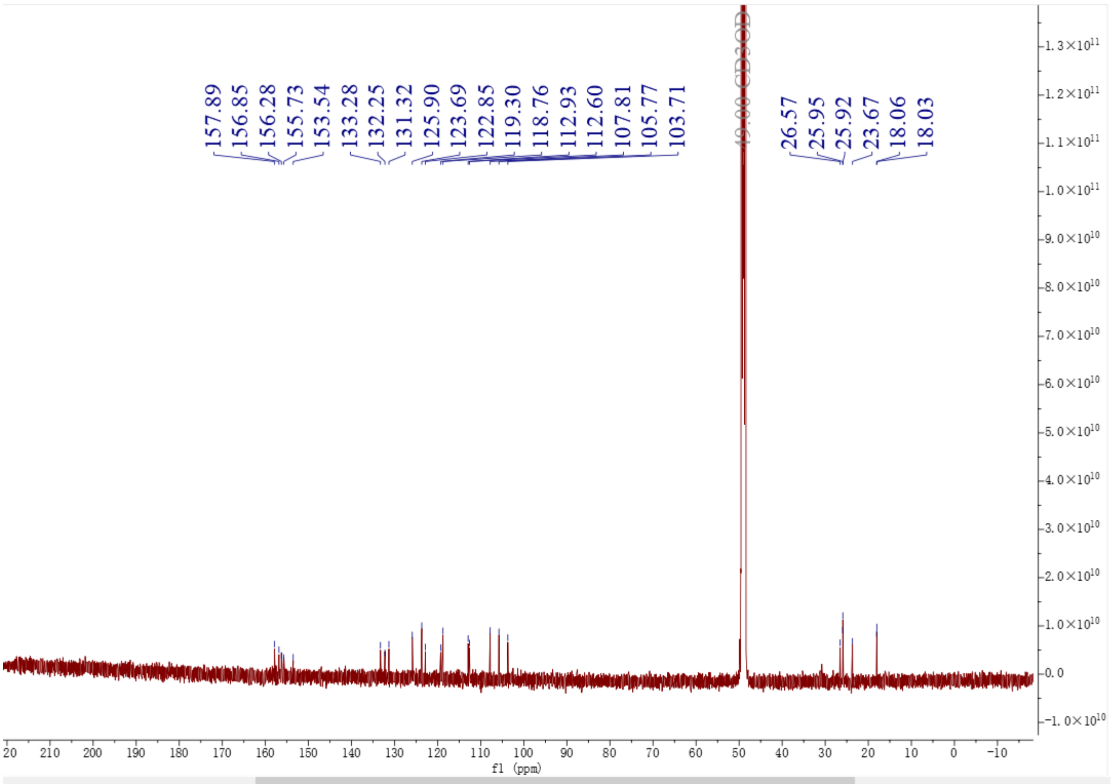

Figure S37: 13C-NMR (126 MHz, MEOD) spectrum of compound **12**

13

Yellow powder with a molecular formula of $C_{20}H_{20}O_6$. In its ^{13}C-NMR spectrum, a dihydroflavonoid ketone carbonyl carbon signal is observed at δC 197.3. In the mid-field region, there are an oxygenated methine carbon at δC 74.8 and an aliphatic methylene carbon at δC 42.6. In the ^1H-NMR spectrum, the proton signals corresponding to these two carbons form an ABX spin coupling system [δH 5.69, 3.11, 2.69], indicating that the core skeleton of the compound is an unsubstituted dihydroflavonoid. The aromatic region of the ^1H-NMR spectrum shows a pair of meta-coupled proton signals on ring A at δH 5.96 and δH 5.95, indicating that ring A has a 5,7-dihydroxy substitution pattern. In the B-ring region, a pair of ortho-coupled proton signals at δH 7.21 and δH 6.49 indicate that the B-ring is 1,2,3,4-tetrasubstituted. An oxygenated quaternary carbon at δC 76.9 and two methyl carbon signals at δC 27.9 and 27.7 in the carbon spectrum indicate the presence of a 2,2-dimethylpyran ring in the molecule. Based on the above spectroscopic features and comparison with literature data, the NMR data of this compound are completely consistent with those of Sanggenon H. Therefore, compound 13 was identified as Sanggenon H.

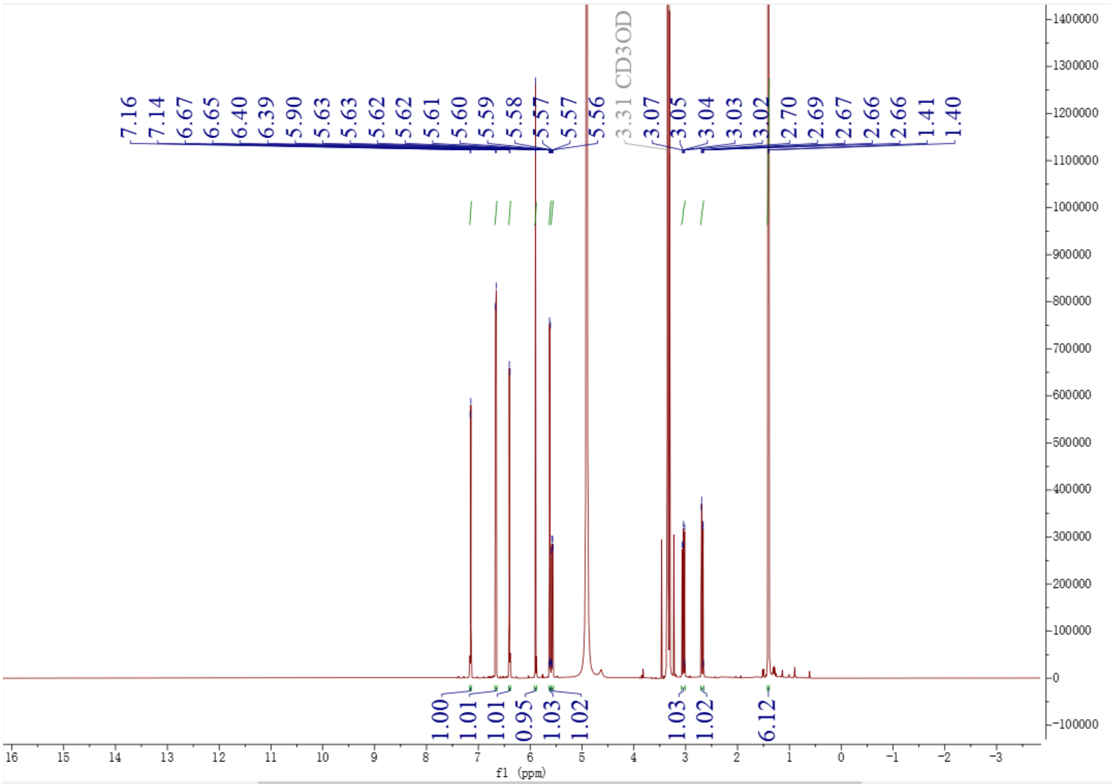

Figure S38: 1H-NMR (600 MHz, MEOD) spectrum of compound **13**

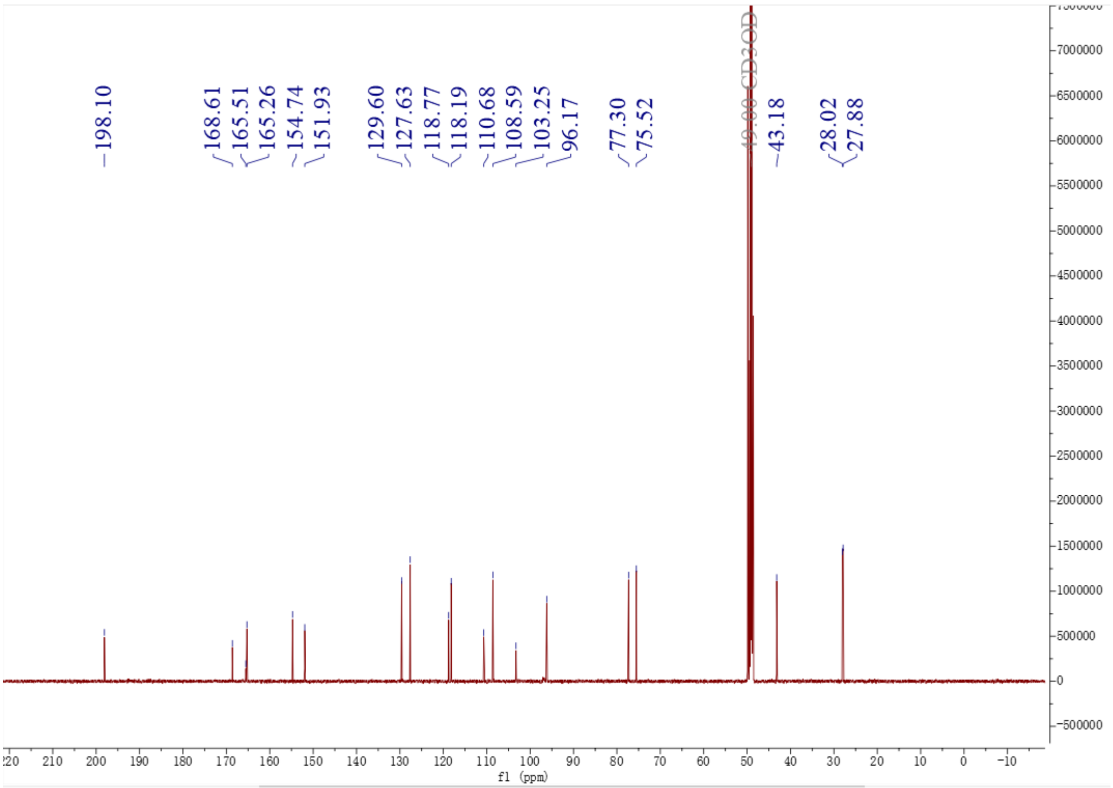

Figure S39: 13C-NMR (151 MHz, MEOD) spectrum of compound **13**

14

White powder with a molecular formula of $C_7H_6O_3$. Its ^{1}H-NMR (600 MHz, CD_3OD) spectrum shows signals at δH 7.88 and δH 6.82, indicating that the compound contains a 1,4-disubstituted benzene ring skeleton. Its ^{13}C-NMR (151 MHz, CD_3OD) spectrum reveals a carbonyl carbon signal at δC 170.1 and an oxygenated aromatic quaternary carbon signal at δC 163.4 in the low-field region, along with three carbon signals at δC 133.0, 122.7, and 116.0 in the mid-field region. Based on the above spectroscopic data analysis and comparison with NMR data reported in the literature, the data of this compound are essentially consistent with those of p-hydroxybenzoic acid. Therefore, compound 14 was identified as p-hydroxybenzoic acid.

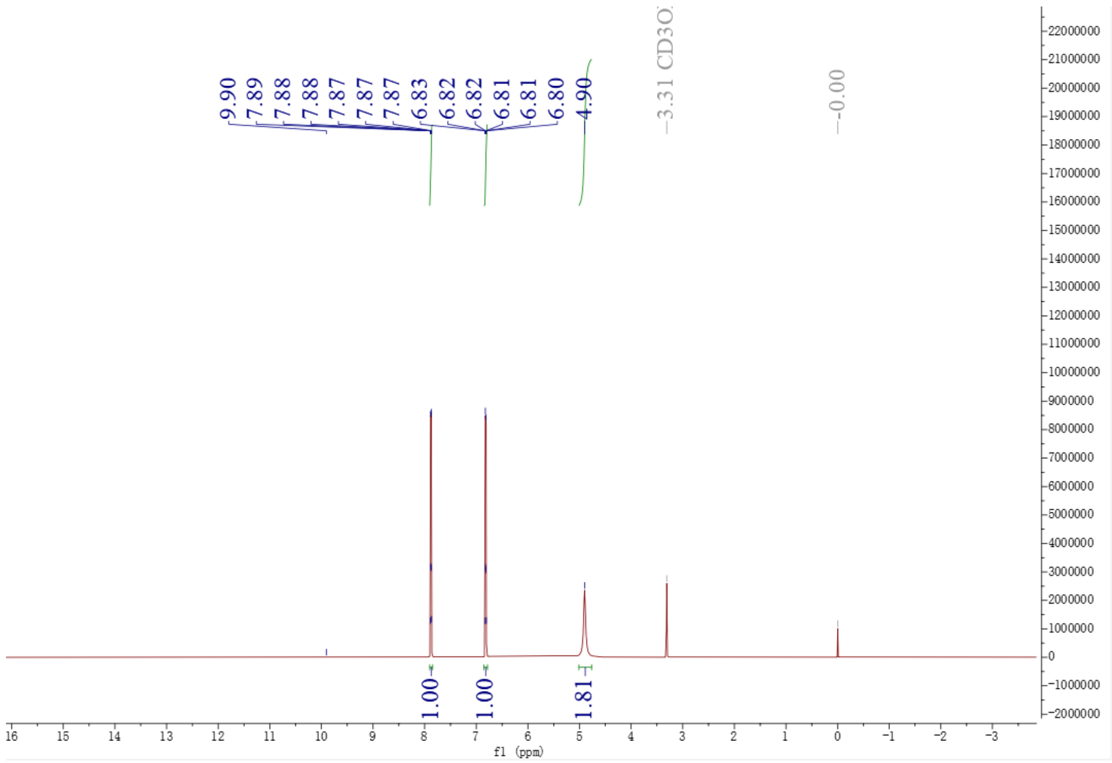

Figure S40: 1H-NMR (600 MHz, MEOD) spectrum of compound **14**

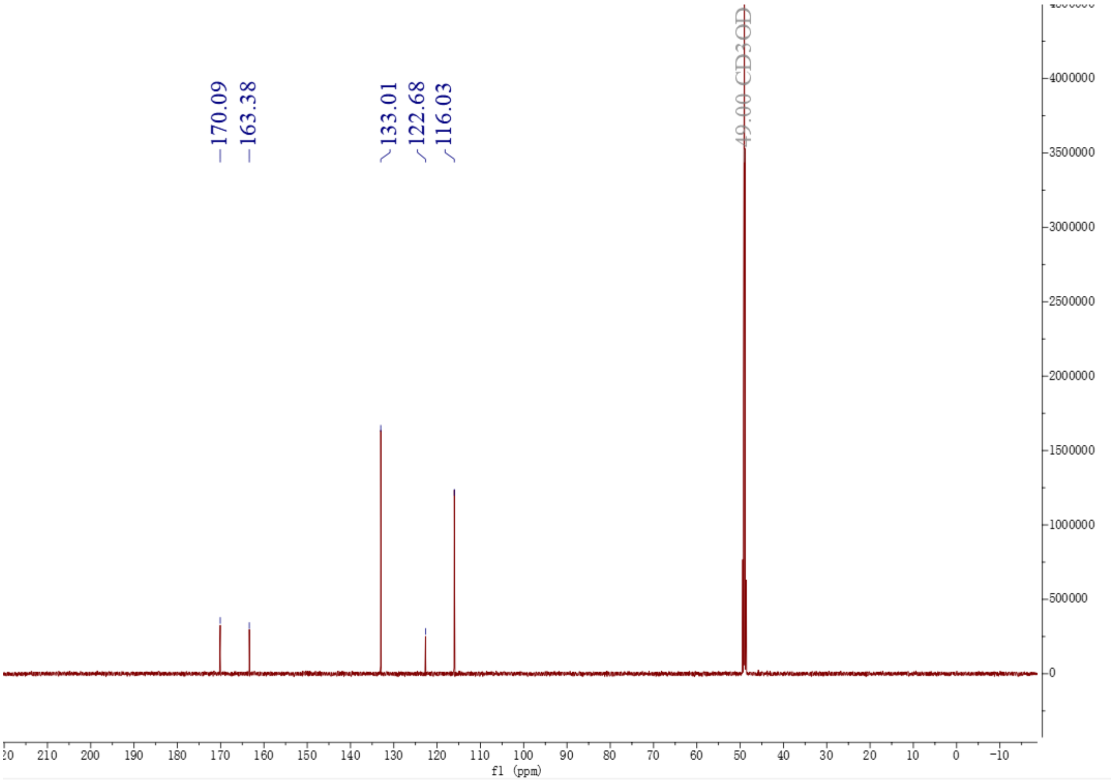

Figure S41: 13C-NMR (151 MHz, MEOD) spectrum of compound **14**

15

White powder with a molecular formula of $C_{30}H_{48}O_3$. Based on its ^{13}C-NMR spectrum and the distribution characteristics of chemical shifts, it can be inferred that this compound is a typical pentacyclic triterpene skeleton. In the low-field region, a distinct carboxyl carbonyl carbon signal is observed at δC 181.6, along with a pair of characteristic trisubstituted double bond carbon signals at δC 139.6 and 126.9. In the mid-field region, an oxygenated methine carbon signal is present at δC 79.7, indicating the presence of a hydroxyl group at the C-3 position of ring A. In the high-field region, seven methyl carbon signals are observed at δC 27.9, 24.1, 21.6, 17.8, 17.7, 16.4, and 16.1. Comparison with literature data shows that the above NMR data are in good agreement with those reported for Ursolic acid. Therefore, compound 15 was identified as ursolic acid.

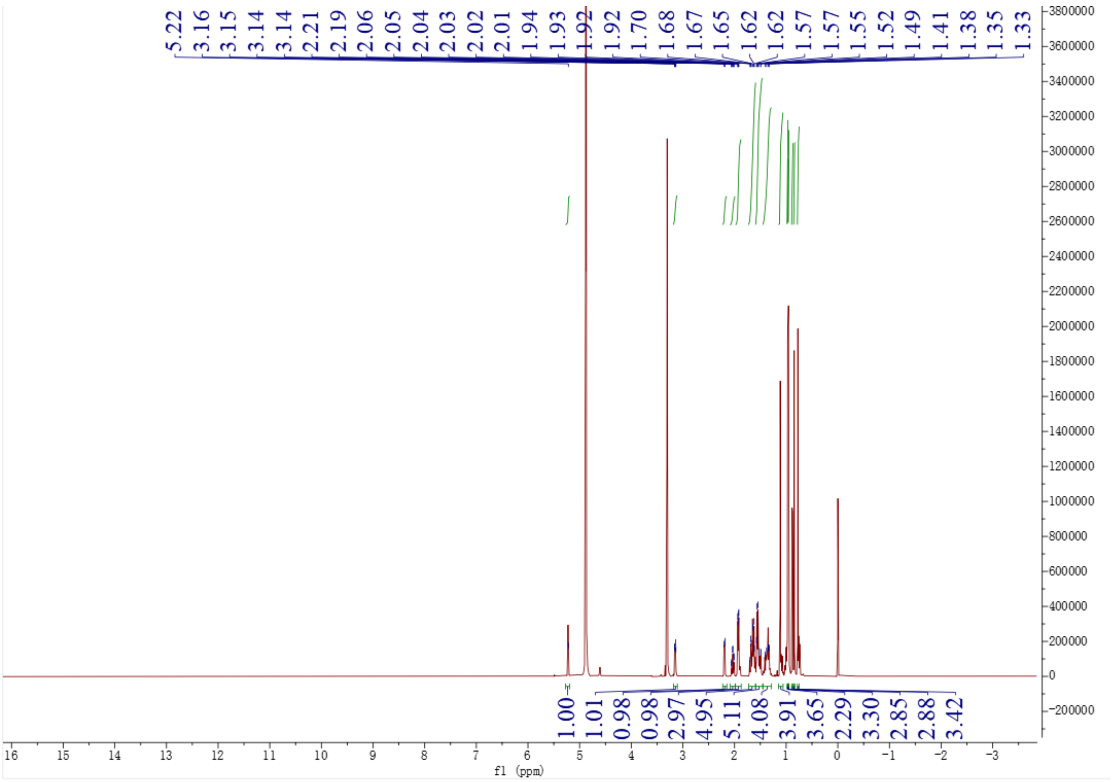

Figure S42: 1H-NMR (600 MHz, MEOD) spectrum of compound **15**

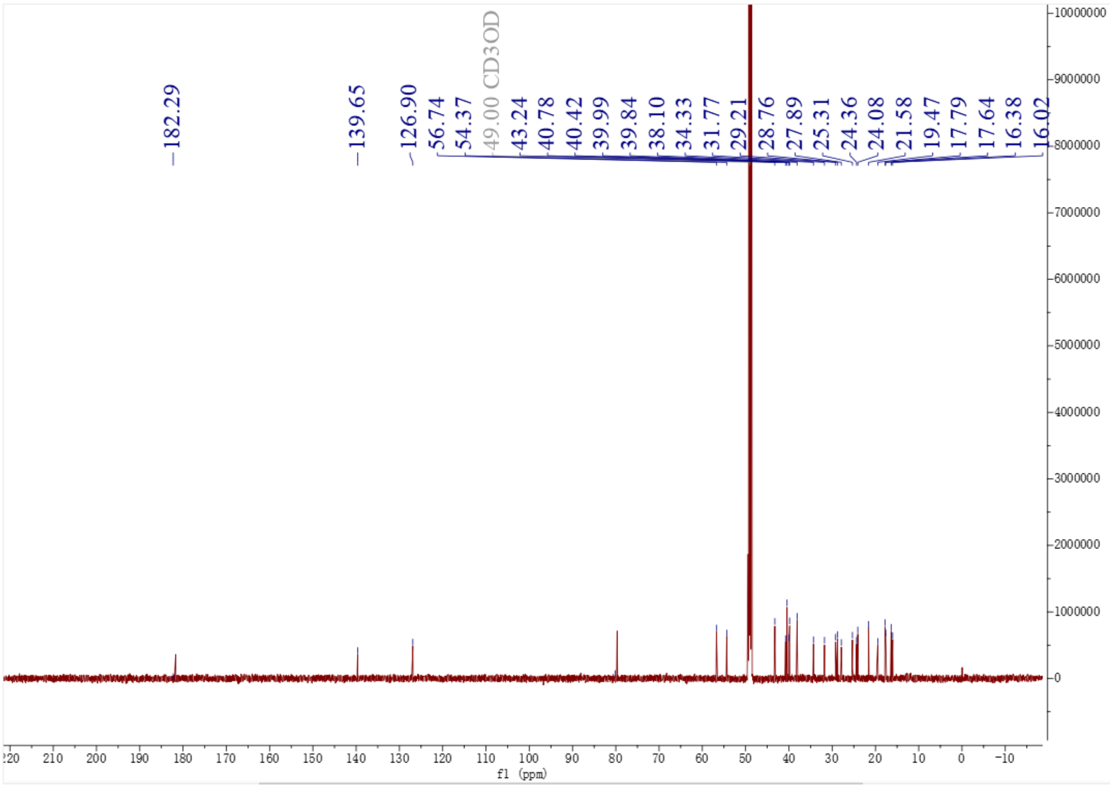

Figure S43: 13C-NMR (151 MHz, MEOD) spectrum of compound **15**

16

Yellow powder with a molecular formula of $C_{20}H_{18}O_7$. Its ^{1}H-NMR (500 MHz, MeOD) spectrum shows an AMX coupling system characteristic of a 1,2,4-trisubstituted benzene ring at δH 7.24, 6.44, and 6.33, along with two meta-coupled aromatic proton signals typical of a phloroglucinol-type substitution pattern at δH 5.83 and 5.71. In the high-field region, a set of characteristic prenyl group signals is observed at δH 5.20, 3.07, 2.72, 1.61, and 1.52. ^{13}C-NMR shows a carbonyl carbon signal at δC 189.0, five carbon signals corresponding to a prenyl group at δC 137.2, 119.0, 32.5, 26.0, and 18.1, along with 12 aromatic carbon signals. Two oxygenated quaternary carbon signals are present at δC 92.4 and 102.6. Based on the comprehensive spectroscopic features and comparison with literature reports, the NMR data of this compound are in good agreement with those reported for Nigragenon E. Therefore, compound 16 was identified as Nigragenon E.

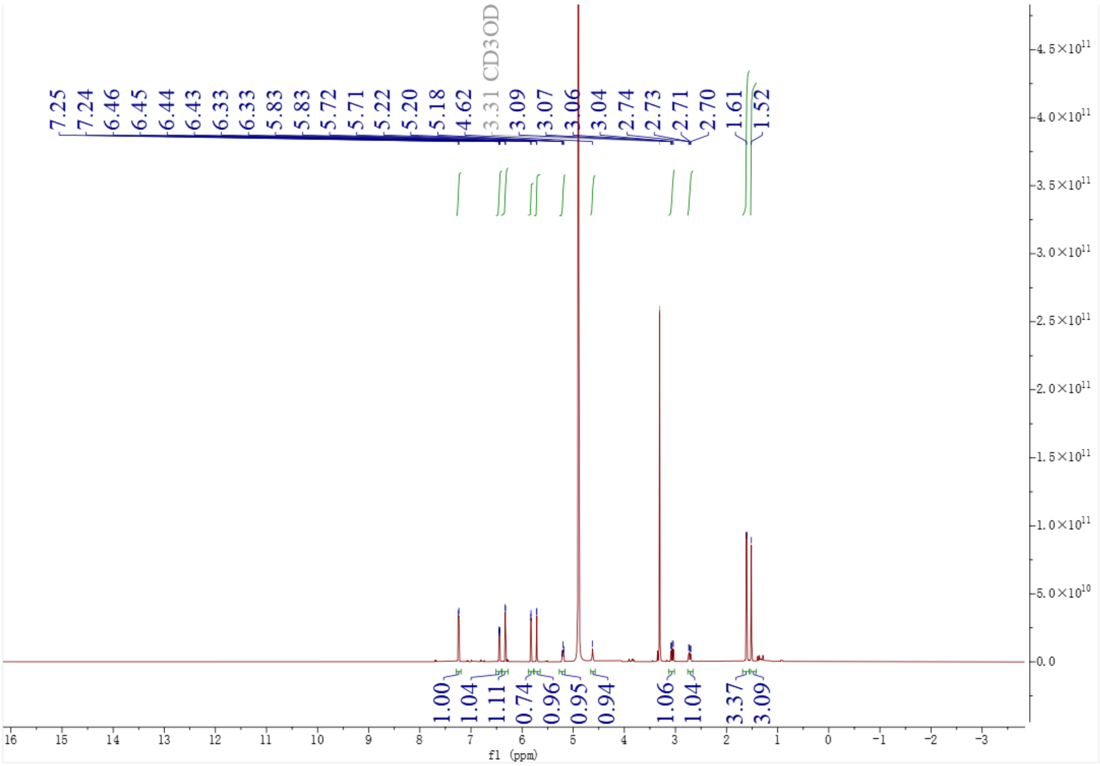

Figure S44: 1H-NMR (500 MHz, MEOD) spectrum of compound **16**

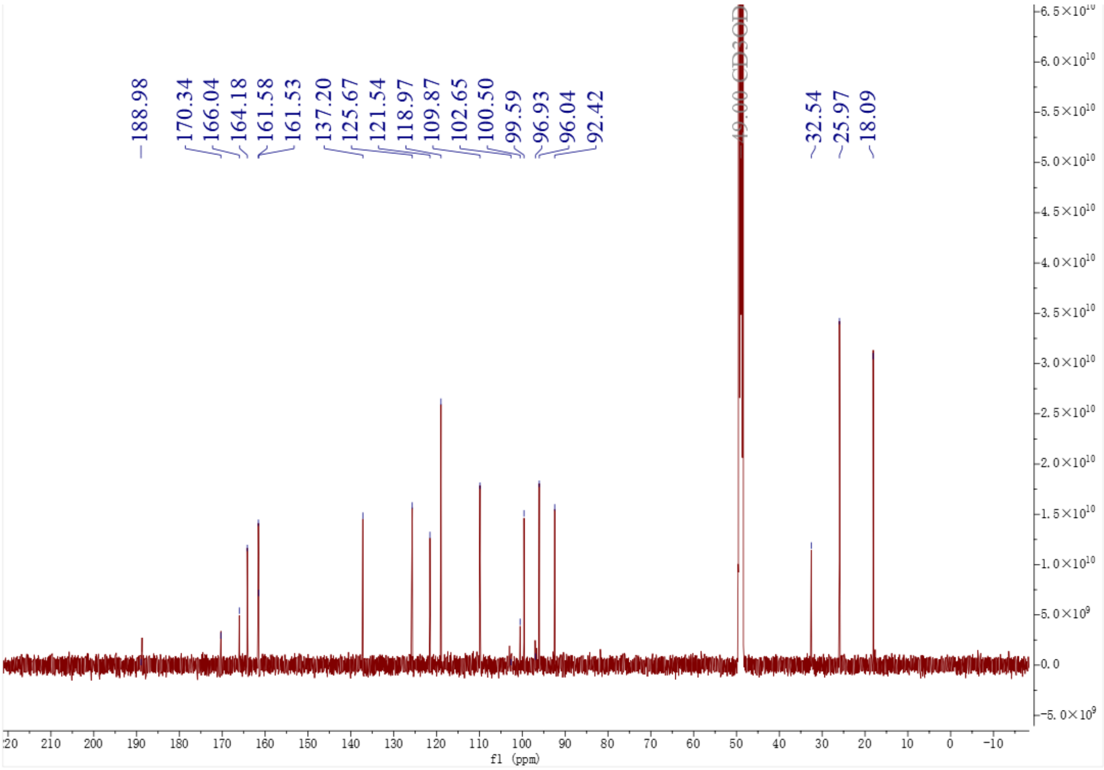

Figure S45: 13C-NMR (126 MHz, MEOD) spectrum of compound **16**

17

Yellow powder with a molecular formula of $C_{25}H_{24}O_6$. Its ^{13}C-NMR spectrum

shows a flavonoid ketone carbonyl carbon signal at δC 183.68 in the low-field region, confirming the presence of a hydroxyl group at the C-5 position. In the aromatic region of its ¹H-NMR spectrum, a pair of meta-coupled proton signals at δH 6.21 and 6.29 are observed on ring A, indicating that ring A has a 5,7-dihydroxy substitution pattern. In the B-ring region, a pair of ortho-coupled proton signals at δH 7.05 and 6.51 indicate that the B-ring is a 1,2,3,4-tetrasubstituted system. In the high-field region, two singlet methyl signals are present at δH 1.55 and 1.38. Combined with a pair of trisubstituted double bond signals at δC 132.85 and 122.51, as well as signals at δH 3.01 and δC 24.70, these indicate the presence of a prenyl group in the molecule. Based on the above spectroscopic features and comparison with literature data, the NMR data of this compound are consistent with those of Kuwanon A. Therefore, compound 17 was identified as Kuwanon A.

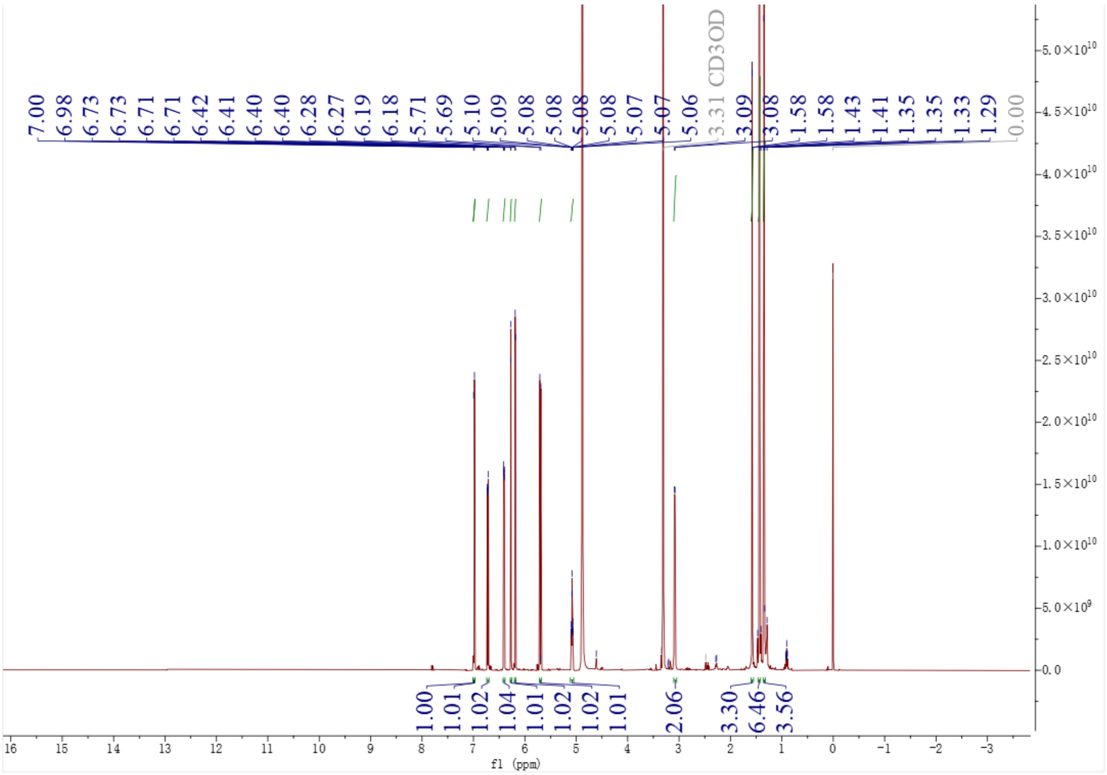

Figure S46: 1H-NMR (500 MHz, MEOD) spectrum of compound **17**

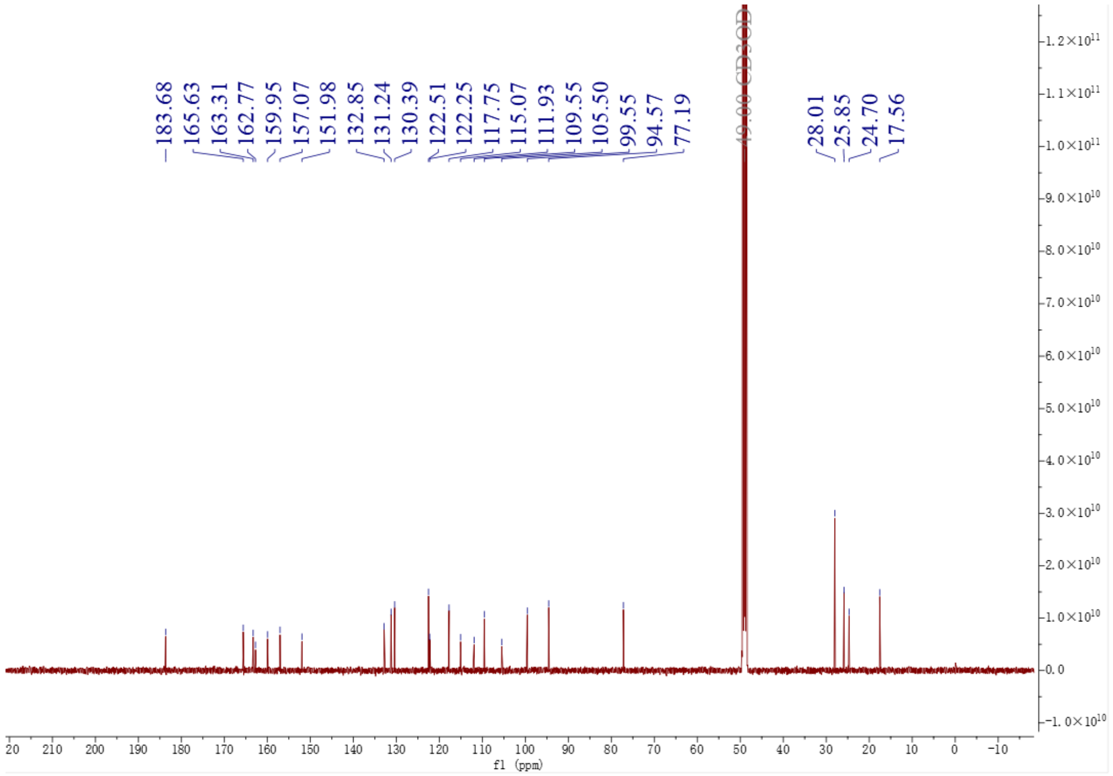

Figure S47: 13C-NMR (126 MHz, MEOD) spectrum of compound **17**

18

Pale yellow powder with a molecular formula of $C_{14}H_{10}O_4$. In the low-field region, multiple oxygenated aromatic quaternary carbon signals are observed at δC 158.56, 155.85, 155.45, and 154.72. Combined with the furan ring quaternary carbon signal at δC 154.72, it is preliminarily deduced that the compound has a 2-arylbenzofuran skeleton. In the aromatic region of the ^{1}H-NMR spectrum, two independent spin coupling systems are present. Among them, δH 6.25 exhibits characteristic meta-coupled splitting, together with overlapping multiplets at δH 6.79–6.71, forming a 1,3,5-trisubstituted symmetric benzene ring system. δH 7.35 is a characteristic ortho-coupled proton, attributed to the benzofuran ring A. Based on the above spectroscopic features and comparison with literature reports, this compound is found to be in good agreement with the known compound Moracin M. Therefore, compound 18 was identified as Moracin M.

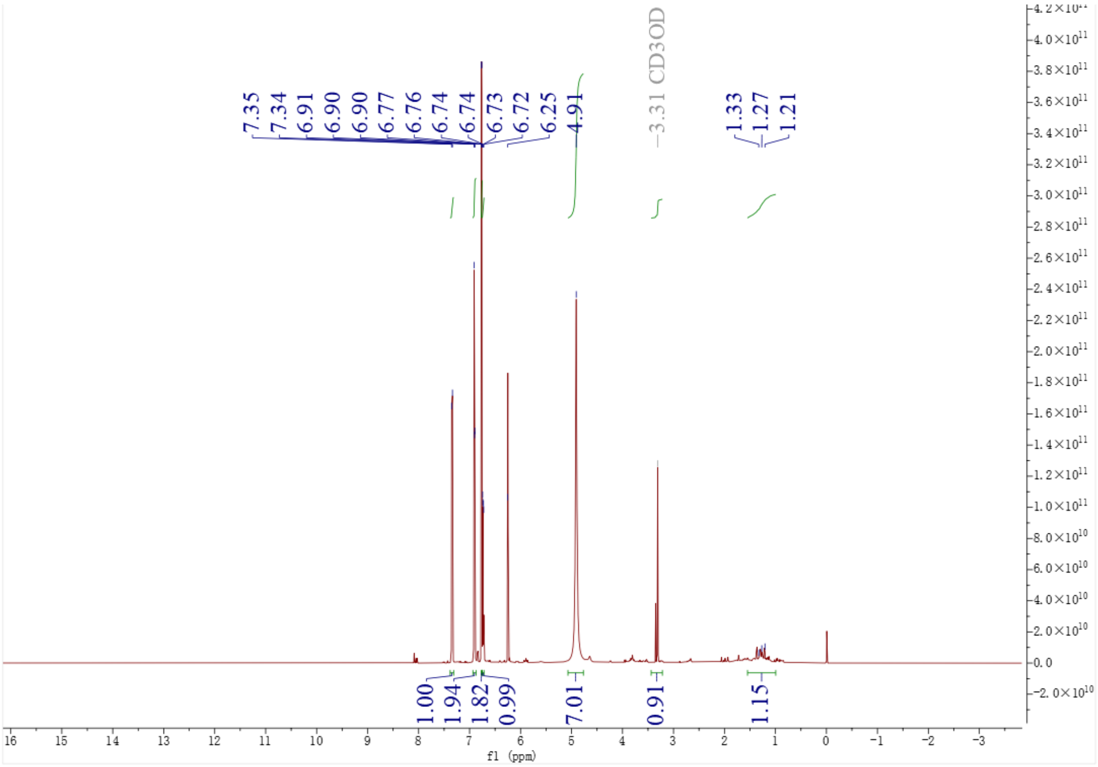

Figure S48: 1H-NMR (500 MHz, MEOD) spectrum of compound **18**

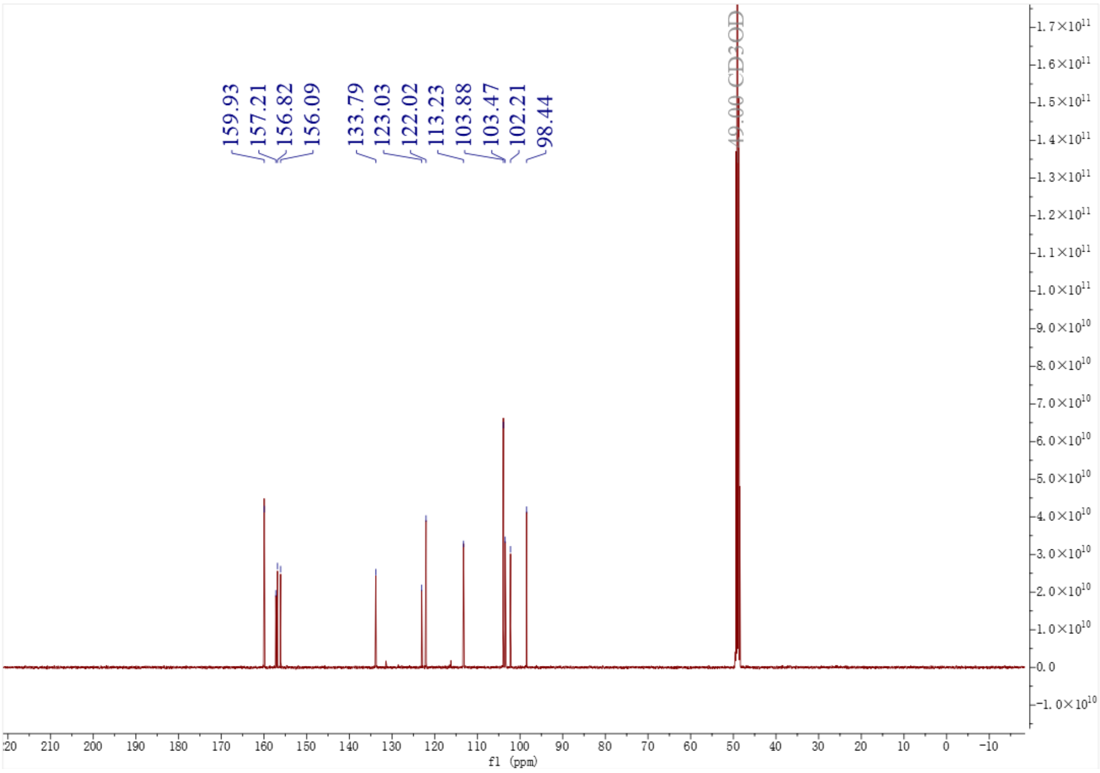

Figure S49: 13C-NMR (126 MHz, MEOD) spectrum of compound **18**

19

Yellow powder with a molecular formula of $C_{25}H_{24}O_6$. Its ^{13}C-NMR spectrum shows a flavonoid ketone carbonyl carbon signal at δ 183.90 in the low-field region. The B-ring signals at δC 157.98 and δC 160.50 are shifted downfield, indicating a 2',4'-dihydroxy substitution pattern on the B-ring. In the high-field region, four singlet methyl signals are observed at δH 1.59, 1.44, 1.44, and 1.41. Combined with trisubstituted double bond signals at δ 132.45 and 122.73 and an aliphatic methylene signal at δH 3.10 and δC 24.90 in the carbon spectrum, these indicate the presence of a prenyl group in the molecule. The carbon signal of the core skeleton at δC 122.06 is shifted downfield and corresponds to a quaternary carbon, suggesting that the prenyl group is substituted at the C-3 position. In the ^{1}H-NMR spectrum, signals at δH 6.60 and 5.60, together with an oxygenated quaternary carbon at δC 79.14 and two methyl carbons in the carbon spectrum, indicate the presence of a 2,2-dimethylpyran ring in the molecule. The ring A carbon signals at δC 163.61 and δC 108.04 are both quaternary carbons, indicating that this pyran ring is fused at the C-7 and C-8 positions of the core skeleton. Based on the above spectroscopic features and comparison with literature data, the data of this compound are consistent with those of Morusin.

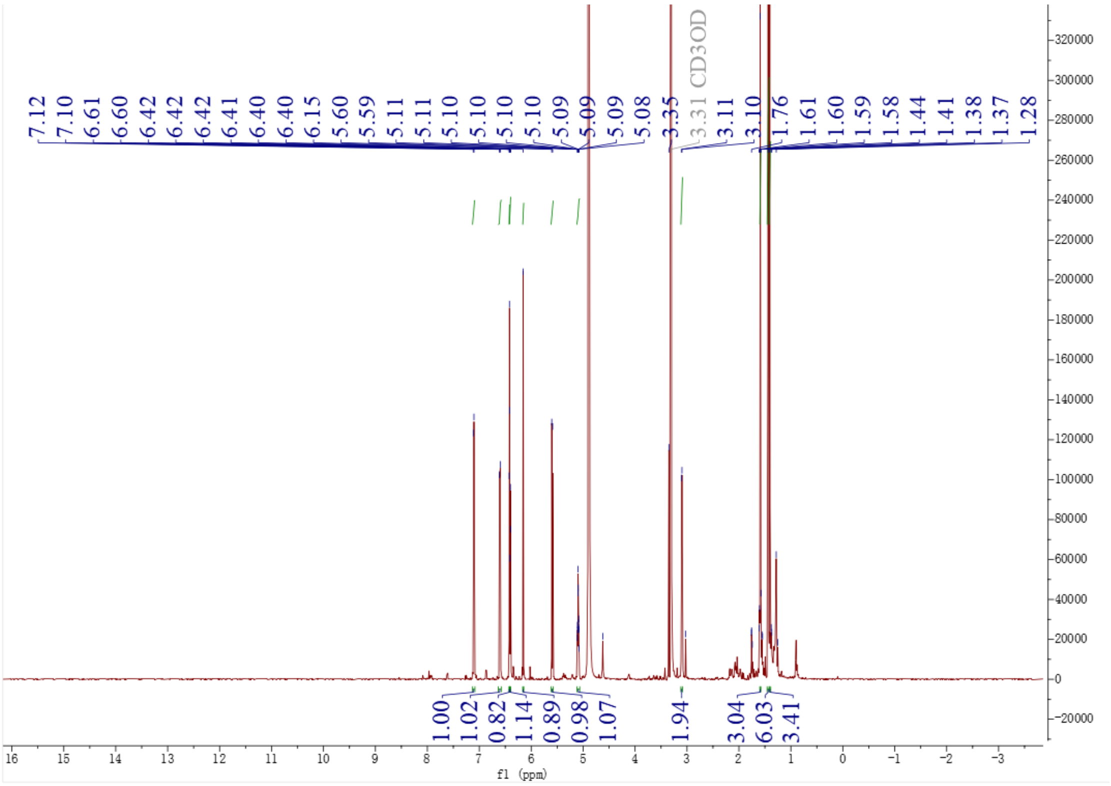

Figure S50: 1H-NMR (600 MHz, MEOD) spectrum of compound **19**

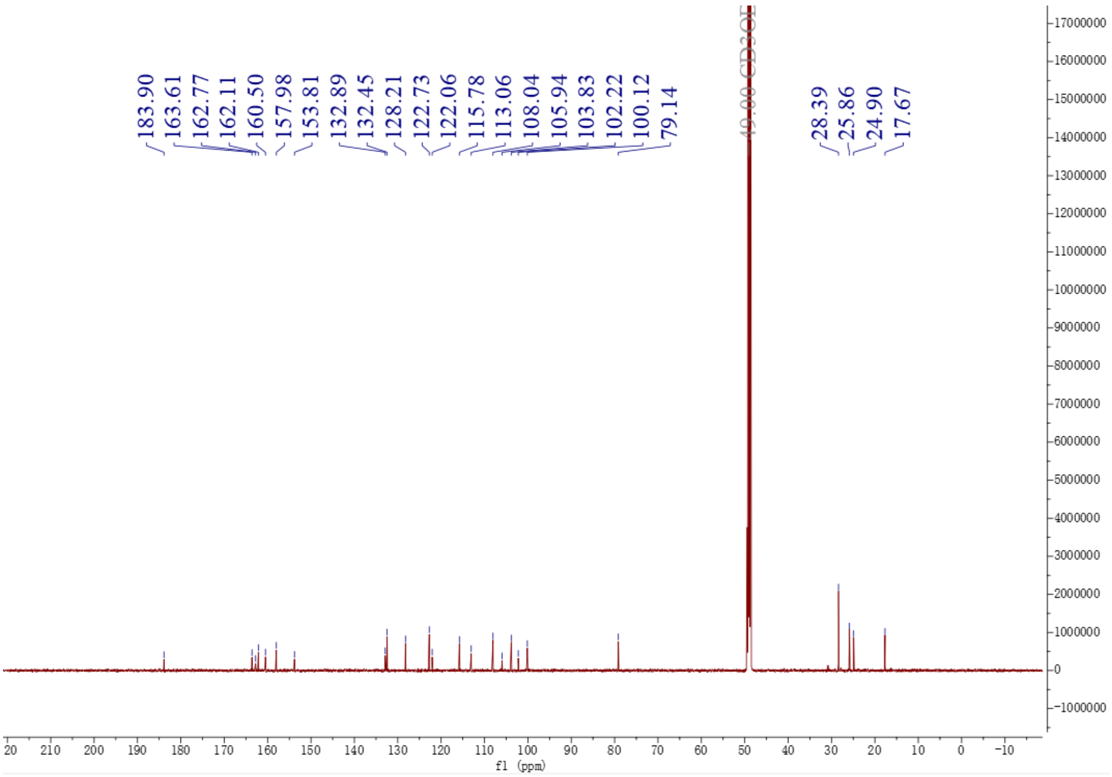

Figure S51: 13C-NMR (151 MHz, MEOD) spectrum of compound **19**

20

Yellow powder with a molecular formula of $C_{27}H_{22}O_6$. In its ^{13}C-NMR spectrum, an oxygenated carbon signal is present at δC 72.54 in the mid-field region. Combined with analysis of the ^{1}H-NMR spectrum, a signal at δH 6.86 in the low-field region indicates a 2-arylbenzofuran skeleton. Signals at δH 7.32, 6.71, and 6.86 suggest that ring A has a 6-hydroxy substitution pattern. The B-ring exhibits a set of meta/ortho-coupled proton signals at δH 6.93, 6.33, and 6.21, indicating that this compound has a 1,2,4-trisubstituted benzene ring system. Based on the above spectroscopic features and comparison with literature data, the NMR data of this compound are consistent with those of Mulberrofuran H. Therefore, compound 20 was identified as Mulberrofuran H.

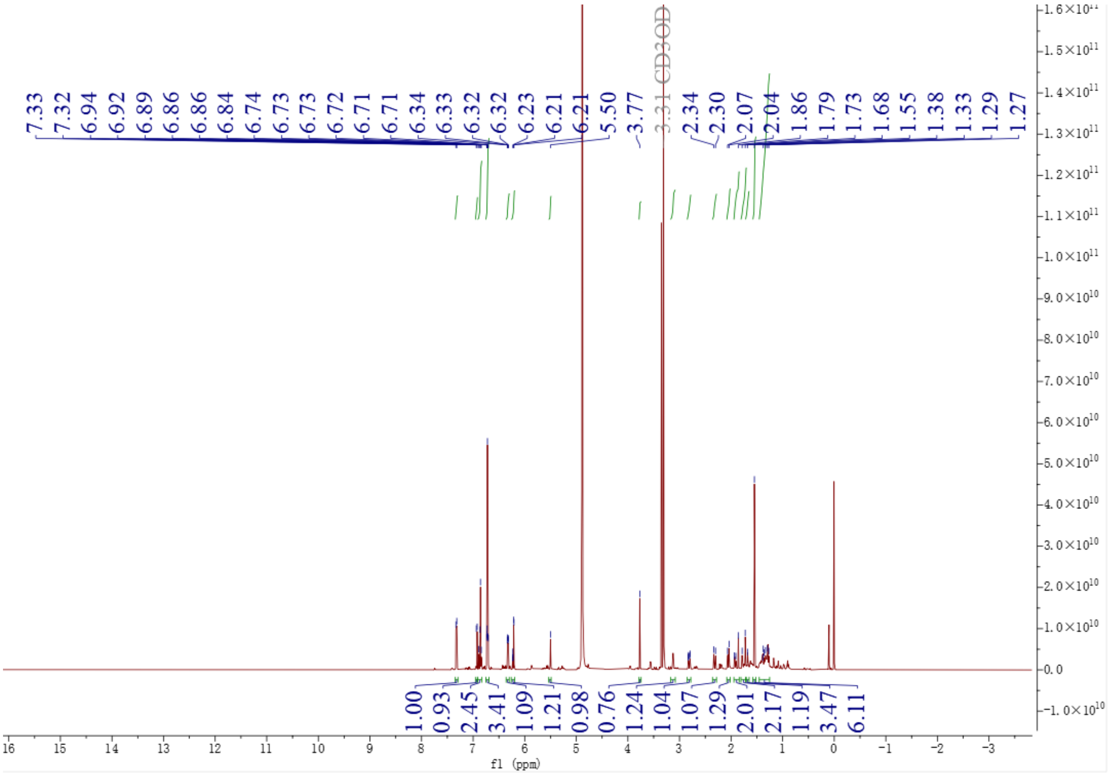

Figure S52: 1H-NMR (500 MHz, MEOD) spectrum of compound **20**

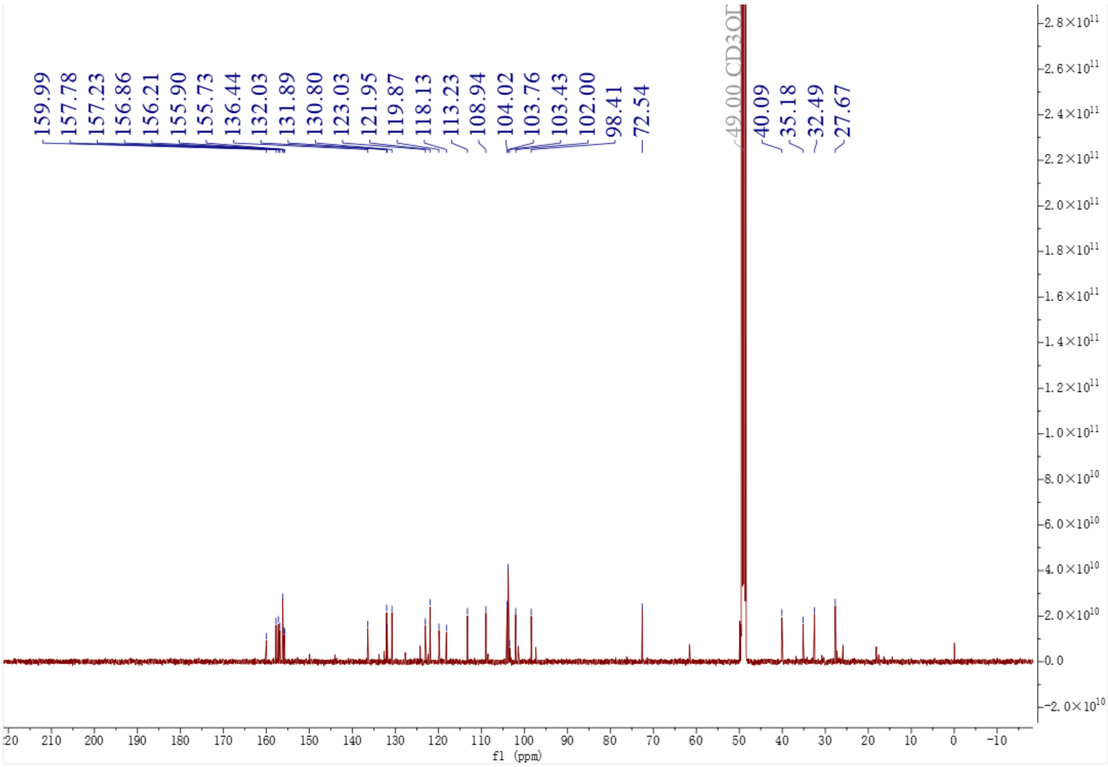

Figure S53: 13C-NMR (126 MHz, MEOD) spectrum of compound **20**

21

Yellow powder with a molecular formula of $C_{25}H_{24}O_6$. In its ^{13}C-NMR spectrum, a dihydroflavonoid ketone carbonyl carbon signal is observed at δC 197.98 in the low-field region. In the mid-field region, a pair of dihydroflavonoid core carbon signals are present: an oxygenated methine carbon at δC 75.31 and an aliphatic methylene carbon at δC 43.19. In the ^{1}H-NMR spectrum, the proton signals corresponding to these two carbons form a spin coupling system at δH 5.59 and 3.22, confirming the basic dihydroflavonoid skeleton. In the aromatic region of its ^{1}H-NMR spectrum, a broad singlet with an integral equivalent to 2H is present at δH 5.89, corresponding to δC 97.10 and δC 96.18 of ring A, indicating that ring A has a 5,7-dihydroxy substitution pattern. In the B-ring region, an isolated singlet proton signal is observed at δH 7.09, indicating that the B-ring is pentasubstituted. Based on the above spectroscopic features and comparison with literature data, the NMR data of this compound are consistent with those of Sanggenol O. Therefore, compound 21 was identified as Sanggenol O.

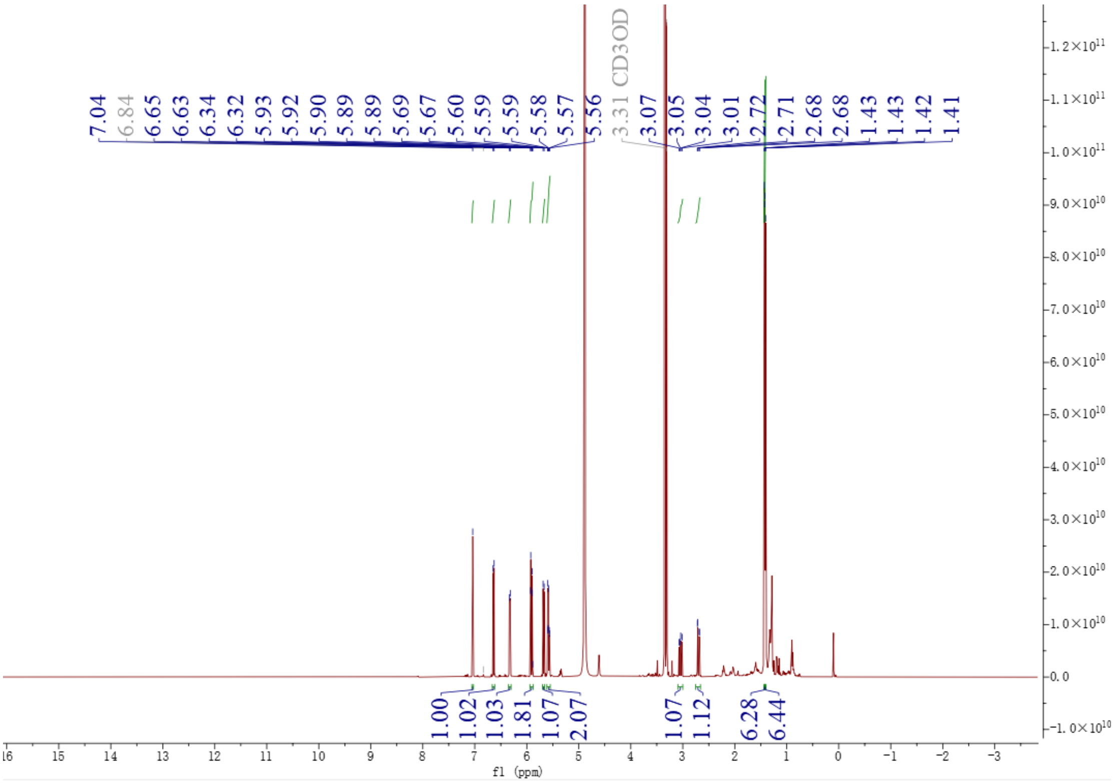

Figure S54: 1H-NMR (500 MHz, MEOD) spectrum of compound **21**

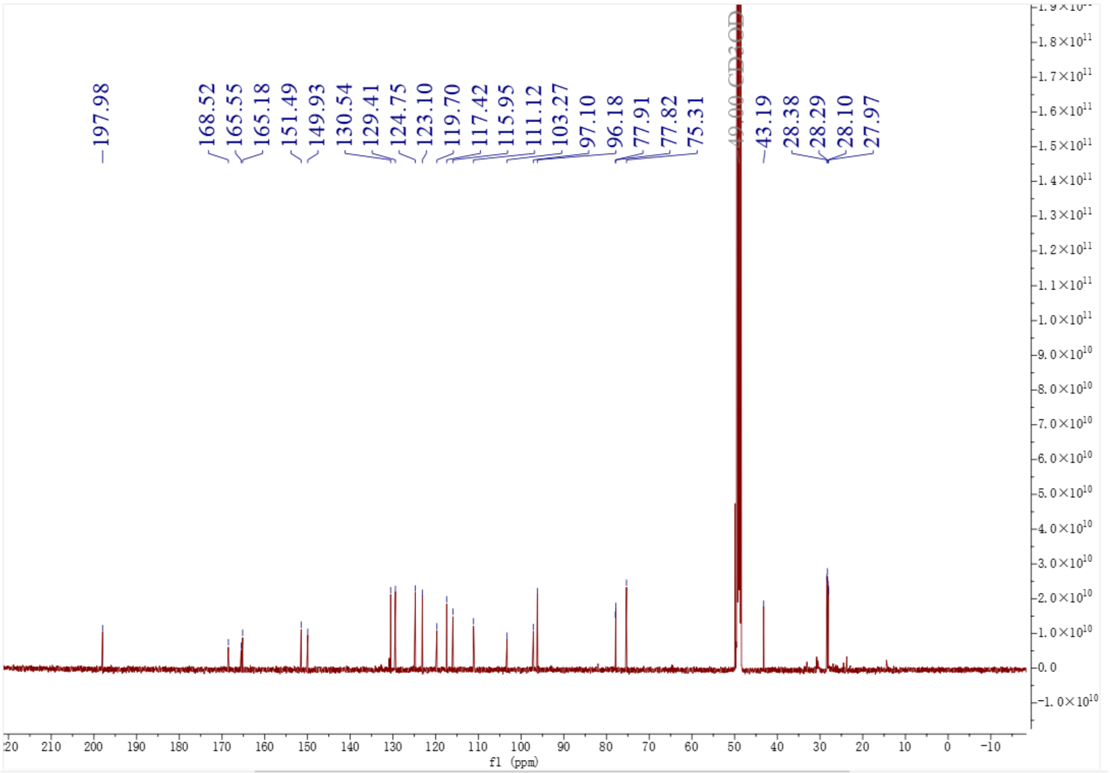

Figure S55: 13C-NMR (126 MHz, MEOD) spectrum of compound **21**

22

Yellow powder with a molecular formula of $C_{40}H_{36}O_{12}$ is presumed to be a typographical error; corrected to $C_{40}H_{36}O_{12}$ based on structural context). The ^{13}C-NMR spectrum shows characteristic α,β-unsaturated ketone carbonyl at δC 188.83 and a hemiketal oxygenated quaternary carbon at δC 92.0 in the low-field region, suggesting that the core skeleton is a 2-hydroxyflavanone. The aromatic region displays abundant signals, comprising four typical polyhydroxy-substituted benzene ring systems corresponding to the flavonoid A/B rings, a benzoyl group, and a resorcinol group. Additionally, δC 208.8 indicates the presence of an isolated aliphatic ketone carbonyl in the side chain. In the mid- to high-field region, a characteristic set of prenyl group signals is observed at δC 32.38, 118.84, 137.21, 18.13, and 26.04, along with signals for a six-membered aliphatic ring containing a double bond and free methyl groups at δC 35.97, 123.67, 134.73, 32.79, and 33.99, as well as a methyl signal at δC 22.66. Based on the comprehensive information above and comparison with literature data, compound 22 was identified as Sanggenon C.

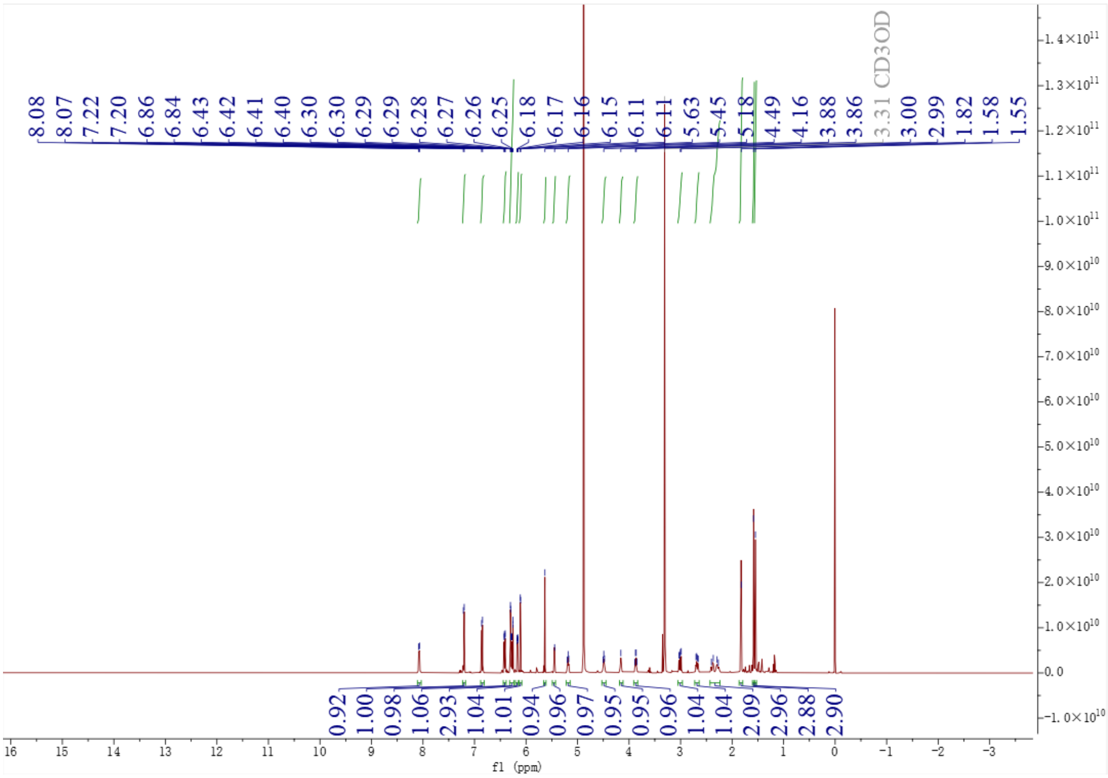

Figure S56: 13C-NMR (126 MHz, MEOD) spectrum of compound **22**

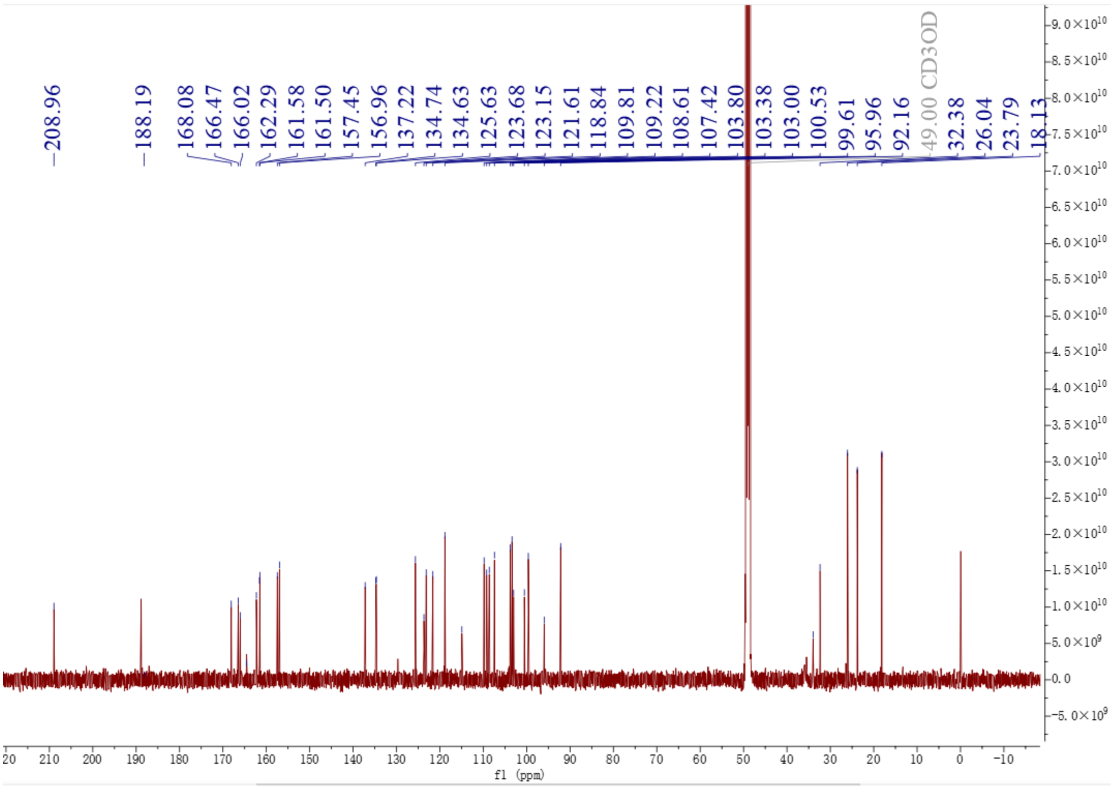

Figure S57: 13C-NMR (126 MHz, MEOD) spectrum of compound **22**
